# A Lewis Acid-Promoted Michael Addition and Ring-Expansion Cascade for the Construction of Nitrogen-Containing Medium-Sized Rings

**DOI:** 10.3390/molecules28041650

**Published:** 2023-02-08

**Authors:** Jiaming Wang, Jia Li, Changgui Zhao

**Affiliations:** Key Laboratory of Radiopharmaceuticals, Ministry of Education, College of Chemistry, Beijing Normal University, Beijing 100875, China

**Keywords:** medium-sized rings, ring expansion, Michael addition, azadienes, eight-membered lactams

## Abstract

A Lewis acid-promoted annulation of azadienes and cyclobutamines was developed. This reaction proceeded through Michael addition and ring-expansion cascade, affording the corresponding nitrogen-containing medium-sized rings with a broad scope in moderate to high yields. The catalytic asymmetric version of this reaction has also been explored using a chiral base.

## 1. Introduction

**Figure 1:**
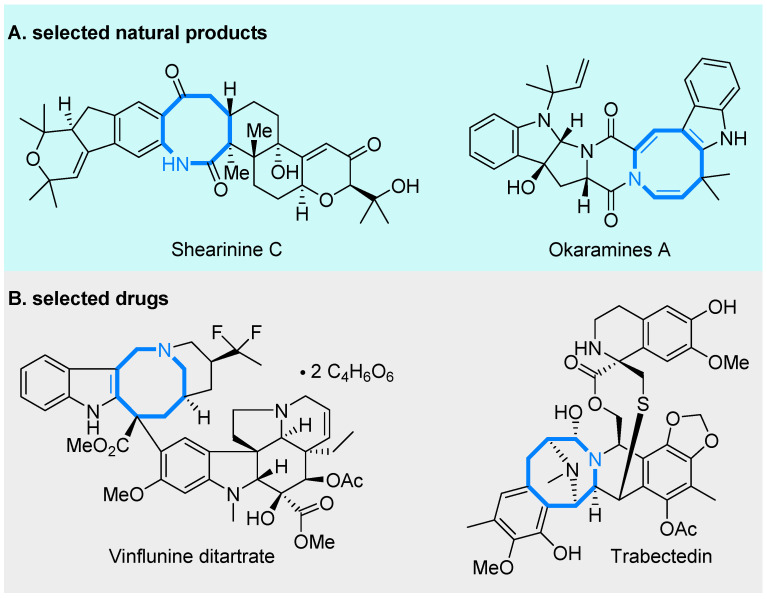
Selected examples of biologically important molecules with a nitrogen-containing medium-sized ring.

Medium-sized rings refer to structures containing from 8 to 11 carbons or heteroatoms. These scaffolds represent a key element in numerous compounds endowed with diverse biological activities [1,2,3,4,5]. Among them, nitrogen-containing medium-sized rings are widely present in natural products (Figure 1A) [6,7]. However, despite their occurrence in some therapeutically important molecules (Figure 1B) [8,9,10], nitrogen-containing medium-sized rings are under-presented among the current clinically approved drugs [11,12]. In contrast, their analogs such as pyrrolidine, piperidine and nitrogen-containing macrocycles are commonly found in marketed drugs [13]. Although various factors contribute to the progression of leads that ultimately become clinically approved for use as prescription drugs [14,15,16,17], the lack of synthetic approaches may limit the drug development process based on privileged structures involving nitrogen-containing medium-sized rings [12,18,19,20]. Consequently, there is an urgent demand to develop efficient protocols to access libraries of nitrogen-containing medium-sized rings as candidates for drug discovery.

**Scheme 1:**
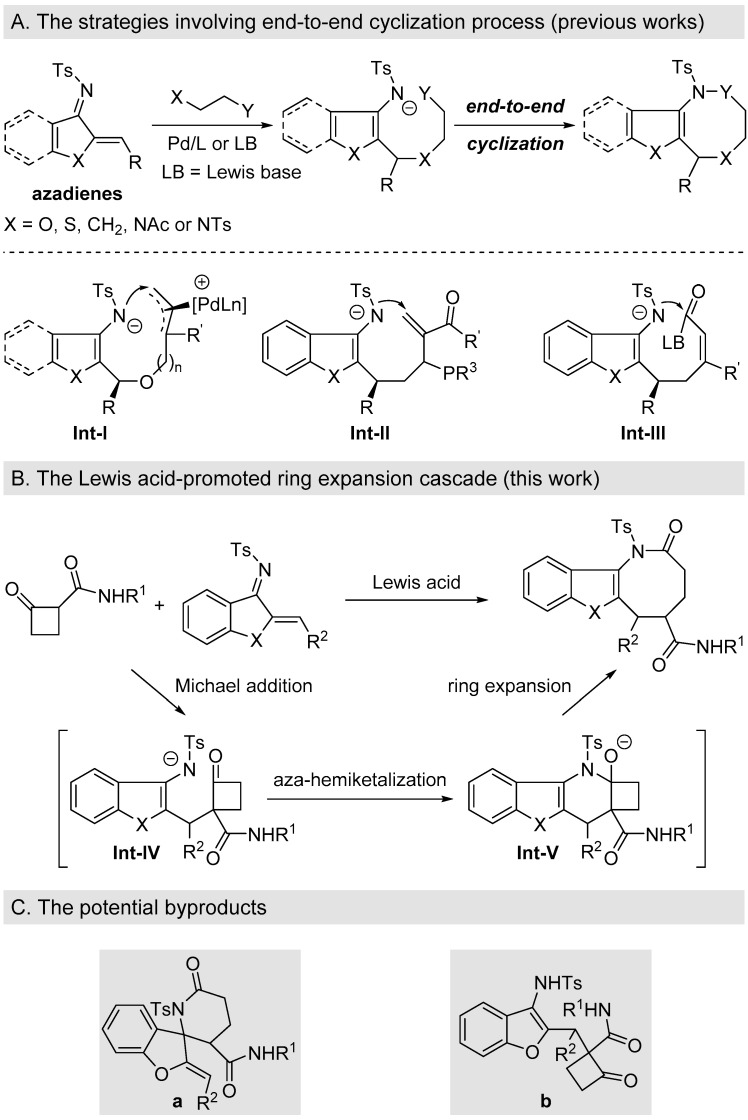
Construction of nitrogen-containing medium-sized rings using azadienes as substrate; Ac = acetyl, Ts = 4-toluenesulfonyl.

In recent years, the synthesis of nitrogen-containing medium-sized rings has attracted considerable attention; significant efforts have been devoted toward their synthesis [21,22,23,24,25,26,27,28,29,30,31,32,33,34,35]. Particularly, azadienes, which act as special *α,β*-unsaturated imines, have been successfully used as effective four-atom synthons to produce nitrogen-containing medium-sized rings through a formal high-order [4 + n] annulation process [36,37,38,39]. For example, the palladium-catalyzed [4 + 4] [40,41], [5 + 4] [42,43,44,45,46] or [6 + 4] [47] annulation established by Zhao, Yao, Lin and co-workers using pyrrole-, benzofuran- or indole-fused azadienes as substrates have proven to be efficient in assembling these frameworks. In 2017, Lu and colleagues disclosed an elegant phosphine-catalyzed enantioselective formal [4 + 4] annulation of azadienes for the synthesis of azocanes [48]. Very recently, Chen further explored the reactivity of azadienes and developed a cinchona alkaloid-catalyzed [4 + 4] annulation for the synthesis of eight-membered lactams [49] (Scheme 1A). Notably, although the above impressive approaches are straightforward for accessing nitrogen-containing medium-sized rings, limitations are observed such as the fact that these strategies rely on an end-to-end cyclization process, that is, nucleophilic attack of the nitrogen atom to the Pd-*π*-allyl moiety (Scheme 1A, **Int-I**), Michael addition of the sulfonamide onto the electron-deficient site of the alkene (Scheme 1A, **Int-II**) and lactamization (Scheme 1A, **Int-III**). Overall, these approaches still suffer from the entropic constraints and unfavorable enthalpic changes due to destabilizing transannular interactions in the medium-sized-ring formation step [50].

Ring expansion of readily available smaller ring systems provides an appealing way to assemble nitrogen-containing medium-sized rings [23,51,52,53,54,55]. Typically, the expansion of a smaller ring to ‘grow’ a medium-sized ring involves the release of a certain degree of instability or formation of a more stable chemical bond, which results in an energy-lowering transformation [28,29,56,57,58,59,60,61,62,63,64,65,66]. The kinetic changes in the difficult end-to-end cyclization approaches are avoided [20]. To the best of our knowledge, the use of azadienes for the construction of nitrogen-containing medium-sized rings through a ring-expansion process remains elusive in the literature. Given the interest of our group in the synthesis of medium-sized rings [4,26,67], we herein present the first example of Lewis acid-promoted ring-expansion approaches for azadienes and cyclobutamines for the construction of eight-membered lactams (Scheme 1B). We anticipated that the chemo- and regioselectivities of this approach would be challenging, as the 1,2-addition of a nucleophile to azadienes has been observed previously, which delivers a spiro-lactam side product (Scheme 1C, **a**) [40,68,69]. Additionally, the electron deficiency characteristic of the 4-toluenesulfonyl group and steric hindrance of the quaternary carbon at the *α*-position of the carbonyl might prevent the aza-hemiketalization reaction (Scheme 1B, **Int-IV**), which leads to another acyclic side product (Scheme 1C, **b**).

## 2. Results and Discussion

We commenced the investigation by employing azadiene **1a** and cyclobutanone **2a** as model substrates to examine the Michael addition/ring-expansion process. For our diligence, the desired eight-membered lactam **3aa** was obtained in 17% yield when the reaction was performed in dichloromethane as a solvent at room temperature with 2.0 equiv. of triethylamine as the base (Table 1, entry 1). Screening of the organic base including 4-dimethylaminopyridine, *N,N*-diisopropylethylamine and 1,5-diazabicyclo[4.3.0]-5-nonene did not increase the yields (Table 1, entries 2–4), whereas an inorganic base such as potassium carbonate or sodium bicarbonate improved the reaction efficiency (Table 1, entries 5–7). The use of a stronger base to replace potassium carbonate resulted in decomposition of the cyclobutanone **2a** (Table 1, entries 8–9). It is important to note that acyclic side product **4aa** was identified as a major by-product, which was formed through 1,4-addition without further aza-hemiketalization and a ring-expansion sequence. Lewis acids were previously proven to be valid to facilitate aza-hemiketalization [70,71]; a catalytic amount of various Lewis acids were explored. Gratifyingly, the addition of Lewis acids switched the chemoselectivity (Table 1, entries 10–13). A significantly improved yield was obtained when Mg(OTf)_2_ was used, and the formation of the undesired by-product **4aa** was inhibited (54%, Table 1, entry 12).

Subsequently, the survey of the solvent was performed, and toluene was found to be less effective, giving a product with a poor yield (Table 1, entry 17). The yield increased to 63% when 1,2-dichloroethane was used as the solvent (Table 1, entry 15). Additionally, reducing the temperature to −5 °C and prolonging the reaction time to 48 h promoted the aza-hemiketalization and led to an increase in the yield (Table 1, entry 18). Notably, the different reaction conditions had no obvious influence on the diastereoselectivity at the C_5_- and C_6_- positions and produced the product **3aa** with *trans* configuration. Interestingly, atropisomerism caused by the high rotational barrier around the aryl-*N* bond in the benzofuran-fused eight-membered lactam **3aa** was observed. The atropisomers were systematically obtained as an approximate 5:1 mixture regardless of the reaction conditions [72]. The structure of **3aa** was unambiguously determined with X-ray diffraction analysis.

**Scheme 2:**
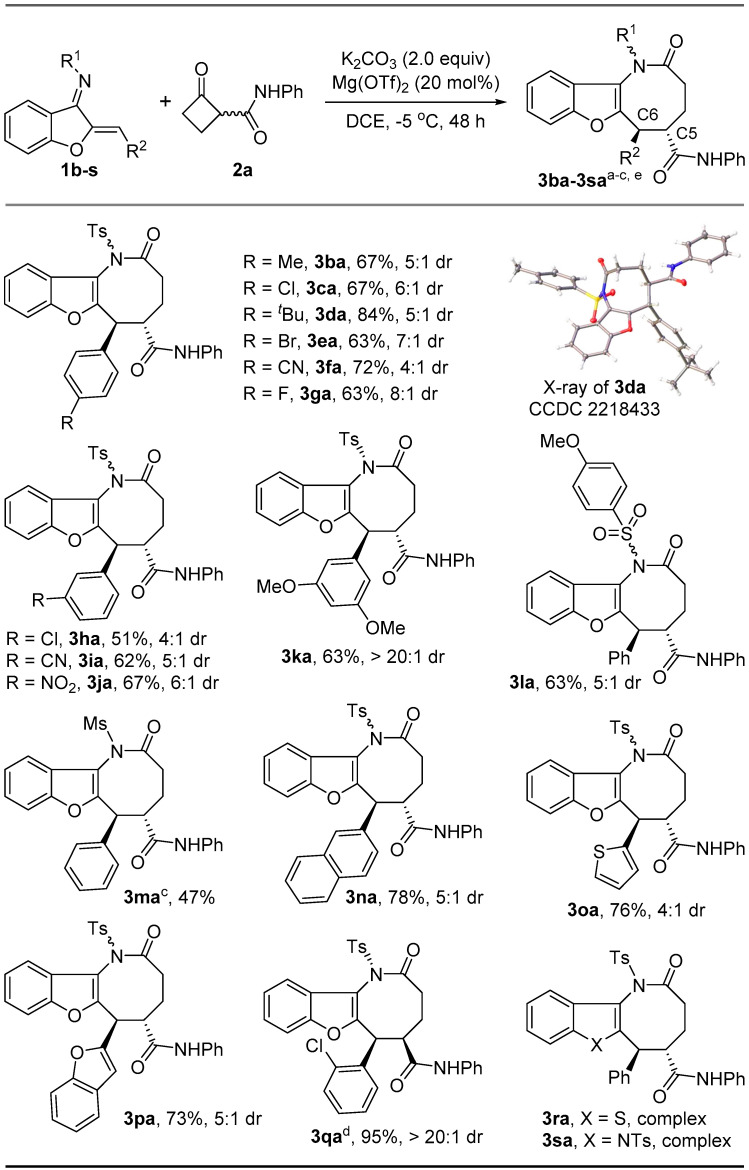
Scope of azadienes. ^a^ dr was determined with ^1^H NMR analysis. ^b^ **3aa**–**3la, 3na**–**3pa** were obtained as atropoisomer mixture, only C_5_−C_6_ *trans* product was observed. ^c^ No atropisomers. ^d^ Only C_5_−C_6_ *cis* product was observed. ^e^ C_5_−C_6_ *cis* and *trans* diastereoisomers are separable using flash columns on silica gel.

With the optimal reaction conditions established, we sought to explore the substrate scope of the reaction. As indicated in Scheme 2, *N*-tosyl azadienes bearing electron donating or withdrawing groups at the para-position of the aryl rings were compatible with the cascade reaction, thus delivering the corresponding eight-membered lactam in a moderate to high yield (**3ba-3ga**) with 4:1 to 8:1 diastereoselectivities with regard to the axial chirality. Switching the substituents from the para- to the meta-position was also feasible (**3ha-3ja**). In addition, azadiene with a 3,5-dimethoxyl group in the aryl ring was also well-tolerated, giving a product with a high yield and diastereoselectivity (**3ka**). Moreover, replacement of the *N*-tosyl with a 4-methoxy-benzene sulfonyl or mesyl group led to slightly reduced yields (**3la**, **3ma**), and the atropisomerism disappeared in the product **3ma**. Furthermore, when the phenyl ring was changed to 2-naphthyl, 2-thienyl or 2-benzofuranyl in the azadienes, the corresponding products were generated with a good yield and moderate diastereoselectivity (**3na-3pa**). Interestingly, *N*-tosyl azadiene bearing chloro at the ortho-position of the aryl rings gave the product **3qa** as a *cis* diastereomer at the C_5_ and C_6_ positions, and only one atropisomer was obtained in this case. Moreover, benzothiophene- or indole-fused azadienes are not appropriate substrates for this reaction under the optimized conditions (**3ra, 3sa**). The relative configuration of **3aa** and **3da** was unambiguously confirmed with single-crystal X-ray diffraction analysis, and the other products were assigned by analogy (see Supplementary Materials for the assignment of diastereoselectivity at the C_5_ and C_6_ positions).

**Scheme 3:**
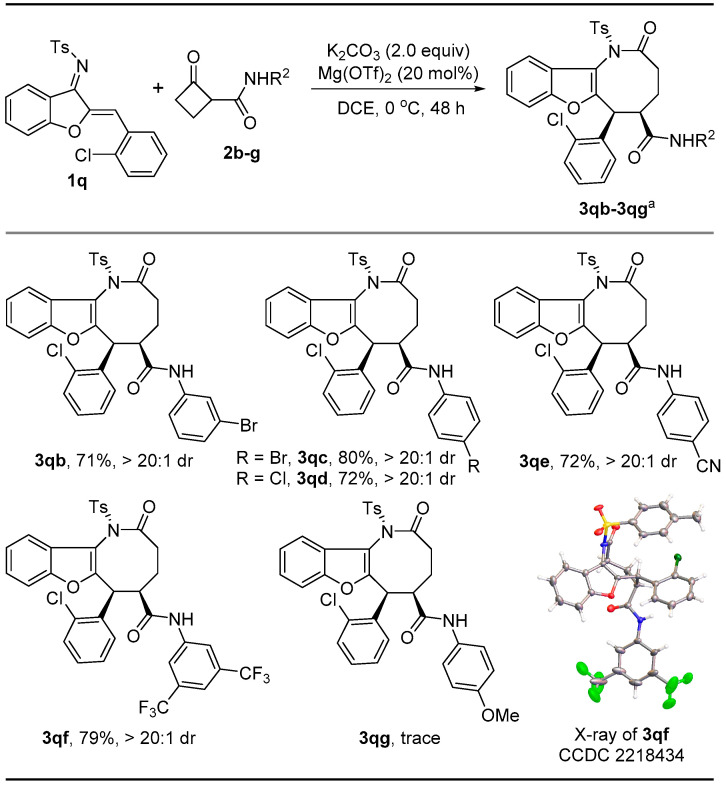
Scope of cyclobutanones. ^a^ Only C_5_-C_6_ *cis* product was observed and only one atropisomer.

Subsequently, an investigation of the scope of the cyclobutanone was conducted using **1q** as a model azadiene (Scheme 3). The electronic and steric character of the *N*-aryl amides of the cyclobutanone were evaluated by varying various substituents at different positions. Cyclobutanone substrates bearing a bromo group at the meta- or para-position on the *N*-aryl amides were tolerated in this reaction, affording the products as *cis* diastereomers at the C_5_- and C_6_- positions with good yields and high diastereoselectivities (**3qb**, **3qc**). Incorporating a chloro or CN substituent at the para-position of the *N*-aryl amides led to the desired eight-membered lactam with a good yield and high diastereoselectivity (**3qd**, **3qe**). A strong electronic-withdrawing CF_3_ group at the 3,5-positions of the *N*-aryl amide was also applicable, delivering the product **3qf** in 79% yield. However, an electronic-donating OMe group at the 4-positions of the *N*-aryl amide was not applicable (**3qg**). The relative configuration of **3qf** was unambiguously confirmed using single-crystal X-ray diffraction analysis, and the other products were assigned by analogy.

Furthermore, the synthetic potential of this strategy was demonstrated with a gram-scale reaction, the annulation proceeded smoothly under the optimal conditions and the adduct **3aa** was obtained in 61% yield.

**Scheme 4:**
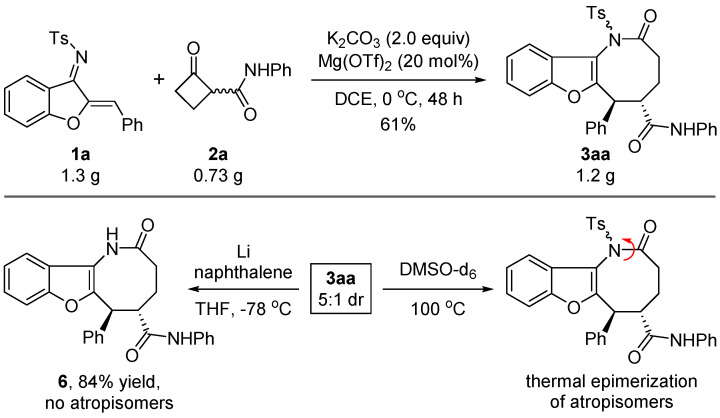
Scale-up synthesis of **3aa**, thermal epimerization and loss of atropisomerism study.

In addition, the atropisomerism in the eight-membered lactam **3aa** disappeared by removing the *N*-tosyl group to afford **6**. Moreover, high-temperature proton NMR experiments of **3aa** in DMSO-*d_6_* were performed, suggesting an isomeric interconversion of the atropisomers at 100 °C (Scheme 4 and Supplementary Materials).

**Scheme 5:**
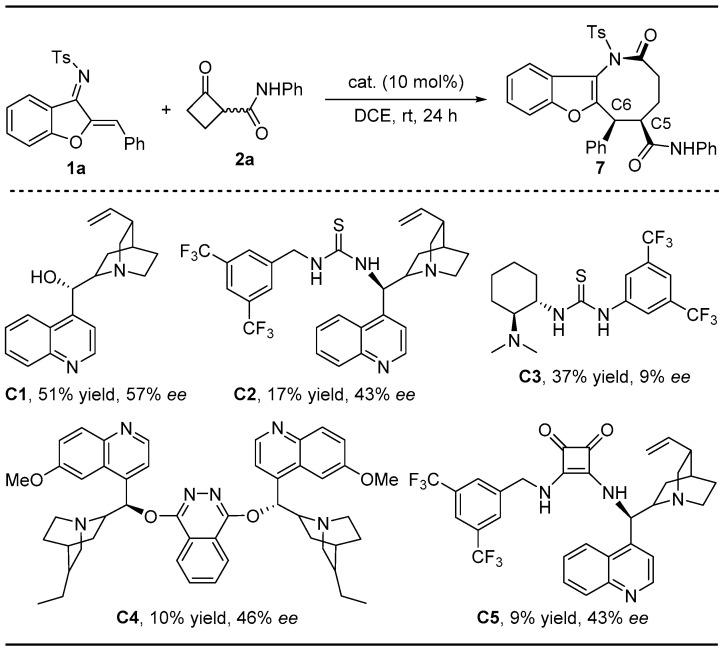
Attempts for the catalytic asymmetric Michael addition/ring-expansion cascade.

The catalytic asymmetric version of the Michael addition/ring-expansion cascade has also been explored using a chiral base such as a cinchona alkaloid, quinine-derived bifunctional thiourea catalyst, Takemoto catalyst and dimeric quinidine derivatives, as well as quinine-derived squaramide catalyst. As indicated in Scheme 5, the product **3aa** was obtained as *cis* diastereomers at the C_5_- and C_6_- positions when chiral bases were used as catalysts. Although the yields and the enantioselectivities are not synthetically practical at the current stage (cat. **C1**, up to 51% yield, 56% ee), these results demonstrate that this protocol might provide opportunities for stereoselective library collections.

## 3. Materials and Methods

All reactions in non-aqueous media were conducted under a positive pressure of dry argon in glassware that had been dried in an oven prior to use unless noted otherwise. Anhydrous solutions of reaction mixtures were transferred via an oven-dried syringe or cannula. Chemicals were purchased from commercial sources; dichloromethane (DCM), *n*-hexane, ethyl acetate (EA), methanol (MeOH), tetrahydrofuran (THF), acetone and petroleum ether (PE) were purchased from Beijing Chemical Factory (Beijing, China). Silica gel for analytical thin-layer chromatography (TLC) and column chromatography (200~300 mesh) was purchased from Qingdao Haiyang Chemical Co., Ltd. (Qingdao, China) & Special Silica Gel Factory (Taiyuan, China). ^1^H and ^13^C nuclear magnetic resonance spectra (NMR) were obtained on a JEOL Delta (400 MHz and 600 MHz) and recorded in ppm (δ) downfield of TMS (δ = 0) in CDCl_3_, DMSO-*d*_6_, unless noted otherwise. Signal-splitting patterns were described as singlet (s), doublet (d), triplet (t), quartet (q), quintet (quint) or multiplet (m), with coupling constants (*J*) in hertz. HPLC analysis was conducted on a SHIMADZU LC-20ADXR instrument with chiral columns (Chiralpak IF, column 4.6 × 250 mm, (Daicel Chemical Ind., Ltd., Tokyo, Japan)). High-resolution mass spectra (HRMS) were recorded on a Waters LCT Premier XE mass spectrometer with TOF. Crystallographic data were collected using a Rigaku Oxford Diffraction XtaLAB Synergy diffractometer (Tokyo, Japan) equipped with a HyPix-6000E area detector at 173 K using Cu Kα (λ = 1.54184 Å) from a PhotonJet micro-focus X-ray source.

### 3.1. General Procedure for the Synthesis of **3**

To an oven-dried flask was added **1** (0.1 mmol), **2** (0.11 mmol), K_2_CO_3_ (27.6 mg, 0.2 mmol), Mg(OTf)_2_ (6.4 mg, 0.02 mmol) and dry DCE (1.5 mL) under Ar. The reaction mixture was stirred at −5 °C and monitored with TLC. After completion (~48 h), the reaction mixture was purified with flash column chromatography to yield the product.

**3aa**, 78%, white solid. ^1^H NMR (600 MHz, CDCl_3_) δ 8.26 (d, *J* = 7.8 Hz, 2H), 7.50–7.38 (m, 5H), 7.36–7.27 (m, 3H), 7.22–7.13 (m, 5H), 7.05–6.96 (m, 3H), 6.59 (s, 1H), 4.57 (d, *J* = 11.8 Hz, 1H), 3.22 (t, *J* = 12.0 Hz, 1H), 2.83–2.67 (m, 1H), 2.45 (s, 3H), 2.39–2.17 (m, 3H). ^13^C NMR (101 MHz, CDCl_3_) δ 173.2, 170.4, 153.5, 146.1, 138.9, 136.6, 135.9, 130.2, 129.6, 129.1, 128.9, 128.7, 127.9, 126.1, 125.6, 125.1, 123.8, 120.9, 120.6, 119.3, 116.3, 111.9, 48.9, 47.9, 33.4, 29.4, 21.9. HRMS (ESI) was calculated for C_33_H_29_N_2_O_5_S [M + H]^+^ 565.1792, found 565.1893.

**3ba**, 67%, white solid. ^1^H NMR (600 MHz, CDCl_3_) δ 8.30 (d, *J* = 8.3 Hz, 2H), 7.48–7.46 (m, 3H), 7.38 (d, *J* = 8.0 Hz, 3H), 7.33–7.28 (m, 2H), 7.23 (t, *J* = 7.7 Hz, 2H), 7.11–7.08 (m, 5H), 6.71 (s, 1H), 4.58 (d, *J* = 11.8 Hz, 1H), 3.28–3.19 (m, 1H), 2.83–2.73 (m, 1H), 2.51 (s, 3H), 2.41–2.27 (m, 3H), 2.25 (s, 3H). ^13^C NMR (151 MHz, CDCl_3_) δ 172.6, 169.8, 155.1, 152.8, 145.4, 136.9, 136.2, 135.1, 129.5, 129.0, 128.9, 128.1, 127.8, 125.3, 124.7, 124.1, 123.0, 120.0, 118.5, 115.4, 111.2, 47.8, 47.1, 32.8, 28.7, 21.2, 20.4. HRMS (ESI) was calculated for C_34_H_21_N_2_O_5_S [M + H]^+^ 579.1948, found 579.1957.

**3ca**, 67%, white solid. ^1^H NMR (600 MHz, CDCl_3_) δ 8.27 (d, *J* = 8.0 Hz, 2H), 7.47 (dd, *J* = 13.1, 8.6 Hz, 4H), 7.41–7.38 (m, 2H), 7.35–7.26 (m, 4H), 7.23 (s, 1H), 7.19–7.10 (m, 4H), 7.07 (t, *J* = 7.7 Hz, 1H), 4.63 (d, *J* = 11.9 Hz, 1H), 3.32 (t, *J* = 11.5 Hz, 1H), 2.77–2.72 (m, 1H), 2.52 (s, 3H), 2.40–2.26 (m, 3H). ^13^C NMR (151 MHz, CDCl_3_) δ 173.1, 170.1, 155.1, 153.5, 146.2, 137.3, 136.7, 135.7, 133.8, 130.1, 129.6, 129.1, 128.9, 125.9, 125.7, 125.0, 123.8, 120.7, 119.2, 116.3, 111.9, 108.2, 48.0, 47.7, 33.4, 29.4, 21.8. HRMS (ESI) was calculated for C_33_H_28_ClN_2_O_5_S [M + H]^+^ 599.1402, found 599.1410.

**3da**, 84%, white solid. ^1^H NMR (600 MHz, CDCl_3_) δ 8.29 (d, *J* = 7.7 Hz, 2H), 7.47 (d, *J* = 7.8 Hz, 2H), 7.44 (d, *J* = 7.2 Hz, 1H), 7.41–7.31 (m, 3H), 7.32–7.28 (m, 4H), 7.18 (t, *J* = 7.8 Hz, 2H), 7.04 (t, *J* = 7.5 Hz, 1H), 6.96 (d, *J* = 7.8 Hz, 2H), 6.66 (s, 1H), 4.55 (d, *J* = 11.8 Hz, 1H), 3.26 (t, *J* = 11.9 Hz, 1H), 2.79–2.74 (m, 1H), 2.49 (s, 3H), 2.41–2.28 (m, 3H), 1.21 (s, 9H). ^13^C NMR (101 MHz, CDCl_3_) δ 173.4, 170.6, 155.8, 153.6, 150.8, 146.13, 136.7, 136.0, 136.0, 130.3, 129.7, 128.8, 128.2, 126.2, 126.2, 125.5, 125.1, 123.8, 121.2, 119.3, 116.2, 112.0, 48.8, 47.9, 34.6, 33.5, 31.3, 29.2, 21.9. HRMS (ESI) was calculated for C_37_H_37_N_2_O_5_S [M + H]^+^ 621.2418, found 621.2410.

**3ea**, 63%, white solid. ^1^H NMR (600 MHz, CDCl_3_) δ 8.28 (d, *J* = 6.6 Hz, 2H), 7.51–7.48 (m, 2H), 7.43 (m, 4H), 7.40 (m, 2H), 7.36–7.28 (m, 2H), 7.27 (d, *J* = 1.8 Hz, 1H), 7.24 (d, *J* = 1.7 Hz, 1H), 7.17–7.13 (m, 2H), 7.12–7.07 (m, 1H), 6.98 (s, 1H), 4.63 (d, *J* = 12.3 Hz, 1H), 3.38–3.26 (m, 1H), 2.87–2.68 (m, 1H), 2.52 (s, 3H), 2.35 (m, 3H). ^13^C NMR (151 MHz, CDCl_3_) δ 173.2, 170.2, 155.1, 153.5, 146.3, 137.9, 136.7, 135.8, 132.2, 130.5, 130.2, 129.7, 125.9, 125.7, 125.1, 123.9, 122.1, 120.8, 119.3, 116.3, 112.0, 48.1, 47.6, 33.5, 29.5, 21.9. HRMS (ESI) was calculated for C_33_H_28_BrN_2_O_5_S [M + H]^+^ 643.0879, found 643.0886.

**3fa**, 63%, white solid. ^1^H NMR (400 MHz, CDCl_3_) δ 8.26 (d, *J* = 8.0 Hz, 2H), 7.72–7.56 (m, 4H), 7.48 (d, *J* = 8.1 Hz, 2H), 7.42–7.29 (m, 6H), 7.22–7.15 (m, 3H), 7.11–7.01 (m, 1H), 4.74 (d, *J* = 12.0 Hz, 1H), 3.50–3.43 (m, 1H), 2.82–2.64 (m, 1H), 2.52 (s, 3H), 2.40–2.28 (m, 2H), 2.08–2.05 (m, 1H). ^13^C NMR (151 MHz, CDCl_3_) δ 173.2, 167.0, 154.7, 153.7, 146.5, 144.2, 136.9, 132.8, 132.1, 130.1, 129.9, 129.8, 129.1, 126.0, 125.7, 125.2, 124.1, 120.5, 119.3, 116.6, 112.1, 111.8, 48.5, 47.2, 33.6, 29.6, 22.0. HRMS (ESI) was calculated for C_34_H_28_N_3_O_5_S [M + H]^+^ 590.1744, found 590.1751.

**3ga**, 84%, white solid. ^1^H NMR (600 MHz, CDCl_3_) δ 8.24 (d, *J* = 8.2 Hz, 2H), 7.55–7.40 (m, 4H), 7.35–7.31 (m, 3H), 7.28–7.27 (m, 1H), 7.24–7.22 (m, 2H), 7.22–7.20 (m, 3H), 7.10 (d, *J* = 7.6 Hz, 1H), 7.05–7.01 (m, 1H), 6.78 (s, 1H), 4.81 (d, *J* = 11.8 Hz, 1H), 3.20 (td, *J* = 11.5, 3.0 Hz, 1H), 2.78–2.68 (m, 1H), 2.50 (s, 3H), 2.38–2.24 (m, 3H). ^13^C NMR (101 MHz, CDCl_3_) δ 173.2, 170.3, 155.5, 153.5, 146.2, 136.7, 135.7, 134.7, 134.6, 130.4, 130.4, 130.1, 129.6, 128.9, 125.9, 125.6, 125.1, 123.8, 120.6, 119.2, 116.1, 116.0, 115.8, 111.9, 47.9, 47.8, 33.4, 29.3, 21.8. HRMS (ESI) was calculated for C_34_H_28_FN_2_O_5_S [M + H]^+^ 583.1697, found 583.1690.

**3ha**, 51%, white solid. ^1^H NMR (400 MHz, CDCl_3_) δ 8.29 (d, *J* = 8.0 Hz, 2H), 7.54 (s, 1H), 7.49–7.44 (m, 3H), 7.41–7.39 (m, 2H), 7.35–7.28 (m, 2H), 7.23–7.16 (m, 4H), 7.12 (d, *J* = 8.0 Hz, 2H), 7.07–7.04 (m, 1H), 6.82 (s, 1H), 4.60 (d, *J* = 11.8 Hz, 1H), 3.22 (t, *J* = 11.1 Hz, 1H), 2.86–2.67 (m, 1H), 2.50 (s, 3H), 2.40–2.25 (m, 3H). ^13^C NMR (151 MHz, CDCl_3_) δ 173.0, 169.9, 154.6, 153.4, 146.1, 140.6, 136.5, 134.7, 130.2, 130.1, 130.0, 129.5, 129.3, 128.8, 128.7, 128.0, 126.8, 125.8, 125.6, 125.0, 123.8, 120.4, 119.3, 111.8, 48.2, 47.5, 33.3, 29.3, 21.8. HRMS (ESI) was calculated for C_32_H_28_ClN_2_O_5_S [M + H]^+^ 599.1402, found 599.1410.

**3ia**, 62%, white solid. ^1^H NMR (400 MHz, CDCl_3_) δ 8.28 (d, *J* = 7.6 Hz, 2H), 7.88 (s, 1H), 7.82 (d, *J* = 8.9 Hz, 1H), 7.54–7.48 (m, 3H), 7.44–7.33 (m, 7H), 7.20–7.15 (m, 2H), 7.10–7.05 (m, 2H), 4.72 (d, *J* = 11.9 Hz, 1H), 3.46–3.37 (m, 1H), 2.87–2.71 (m, 1H), 2.53 (s, 3H), 2.43–2.33 (m, 3H). ^13^C NMR (101 MHz, CDCl_3_) δ 173.1, 169.9, 154.5, 153.7, 146.4, 140.5, 136.7, 135.8, 133.2, 132.8, 131.7, 130.1, 129.9, 129.1, 126.0, 125.8, 125.3, 124.1, 120.6, 120.3, 119.4, 118.5, 116.7, 113.1, 112.1, 48.2, 47.41, 33.5, 29.6, 22.0. HRMS (ESI) was calculated for C_34_H_28_N_3_O_5_S [M + H]^+^ 590.1744, found 590.1750.

**3ja**, 51%, white solid. ^1^H NMR (400 MHz, CDCl_3_) δ 8.28 (d, *J* = 8.6 Hz, 2H), 8.16 (d, *J* = 8.9 Hz, 2H), 7.76 (d, *J* = 8.8 Hz, 2H), 7.50 (d, *J* = 8.3 Hz, 2H), 7.46–7.32 (m, 5H), 7.24–7.15 (m, 4H), 7.12–7.01 (m, 1H), 4.82 (d, *J* = 11.9 Hz, 1H), 3.62–3.42 (m, 1H), 2.86–2.68 (m, 1H), 2.54 (s, 3H), 2.42–2.34 (m, 2H), 1.99 (d, *J* = 5.2 Hz, 1H). ^13^C NMR (151 MHz, CDCl_3_) δ 172.8, 169.7, 154.2, 153.5, 147.3, 146.3, 145.9, 136.5, 135.5, 129.9, 129.8, 129.7, 128.9, 125.9, 125.5, 125.1, 124.0, 123.9, 120.3, 119.1, 116.5, 111.9, 48.1, 47.1, 33.3, 29.6, 21.8. HRMS (ESI) was calculated for C_32_H_28_N_3_O_7_S [M + H]^+^ 610.1642, found 610.1637.

**3ka**, 63%, white solid. ^1^H NMR (600 MHz, CDCl_3_) δ 8.30 (d, *J* = 8.1 Hz, 2H), 7.49 (d, *J* = 8.2 Hz, 2H), 7.45–7.41 (m, 2H), 7.34 (td, *J* = 8.2, 7.7, 1.5 Hz, 1H), 7.32–7.28 (m, 1H), 7.24 (t, *J* = 7.9 Hz, 2H), 7.20–7.16 (m, 2H), 7.10–7.05 (m, 2H), 6.70 (d, *J* = 2.3 Hz, 2H), 6.29 (t, *J* = 2.3 Hz, 1H), 4.57 (d, *J* = 12.0 Hz, 1H), 3.69 (s, 6H), 3.35 (td, *J* = 11.6, 2.8 Hz, 1H), 2.80–2.70 (m, 1H), 2.52 (s, 3H), 2.42–2.29 (m, 3H). ^13^C NMR (101 MHz, CDCl_3_) δ 173.4, 170.6, 161.3, 155.8, 153.6, 146.2, 141.1, 137.1, 135.9, 130.3, 130.1, 129.7, 129.5, 128.9, 126.0, 125.6, 124.9, 123.9, 120.9, 119.1, 116.3, 112.1, 106.3, 101.0, 55.8, 49.2, 47.9, 33.5, 29.1, 22.0. HRMS (ESI) was calculated for C_35_H_33_N_2_O_7_S [M + H]^+^ 625.2003, found 625.2009.

**3la**, 63%, white solid. ^1^H NMR (400 MHz, CDCl_3_) δ 8.35 (d, *J* = 9.0 Hz, 2H), 7.52–7.44 (m, 3H), 7.40–7.36 (m, 1H), 7.33–7.27 (m, 4H), 7.22–7.18 (m, 3H), 7.13–7.04 (m, 5H), 6.67 (s, 1H), 4.61 (d, *J* = 11.9 Hz, 1H), 3.91 (s, 3H), 3.23 (td, *J* = 11.8, 2.8 Hz, 1H), 2.85–2.73 (m, 1H), 2.46–2.23 (m, 3H). ^13^C NMR (101 MHz, CDCl_3_) δ 173.3, 170.5, 164.7, 155.5, 153.5, 139.0, 136.9, 132.7, 130.0, 129.1, 129.0, 128.7, 128.0, 126.2, 125.6, 125.0, 123.8, 120.8, 119.3, 116.5, 114.2, 112.0, 56.0, 48.9, 47.9, 33.5, 29.5. HRMS (ESI) was calculated for C_33_H_29_N_2_O_6_S [M + H]^+^ 581.1741, found 581.1747.

**3ma**, 47%, white solid. ^1^H NMR (400 MHz, CDCl_3_) δ 7.44–7.36 (m, 4H), 7.35–7.27 (m, 4H), 7.22–7.15 (m, 3H), 7.12–7.02 (m, 3H), 6.98 (s, 1H), 4.63 (d, *J* = 12.0 Hz, 1H), 3.84 (s, 3H), 3.41 (td, *J* = 11.5, 3.7 Hz, 1H), 2.90 (td, *J* = 11.7, 8.2 Hz, 1H) and 2.56–2.36 (m, 3H). ^13^C NMR (151 MHz, CDCl_3_) δ 174.8, 170.4, 156.1, 153.5, 138.9, 136.8, 129.2,128.8, 128.5, 127.9 125.6, 125.6, 124.9, 123.8, 120.9, 118.7, 114.9, 112.1, 48.9, 47.9, 44.5, 33.3 and 29.1. HRMS (ESI) was calculated for C_27_H_25_N_2_O_5_S [M + H]^+^ 489.1479, found 489.1486.

**3na**, 78%, white solid. ^1^H NMR (400 MHz, CDCl_3_) δ 8.33 (d, *J* = 7.2 Hz, 2H), 8.01 (s, 1H), 7.83–7.71 (m, 3H), 7.67–7.60 (m, 1H), 7.51–7.48 (m, 1H), 7.45 (d, *J* = 7.9 Hz, 2H), 7.42–7.37 (m, 2H), 7.36–7.34 (m, 1H), 7.32–7.29 (m, 2H), 7.17–7.07 (m, 2H), 7.00–6.86 (m, 3H), 6.63 (s, 1H), 4.80 (d, *J* = 12.0 Hz, 1H), 3.34 (t, *J* = 11.7 Hz, 1H), 2.89–2.74 (m, 1H), 2.48 (s, 3H), 2.46–2.29 (m, 3H). ^13^C NMR (101 MHz, CDCl_3_) δ 173.3, 170.5, 155.6, 153.6, 146.2, 136.6, 136.4, 136.0, 133.6, 132.9, 130.3, 129.7, 128.9, 128.8, 128.3, 128.0, 127.7, 126.4, 126.3, 125.7, 125.0, 123.9, 120.9, 119.4, 116.6, 112.0, 47.0, 48.1, 33.6, 29.5, 21.9. HRMS (ESI) was calculated for C_37_H_31_N_2_O_5_S [M + H]^+^ 615.1948, found 615.1957.

**3oa**, 76%, white solid. ^1^H NMR (600 MHz, CDCl_3_) δ 8.25 (d, *J* = 8.2 Hz, 2H), 7.50–7.42 (m, 4H), 7.39–7.30 (m, 3H), 7.28–7.26 (m, 1H), 7.25–7.24 (m, 1H), 7.22–7.20 (m, 3H), 7.12–7.01 (m, 2H), 6.79 (s, 1H), 4.82 (d, *J* = 11.8 Hz, 1H), 3.21 (td, *J* = 11.5, 3.0 Hz, 1H), 2.78–2.69 (m, 1H), 2.50 (s, 3H), 2.38–2.24 (m, 3H). ^13^C NMR (151 MHz, CDCl_3_) δ 173.2, 170.8, 155.5, 153.5, 146.1, 139.3, 137.0, 130.2, 129.6, 129.0, 127.7, 126.5, 125.6, 125.0, 123.9, 120.8, 119.3, 115.8, 112.0, 48.0, 43.8, 33.5, 29.0, 21.9. HRMS (ESI) was calculated for C_31_H_27_N_2_O_5_S_2_ [M + H]^+^ 571.1356, found 571.1350.

**3pa**, 73%, white solid. ^1^H NMR (400 MHz, CDCl_3_) δ 8.36 (d, *J* = 8.3 Hz, 2H), 7.70–7.64 (m, 1H), 7.49–7.33 (m, 8H), 7.23–7.13 (m, 4H), 7.13–7.06 (m, 1H), 7.06–7.00 (m, 2H), 6.71 (s, 1H), 4.89 (d, *J* = 11.8 Hz, 1H), 2.98 (td, *J* = 11.8, 3.2 Hz, 1H), 2.72 (td, *J* = 12.0, 8.2 Hz, 1H), 2.43 (s, 3H), 2.39–2.17 (m, 3H). ^13^C NMR (101 MHz, CDCl_3_) δ 172.7, 170.1, 155.0, 153.6, 153.5, 151.2, 146.1, 136.8, 135.8, 130.6, 129.4, 129.0, 128.2, 126.4, 126.0, 125.1, 124.7, 124.2, 123.3, 121.5, 120.6, 120.2, 117.5, 111.9, 111.0, 106.0, 45.5, 42.6, 33.3, 28.9, 21.9. HRMS (ESI) was calculated for C_31_H_27_N_2_O_5_S_2_ [M + H]^+^ 571.1356, found 571.1350.

**3qa**, 95%, white solid. ^1^H NMR (400 MHz, CDCl_3_) δ 8.33 (d, *J* = 5.1 Hz, 1H), 7.84 (d, *J* = 6.7 Hz, 1H), 7.70 (d, *J* = 7.9 Hz, 2H), 7.46–7.26 (m, 10H), 7.13 (t, *J* = 7.3 Hz, 1H), 6.94 (d, *J* = 8.0 Hz, 2H), 6.57 (s, 1H), 3.82 (s, 1H), 2.85 (t, *J* = 12.6 Hz, 1H), 2.77 (d, *J* = 5.5 Hz, 1H), 2.55 (dd, *J* = 12.8, 7.7 Hz, 1H), 2.33 (s, 3H), 2.25 (dd, *J* = 14.8, 7.7 Hz, 1H), 2.03 (td, *J* = 14.6, 13.8, 5.5 Hz, 1H). ^13^C NMR (101 MHz, CDCl_3_) δ 174.4, 169.7, 154.8, 150.5, 145.5, 137.5, 136.2, 134.6, 133.8, 130.3, 130.0, 129.6, 129.5, 129.2, 128.9, 127.5, 126.1, 125.0, 124.6, 124.4, 121.2, 119.8, 119.4, 111.8, 46.7, 39.9, 33.1, 28.4, 21.8. HRMS (ESI) was calculated for C_32_H_28_ClN_2_O_5_S [M + H]^+^ 599.1402, found 599.1409.

**3qb**, 71%, white solid. ^1^H NMR (600 MHz, CDCl_3_) δ 8.33–8.28 (m, 1H), 7.88–7.84 (m, 1H), 7.70 (d, *J* = 8.2 Hz, 2H), 7.57 (s, 1H), 7.46–7.33 (m, 6H), 7.30–7.27 (m, 1H), 7.17 (t, *J* = 8.0 Hz, 1H), 7.10 (dt, *J* = 8.5, 1.2 Hz, 1H), 6.94 (d, *J* = 8.1 Hz, 2H), 6.56 (s, 1H), 3.80 (d, *J* = 1.4 Hz, 1H), 2.85–2.79 (m, 1H), 2.78 (d, *J* = 5.8 Hz, 1H), 2.56 (ddd, *J* = 13.1, 7.9, 1.8 Hz, 1H), 2.33 (s, 3H), 2.24 (ddt, *J* = 14.8, 8.0, 1.8 Hz, 1H), 2.05 (dddd, *J* = 14.4, 12.7, 5.8, 1.8 Hz, 1H). ^13^C NMR (151 MHz, CDCl_3_) δ 173.5, 169.2, 154.0, 149.5, 144.8, 138.0, 135.3, 133.9, 132.9, 129.7, 129.1, 128.9, 128.7, 128.1, 126.8, 126.8, 125.5, 124.2, 123.7, 122.1, 120.5, 118.8, 117.4, 111.0, 46.0, 39.1, 32.3, 27.5, 21.03. HRMS (ESI) was calculated for C_33_H_27_BrClN_2_O_5_S [M + H]^+^ 677.0507, found 677.0517.

**3qc**, 80%, white solid. ^1^H NMR (400 MHz, CDCl_3_) δ 8.32–8.25 (m, 1H), 7.89–7.81 (m, 1H), 7.69 (d, *J* = 8.1 Hz, 2H), 7.46–7.36 (m, 7H), 7.32–7.27 (m, 1H), 7.19–7.13 (m, 2H), 6.93 (d, *J* = 8.2 Hz, 2H), 6.59 (s, 1H), 3.80 (s, 1H), 2.86–2.74 (m, 2H), 2.62–2.50 (m, 1H), 2.33 (s, 3H), 2.24 (dd, *J* = 14.7, 7.9 Hz, 1H), 2.03 (td, *J* = 13.0, 5.6 Hz, 1H). ^13^C NMR (101 MHz, CDCl_3_) δ 174.3, 169.8, 154.7, 136.6, 136.0, 134.6, 133.7, 132.2, 130.4, 129.9, 129.7, 129.5, 128.9, 127.5, 126.2, 124.9, 124.5, 121.3, 121.2, 119.5, 117.1, 111.7, 46.7, 39.8, 33.1, 28.3, 21.8. HRMS (ESI) was calculated for C_33_H_27_BrClN_2_O_5_S [M + H]^+^ 677.0507, found 677.0516.

**3qd**, 72%, white solid. ^1^H NMR (600 MHz, CDCl_3_) δ 8.34–8.27 (m,1H), 7.88–7.81 (m, 1H), 7.69 (d, *J* = 8.3 Hz, 2H), 7.45–7.35 (m, 5H), 7.32–7.26 (m, 3H), 7.22 (d, *J* = 8.8 Hz, 2H), 6.94 (d, *J* = 8.0 Hz, 2H), 6.60 (s, 1H), 3.80 (s, 1H), 2.82 (t, *J* = 12.3 Hz, 1H), 2.77 (d, *J* = 5.6 Hz, 1H), 2.55 (ddd, *J* = 13.1, 7.9, 1.5 Hz, 1H), 2.33 (s, 3H), 2.24 (dd, *J* = 14.7, 8.0 Hz, 1H), 2.03 (dddd, *J* = 14.4, 12.4, 5.6, 1.7 Hz, 1H). ^13^C NMR (151 MHz, CDCl_3_) δ 174.3, 169.8, 154.7, 150.4, 145.6, 136.1, 136.1, 134.7, 133.7, 130.5, 129.9, 129.7, 129.5, 129.5, 129.2, 128.9, 127.5, 126.2, 125.0, 124.5, 121.3, 120.9, 119.5, 111.7, 46.7, 39.9, 33.1, 28.3, 21.8. HRMS (ESI) was calculated for C_33_H_27_Cl_2_N_2_O_5_S [M + H]^+^ 633.1012, found 633.1017.

**3qe**, 72%, white solid. ^1^H NMR (400 MHz, CDCl_3_) δ 8.25–8,23 (m, 1H), 7.88–7.78 (m, 1H), 7.67 (d, *J* = 7.9 Hz, 2H), 7.58 (d, *J* = 8.3 Hz, 2H), 7.46–7.33 (m, 7H), 7.23–7.15 (m, 1H), 6.92 (d, *J* = 8.0 Hz, 2H), 6.86 (s, 1H), 3.79 (s, 1H), 2.82 (d, *J* = 5.3 Hz, 1H), 2.77 (t, *J* = 12.6 Hz, 1H), 2.54 (dd, *J* = 13.0, 7.7 Hz, 1H), 2.32 (s, 3H), 2.23 (dd, *J* = 14.7, 7.7 Hz, 1H), 2.04 (td, *J* = 16.3, 14.6, 5.6 Hz, 1H). ^13^C NMR (101 MHz, CDCl_3_) δ 174.1, 170.4, 154.7, 150.1, 145.6, 141.5, 135.8, 134.7, 133.6, 133.5, 130.5, 129.8, 129.7, 129.5, 128.9, 127.5, 126.3, 124.9, 124.6, 121.3, 119.6, 119.4, 118.8, 111.5, 107.4, 46.9, 39.8, 33.0, 28.2, 21.8. HRMS (ESI) was calculated for C_34_H_27_ClN_3_O_5_S [M + H]^+^ 624.1354, found 624.1354.

**3qf**, 79%, white solid. ^1^H NMR (400 MHz, CDCl_3_) δ 8.35–8.17 (m, 1H), 7.92–7.83 (m, 1H), 7.76 (s, 2H), 7.72–7.63 (m,3H), 7.50–7.34 (m, 5H), 7.24 (d, *J* = 5.6 Hz, 1H), 6.93 (d, *J* = 8.0 Hz, 2H), 6.88 (s, 1H), 3.80 (s, 1H), 2.89–2.73 (m, 2H), 2.57 (dd, *J* = 13.0, 7.7 Hz, 1H), 2.33 (s, 3H), 2.25 (dd, *J* = 14.7, 7.7 Hz, 1H), 2.08 (td, *J* = 14.1, 13.3, 5.3 Hz, 1H).^13^C NMR (101 MHz, CDCl_3_) δ 174.1, 170.5, 154.7, 150.0, 145.6, 138.8, 135.8, 134.7, 131.6 (q, *J* = 33.3 Hz, 2C), 129.9, 129.6, 129.4, 128.8, 127.5, 126.3, 124.6, 123.1 (q, *J* = 273.7 Hz, 2C), 121.30, 119.40, 117.8, 111.5, 46.8, 39.8, 33.0, 28.0, 21.8. HRMS (ESI) was calculated for C_35_H_26_ClF_6_N_3_O_5_S [M + H]^+^ 735.1150, found 735.1157.

### 3.2. General Procedure for the Synthesis of **6**

Naphthalene (448 mg, 3.5 mmol, 1.0 equiv) was dissolved in THF (7 mL) under N_2_. Lithium (25 mg, 3.5 mmol, 1.0 equiv) was added and the mixture stirred for 2 h at room temperature. A dark green Li/Naphthalene solution (0.5 M) was obtained. To an oven-dried flask was added **3aa** (56 mg, 0.1 mmol, 1.0 equiv) and dry THF (1.5 mL) under Ar at −78 °C. Li/Naphthalene solution (0.5 M in THF) was added dropwise until the dark green solution turned colorless, then the reaction mixture was stirred at RT for 30 min. Quenched with 1 M NaHCO_3_ (5 mL), the aqueous layer was extracted with EtOAc (3 × 10 mL), and the combined organic layer was dried with Na_2_SO_4_, filtered and concentrated in vacuo. Purification with flash column chromatography yielded the product **6** (84% yield) as a white solid. ^1^H NMR (400 MHz, DMSO-*d*_6_) δ 9.87 (s, 1H), 9.68 (s, 1H), 7.62–7.59 (m, 1H), 7.38–7.35 (m, 1H), 7.30–7.13 (m, 10H), 7.08 (td, *J* = 7.0, 1.8 Hz, 1H), 6.97–6.93 (m, 1H), 4.49 (d, *J* = 11.2 Hz), 3.73–3.51 (m, 1H), 2.85–2.78 (m, 1H), 2.26–2.10 (m, 3H). ^13^C NMR (101 MHz, DMSO-*d*_6_) δ 173.4, 171.1, 152.2, 150.1, 138.5, 128.4, 128.3, 128.0, 126.7, 125.4, 124.7, 123.2, 122.7, 119.5, 119.1, 117.0, 110.9, 47.4, 47.0, 30.5, 28.5. HRMS (ESI) was calculated for C_26_H_23_N_2_O_3_ [M + H]^+^ 411.1703, found 411.1697.

### 3.3. General Procedure for the Synthesis of **7**

To a flame-dried Schlenk reaction tube equipped with a magnetic stir bar was added the catalyst (0.01 mmol), **1a** (0.10 mmol, 37.5 mg) and **2a** (0.10 mmol, 19.8 mg) under N_2_, and freshly distilled DCE (2.0 mL) was added. The mixture was then stirred at rt for 24 h. Then, the reaction mixture was filtered through a pad of Celite, and the solvent was concentrated in vacuo. Purification with flash column chromatography yielded product **7** as a white solid. ^1^H NMR (600 MHz, CDCl_3_) δ 7.85–7.83 (m, 2H), 7.82–7.79 (m, 1H), 7.44–7.33 (m, 7H), 7.33–7.26 (m, 4H), 7.26–7.23 (m, 1H), 7.16–7.11 (m, 1H), 7.09 (d, *J* = 8.0 Hz, 2H), 6.74 (s, 1H), 3.60 (s, 1H), 2.94–2.86 (m, 2H), 2.53 (ddd, *J* = 13.2, 7.9, 1.9 Hz, 1H), 2.35 (s, 3H), 2.34–2.28 (m, 1H), 1.96 (dddd, *J* = 14.3, 12.1, 5.5, 1.8 Hz, 1H). ^13^C NMR (151 MHz, CDCl_3_) δ 174.3, 169.5, 154.3, 151.1, 145.2, 138.0, 137.3, 134.4, 129.5, 129.1, 129.0, 128.5, 128.1, 125.6, 125.1, 124.5, 124.0, 120.8, 119.9, 118.5, 111.7, 47.4, 43.7, 32.9, 29.0, 21.7.

## 4. Conclusions

In summary, we have described a Lewis acid-promoted Michael addition/ring-expansion cascade of azadienes and cyclobutamines. Lewis acids were used to facilitate the cascade reactions. The entropic constraints of and unfavorable enthalpic changes in the classical end-to-end cyclization process were completely avoided. This process provides a new entry to access benzofuran-fused eight-membered lactams, which has emerged as a useful framework in drug discovery. The catalytic asymmetric version of the reaction has also been explored using chiral bases as catalysts, which may provide opportunities for stereoselective library collections of nitrogen-containing medium-sized rings.

## Figures and Tables

**Table 1:** Optimization of the reaction conditions. ^a^ Reaction conditions: **1a** (0.1 mmol), **2a** (0.11 mmol), base (0.2 mol), Lewis acid (0.02 mol), solvent (1.0 mL), Ts = 4-toluenesulfonyl, rt = room temperature. ^b^ Yields of isolated products. ^c^ **3aa** was obtained as mixture of atropisomers, only C_5_-C_6_ *trans* product was observed. Relative configuration. ^d^ dr was determined with ^1^H NMR analysis. ^e^ DIPEA = *N,N*-Diisopropylethylamine. ^f^ DMAP = 4-Dimethylaminopyridine. ^g^ DBN = 1,5-Diazabicyclo[4.3.0]-5-nonene. ^h^ DCE = 1,2-Dichloroethane. ^i^ The reaction was performed at −5 °C for 48 h.

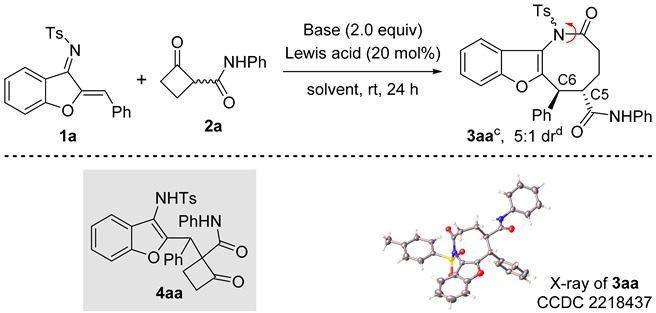				
Entry ^a^	Solvent	Base	Lewis Acid	Yield (%) ^b^
1	CH_2_Cl_2_	Et_3_N		16
2 ^e^	CH_2_Cl_2_	DIPEA		11
3 ^f^	CH_2_Cl_2_	DMAP		17
4 ^g^	CH_2_Cl_2_	DBN		NR
5	CH_2_Cl_2_	Cs_2_CO_3_		17
6	CH_2_Cl_2_	NaHCO_3_		26
7	CH_2_Cl_2_	K_2_CO_3_		27
8	CH_2_Cl_2_	KO^t^Bu		trace
9	CH_2_Cl_2_	KOH		trace
10	CH_2_Cl_2_	K_2_CO_3_	Mg(OTf)_2_	54
11	CH_2_Cl_2_	K_2_CO_3_	Sc(OTf)_2_	trace
12	CH_2_Cl_2_	K_2_CO_3_	Zn(OTf)_2_	41
13	CH_2_Cl_2_	K_2_CO_3_	Cu(OTf)_2_	11
14	THF	K_2_CO_3_	Mg(OTf)_2_	57
15 ^h^	DCE	K_2_CO_3_	Mg(OTf)_2_	63
16	MeCN	K_2_CO_3_	Mg(OTf)_2_	46
17	Toluene	K_2_CO_3_	Mg(OTf)_2_	18
**18 ^i^**	**DCE**	**K_2_CO_3_**	**Mg(OTf)_2_**	**78**

## Data Availability

Not applicable.

## Supplementary Materials

The following supporting information can be downloaded at: https://www.mdpi.com/article/10.3390/molecules28041650/s1, Section S1. General remarks, Section S2. General procedure for the preparation of substrates and chiral catalysts, Section S3. Characterization data of the unknown azadienes and eight-membered ring lactams, Section S4. Gram-scale reaction, Section S5. Loss of atropisomerism study, Section S6. VT ^1^H NMR experiments, Section S7. References [73,74,75,76,77,78,79,80], Section S8. X-ray report, Section S9. Copies of NMR spectra, Section S10. Optimization of the catalytic asymmetric reaction conditions and HPLC spectrum.
