# Supplementary material for: A Lewis Acid-Promoted Michael Addition and Ring-Expansion Cascade for the Construction of Nitrogen-Containing Medium-Sized Rings
Source: Molecules. 2023 Feb 8;28(4):1650. doi: 10.3390/molecules28041650 (PMC9966210; doi:10.3390/molecules28041650)
Supplement: Supplementary file 1 [file molecules-28-01650-s001.zip › molecules-2152598-supplementary.pdf]

Supplementary Materials

A Lewis Acid-Promoted Michael Addition and Ring-Expansion Cascade for the Construction of Nitrogen-Containing Medium-Sized Rings

Jiaming Wang, Jia Li and Changgui Zhao *

Key Laboratory of Radiopharmaceuticals, Ministry of Education, College of Chemistry, Beijing Normal University, Beijing 100875, China
* Correspondence: cgzhao@bnu.edu.cn; Tel.: +86-158-0115-4568

Table of Contents

1. General remarks... 2
2. General procedure for the preparation of substrates and chiral catalysts..................................... 2
3. Characterization data of the unknown azadienes and eight-membered ring lactams…………5
4. Gram-scale reaction.. 14
5. Loss of atropisomerism study.. 14
6. VT ^{1}H NMR experiments.. 15
7. References 16
8. X-ray report.. 17
9. Copies of NMR spectra.. 22
10. Optimization of the catalytic asymmetric reaction conditions and HPLC spectrum…..……46

1. General remarks

All reactions in non-aqueous media were conducted under a positive pressure of dry argon in glassware that had been dried in an oven prior to use unless noted otherwise. The anhydrous solutions of reaction mixtures were transferred via an oven-dried syringe or cannula. Chemicals were purchased from commercial sources, including dichloromethane (DCM), *n*-hexane, ethyl acetate (EA), methanol (MeOH), tetrahydrofuran (THF), acetone and petroleum ether (PE), and they were purchased from Beijing Chemical Factory (Beijing, China). Silica gel for analytical thin-layer chromatography (TLC) and column chromatography (200 ~ 300 mesh) was purchased from Qingdao Haiyang Chemical Co., Ltd & Special Silica Gel Factory. ^{1}H and ^{13}C nuclear magnetic resonance spectra (NMR) were obtained on a JEOL Delta (400 MHz and 600 MHz) recorded in ppm (δ) downfield of TMS (δ = 0) in $CDCl_3$ and DMSO-d_6 unless noted otherwise. Signal splitting patterns were described as singlet (s), doublet (d), triplet (t), quartet (q), quintet (quint) or multiplet (m), with coupling constants (*J*) in hertz. HPLC analysis was conducted on SHIMADZU LC-20ADXR instrument with chiral columns (Chiralpak IF, column 4.6 × 250 mm, (Daicel Chemical Ind., Ltd.)). High-resolution mass spectra (HRMS) were recorded on a Waters LCT Premier XE mass spectrometer with TOF. Crystallographic data were collected using a Rigaku Oxford Diffraction XtaLAB Synergy diffractometer equipped with a HyPix-6000E area detector at 173 K using Cu Kα (λ = 1.54184 Å) from a PhotonJet micro-focus X-ray source.

2. General procedure for the preparation of substrates and chiral catalysts

2.1 General procedure for the preparation of azadienes 1

Known azadiene **1a**, [73] **1b**, [73] **1e-1h**, [73] **1n**, [73] **1c**, [74] **1m**, [74] **1d**, [44] **1i**, [75] **1o**, [75] **1q**, [75] **1p**, [76] **1r** and [48] **1s** [49] were prepared according to the literature's procedure from commercially available materials. The spectra are in accordance with the literature.

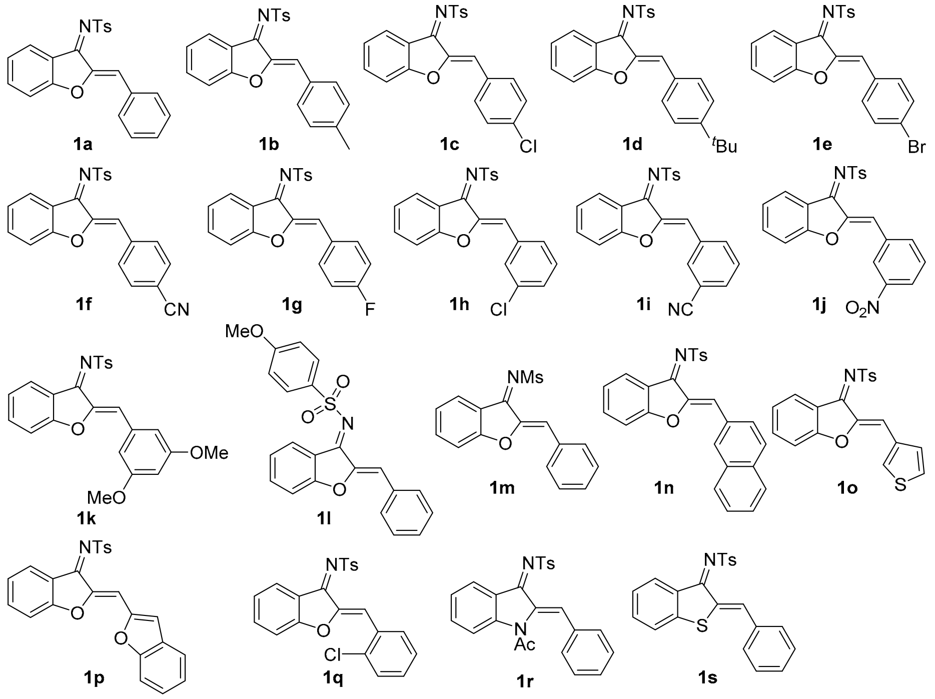

1j, 1k and **1l** were prepared using the following procedure.

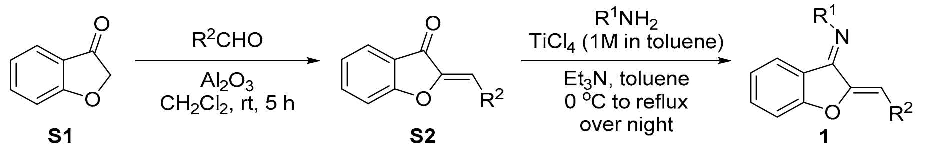

A round bottom flask was charged with a magnetic stirrer. To a solution of **S1** (1.5 g, 11.2 mmol, 1.0 equiv) in CH_2Cl_2 (40 mL), aromatic aldehyde (16.8 mmol, 1.5 equiv) and Al_2O_3 (activated basic, 36.6 g, 358 mmol, 32 equiv) were added. The reaction mixture was stirred for 8 hours at room temperature. Then, the reaction mixture was filtered through a pad of Celite®, and the filtrate was concentrated under reduced pressure. The final products were washed with petroleum ether and Et_2O to obtain the corresponding products: **S2.**

A two-neck round bottom flask was charged with a magnetic stirrer, **S2** (1.29 g, 5.8 mmol, 1.0 equiv), and *p*-toluenesulfonamide (1.49 g, 8.7 mmol, 1.5 equiv) was dissolved in toluene (40 mL) under a nitrogen atmosphere. Then, the reaction mixture was cooled to 0 °C. Triethylamine (1.62 mL, 11.6 mmol, 2.0 equiv) and $TiCl_4$ (1.0 M in toluene, 6.96 mL, 6.96 mmol, 1.2 equiv) were added dropwise. The reaction mixture was stirred for 15 hours at reflux. The solution was then cooled to room temperature and quenched with water (15 mL). The crude mixture was diluted with ethyl acetate (30 mL), washed with brine (3 × 30 mL) and dried over Na_2SO_4; the solvent evaporated under reduced pressure. The crude mixture was purified by column chromatography using silica gel (PE : CH_2Cl_2 : Et_3N = 60 : 30 : 1) to obtain the corresponding products: **1j-l**.

2.2. General procedure for the preparation of cyclobutanone carboxamides 2

Cyclobutanone carboxamides **2a, 2c-2f** and [62] **2g** [65] were prepared according to the literature's procedure from commercially available materials. The spectra are in accordance with the literature.

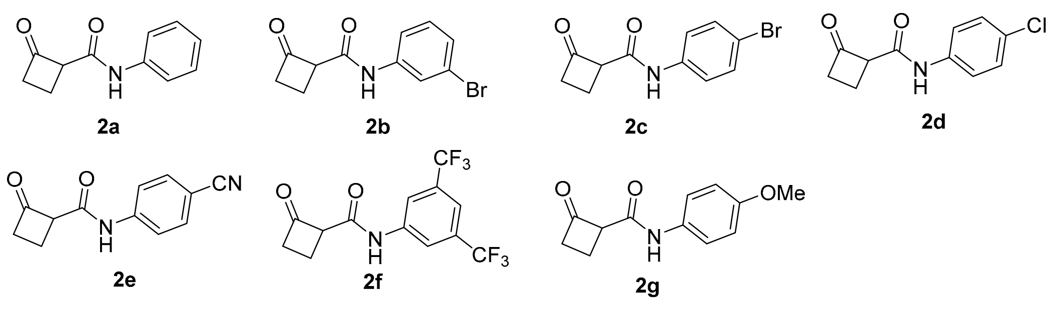

Cyclobutanone carboxamide **2b** was prepared using the following procedure.

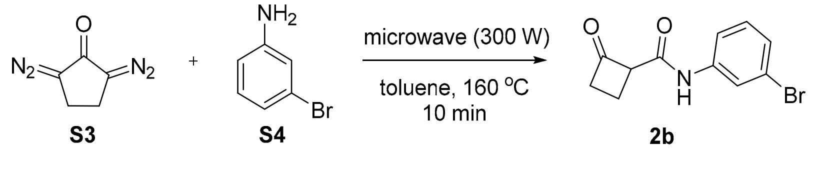

All reactions under microwave irradiation were performed at 300 W in a CEM Discover 1-300W system equipped with a built-in pressure measurement sensor and a vertically focused IR sensor (mode discover power time). A solution comprising 2-diazocyclopentane-1,3-dione **S3** (248 mg, 2.0 mmol, 1.0 equiv) and aromatic amines **S4** (340 mg, 2.0 mmol, 1.0 equiv) in dry toluene (3.0 mL) in a 5 mL microwave vial charged with a magnetic stir bar was used. The vial was sealed with a microwave crimp cap and heated to 160 °C for 10 minutes using a Biotage Initiator microwave reactor (sensitivity set to low). The vessel was then cooled to ambient temperatures, and the solvent evaporated under reduced pressure. The crude mixture was purified by column chromatography using silica gel (PE : EtOAc = 3 : 1) to obtain the corresponding product: **2b**.

2.3. General procedure for the preparation of chiral catalysts

Chiral catalysts **C2**,[77] **C3**,[77] **C5**,[77] **C7** and [78] **C10** [79] were prepared according to the literature's procedure from commercially available materials. The spectra are in accordance with the literature.

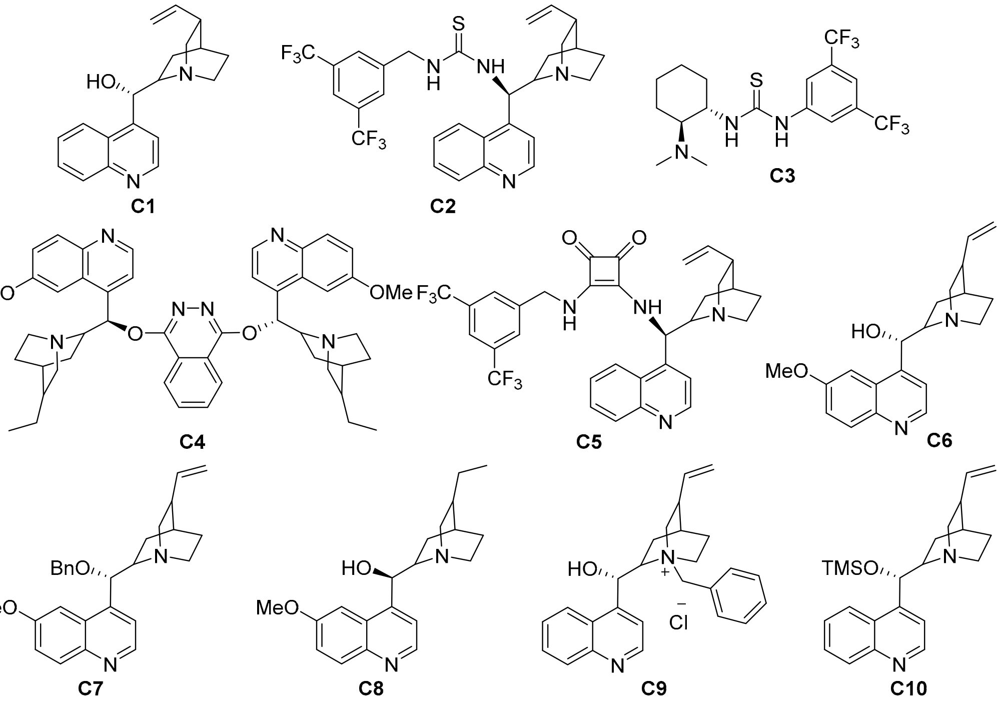

2.4. General procedure for the synthesis of 3

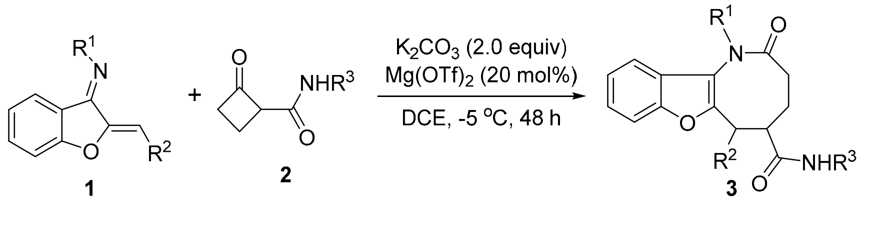

To an oven dried flask, **1** (0.1 mmol), **2** (0.11 mmol), K_2CO_3 (27.6 mg, 0.2 mmol), $Mg(OTf)_2$ (6.4 mg, 0.02 mmol) and dry DCE (1.5 mL) under Ar were added. The reaction mixture was stirred at -5 °C and monitored by TLC. After completion (~48 h), the reaction mixture was purified by flash column chromatography to yield the final product.

2.5. The assignment of relative configuration at C_5- and C_6- positions

The relative configuration of **3aa**, **3da** and **3qf** was confirmed by single-crystal X-ray diffraction analyses, and the other products were assigned by analogy. The diastereomers at C_5- and C_6-positions are separable by flash columns on silica gel. Generally, the C_5-C_6 *cis* product is more polar than the *trans* diastereoisomers at C_5- and C_6- positions. The atropoisomers are determined by ^1H NMR analyses. In the case of **3aa-3pa**, only a trace amount of cis products was observed by TLC analyses. ^1HNMR analysis of **3ka** and **3ma** suggested that no atropoisomers formed. The configuration of **3qa** was assigned by performing single-crystal X-ray diffraction analyses of analogy **3qf**. To further confirm the relative configuration, the H_aC_5–C_6H_b coupling constants in ^1HNMR experiments were calculated (Table 1). The coupling constants (^3J) of **3aa-3pa** (*trans* configuration, $\phi_{HaC5C6Hb}$ = 0.55°) were in the range from 11.8 Hz to 12.3 Hz, while the coupling constants (^3J) of **3qa-3qf** (*cis* configuration, $\phi_{HaC5C6Hb}$ = 80.40°) were 0 Hz. These results are consistent with the well-known Karplus equations [80].

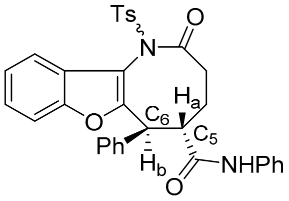

3aa

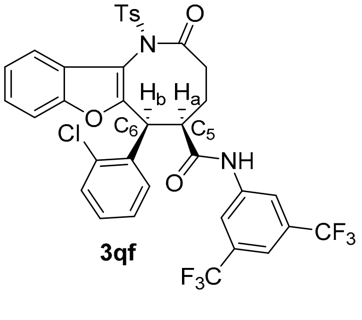

3qf

Table 1. Coupling constants (^3J) of **3aa-3pa**

Entry	J_{HaHb} (Hz)	Entry	J_{HaHb}(Hz)	Entry	J_{HaHb}(Hz)
**3ba**	11.8	**3ga**	11.8	**3la**	11.9
**3ca**	11.9	**3ha**	11.8	**3ma**	12.0
**3da**	11.8	**3ia**	11.9	**3na**	12.0
**3ea**	12.3	**3ja**	11.9	**3oa**	11.8
**3fa**	12.0	**3ka**	12.0	**3pa**	11.8

3. Characterization data of unknown azadienes and eight-membered ring lactams

3.1 Characterization data of azadienes 1j, 1k and 1l

1j, 4-methyl-*N*-((*Z*)-2-((*Z*)-3-nitrobenzylidene)benzofuran-3(2H)-ylidene)benzenesulfonamide

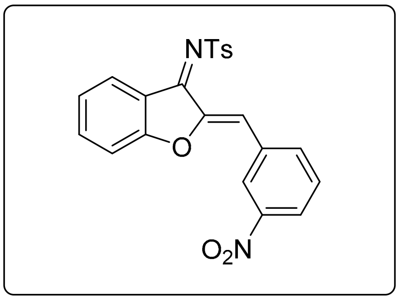

37%, yellow solid. **^{1}H NMR** (600 MHz, $CDCl_3$) δ 8.78 (d, *J* = 7.9 Hz, 1H), 8.28 – 8.24 (m, 2H), 8.02 – 7.97 (m, 4H), 7.72 (ddd, *J* = 8.5, 7.2, 1.4 Hz, 1H), 7.40 (d, *J* = 8.0 Hz, 2H), 7.37 – 7.31 (m, 2H), 7.06 (s, 1H), 2.49 (s, 3H). **^{13}C NMR** (151 MHz, $CDCl_3$) δ 164.8, 148.5, 147.8, 144.0, 138.7, 138.6, 138.3, 137.7, 131.9, 129.7, 127.3, 125.2, 124.6, 124.3, 124.1, 113.2, 112.5, 21.8. **HRMS** (ESI) calcd. for $C_{22}H_{17}N_2O_5S$ $[M + H]^+$ 421.0853, found 421.0860.

1k, *N*-((*Z*)-2-((*Z*)-3,5-dimethoxybenzylidene)benzofuran-3(2H)-ylidene)-4-methylbenzenesulfonamide

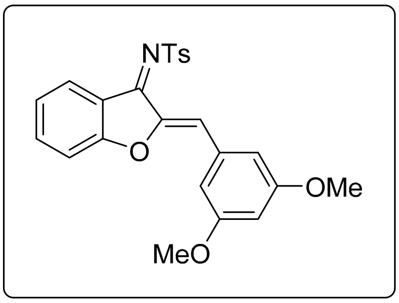

42%, yellow solid. **^{1}H NMR** (600 MHz, $CDCl_3$) NMR (600 MHz, $CDCl_3$) δ 8.76 (d, *J* = 8.0 Hz, 1H), 8.04 – 7.93 (m, 2H), 7.66 (ddd, *J* = 8.3, 7.2, 1.4 Hz, 1H), 7.37 (d, *J* = 8.0 Hz, 2H), 7.29 (d, *J* = 8.3 Hz, 1H), 7.28 – 7.25 (m, 1H), 7.03 (d, *J* = 2.3 Hz, 2H), 7.01(s, 1H), 6.51 (t, *J* = 2.3 Hz, 1H), 3.82 (s, 6H), 2.46 (s, 3H). **^{13}C NMR** (151 MHz, $CDCl_3$) δ 165.2, 164.9, 161.0, 150.0, 143.6, 139.1, 137.8, 137.8, 134.0, 131.2, 129.6, 127.2, 124.0, 118.4, 112.5, 109.7, 103.0, 55.6, 21.8. **HRMS** (ESI) calcd. for $C_{24}H_{22}N_2O_5S$ $[M + H]^+$ 436.1213, found 436.1220.

1l, *N*-((*E*)-2-((*Z*)-benzylidene)benzofuran-3(2H)-ylidene)-4-methoxybenzenesulfonamide

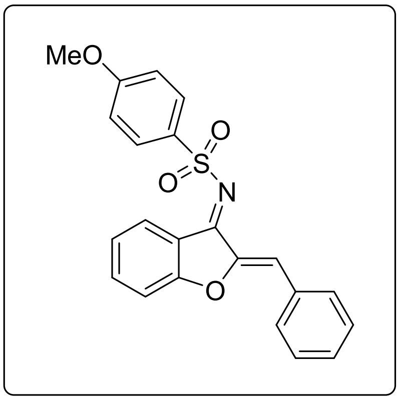

42%, yellow solid. **^{1}H NMR** (600 MHz, $CDCl_3$) δ 8.78 (d, *J* = 8.2 Hz, 1H), 8.07 – 8.02 (m, 2H), 7.89 – 7.81 (m, 2H), 7.64 (ddt, *J* = 8.5, 7.2, 1.7 Hz, 1H), 7.40 (td, *J* = 7.9, 7.3, 1.8 Hz, 2H), 7.35 (td, *J* = 7.1, 1.7 Hz, 1H), 7.29 (dd, *J* = 8.5, 1.8 Hz, 1H), 7.27 – 7.22 (m, 1H), 7.08 (s, 1H), 7.02 (dd, *J* = 8.6, 1.3 Hz, 2H), 3.86 (s, 3H). **^{13}C NMR** (151 MHz, $CDCl_3$) δ 165.0, 164.8, 163.0, 149.7, 137.6, 133.8, 132.5, 131.7, 131.2, 130.3, 129.2, 129.0, 123.8, 118.4, 115.6, 114.1, 112.4, 55.7. **HRMS** (ESI) calcd. for $C_{22}H_{18}NO_4S$ $[M + H]^+$ 392.0951, found 392.0947.

3.2 Characterization data of 2b

2b, ***N*-(3-bromophenyl)-2-oxocyclobutane-1-carboxamide**

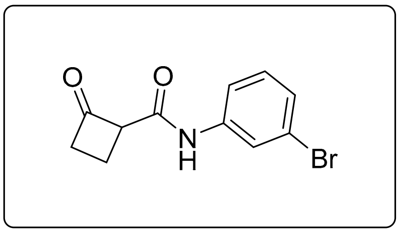

22%, yellow oil. **^{1}H NMR** (400 MHz, $CDCl_3$) δ 8.00 (s, 1H), 7.98(s, 1H), 7.78 – 7.76 (m, 1H), 7.34 – 7.27 (m, 1H), 7.11 – 7.04 (m, 1H), 4.32 – 4.26 (m, 1H), 3.28 – 3.18 (m, 1H), 3.14 – 3.05 (m, 1H), 2.57 – 2.33 (m, 2H). **^{13}C NMR** (101 MHz, $CDCl_3$) δ 205.6, 171.4, 139.1, 130.5, 127.8, 123.0, 118.6, 45.9, 36.2, 20.6. **HRMS** (ESI) calcd. for $C_{11}H_{11}BrNO_2$ $[M + H]^+$ 267.9968, found 267.9976.

3.3 Characterization data of eight-membered ring lactams

3aa, **2-oxo-*N*,6-diphenyl-1-tosyl-1,2,3,4,5,6-hexahydro-benzofuro[3,2-*b*]azocine-5-car boxam ide**

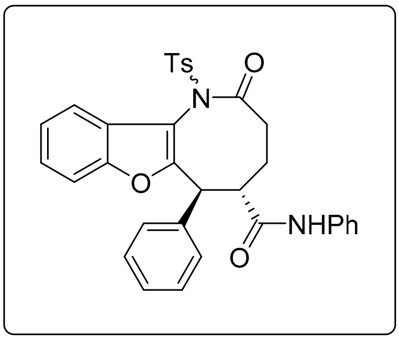

78%, white solid. **^{1}H NMR** (600 MHz, $CDCl_3$) δ 8.26 (d, J = 7.8 Hz, 2H), 7.50 – 7.38 (m, 5H), 7.36 – 7.27 (m, 3H), 7.22 – 7.13 (m, 5H), 7.05 – 6.96 (m, 3H), 6.59 (s, 1H), 4.57 (d, J = 11.8 Hz, 1H), 3.22 (t, J = 12.0 Hz, 1H), 2.83 – 2.67 (m, 1H), 2.45 (s, 3H), 2.39 – 2.17 (m, 3H). **^{13}C NMR** (101 MHz, $CDCl_3$) δ 173.2, 170.4, 153.5, 146.1, 138.9, 136.6, 135.9, 130.2, 129.6, 129.1, 128.9, 128.7, 127.9, 126.1, 125.6, 125.1, 123.8, 120.9, 120.6, 119.3, 116.3, 111.9, 48.9, 47.9, 33.4, 29.4, 21.9. **HRMS** (ESI) calcd. for $C_{33}H_{29}N_2O_5S$ $[M + H]^+$ 565.1792, found 565.1893.

3ba, **2-oxo-*N*-phenyl-6-(p-tolyl)-1-tosyl-1,2,3,4,5,6-hexahydro-benzofuro[3,2-*b*]azocine-5-car boxamide**

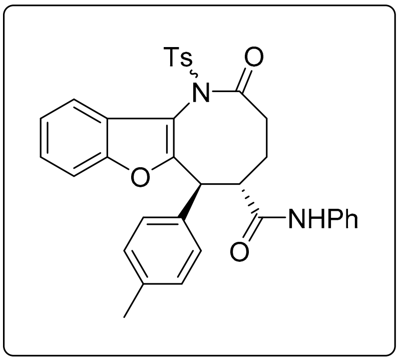

67%, white solid. **^{1}H NMR** (600 MHz, $CDCl_3$) δ 8.30 (d, J = 8.3 Hz, 2H), 7.48 – 7.46 (m, 3H), 7.38 (d, J = 8.0 Hz, 3H), 7.33 – 7.28 (m, 2H), 7.23 (t, J = 7.7 Hz, 2H), 7.11 – 7.08 (m, 5H), 6.71 (s, 1H), 4.58 (d, J = 11.8 Hz, 1H), 3.28 – 3.19 (m, 1H), 2.83 – 2.73 (m, 1H), 2.51 (s, 3H), 2.41 – 2.27 (m, 3H), 2.25 (s, 3H). **^{13}C NMR** (151 MHz, $CDCl_3$) δ 172.6, 169.8, 155.1, 152.8, 145.4, 136.9, 136.2, 135.1, 129.5, 129.0, 128.9, 128.1, 127.8, 125.3, 124.7, 124.1, 123.0, 120.0, 118.5, 115.4, 111.2, 47.8, 47.1, 32.8, 28.7, 21.2, 20.4. **HRMS** (ESI) calcd. for $C_{34}H_{21}N_2O_5S$ $[M + H]^+$ 579.1948, found 579.1957.

3ca, **6-(4-chlorophenyl)-2-oxo-*N*-phenyl-1-tosyl-1,2,3,4,5,6-hexahydro-benzofuro[3,2-*b*]azocine-5-carboxamide**

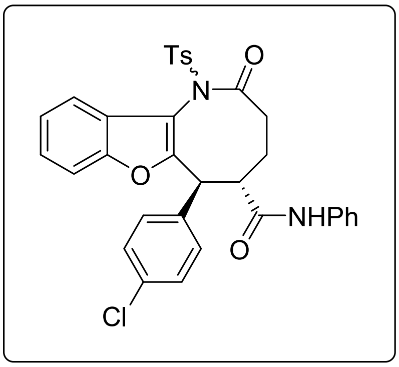

67%, white solid. **^{1}H NMR** (600 MHz, $CDCl_3$) δ 8.27 (d, *J* = 8.0 Hz, 2H), 7.47 (dd, *J* = 13.1, 8.6 Hz, 4H), 7.41 – 7.38 (m, 2H), 7.35 – 7.26 (m, 4H), 7.23 (s, 1H), 7.19 – 7.10 (m, 4H), 7.07 (t, *J* = 7.7 Hz, 1H), 4.63 (d, *J* = 11.9 Hz, 1H), 3.32 (t, *J* = 11.5 Hz, 1H), 2.77 – 2.72 (m, 1H), 2.52 (s, 3H), 2.40 – 2.26 (m, 3H). **^{13}C NMR** (151 MHz, $CDCl_3$) δ 173.1, 170.1, 155.1, 153.5, 146.2, 137.3, 136.7, 135.7, 133.8, 130.1, 129.6, 129.1, 128.9, 125.9, 125.7, 125.0, 123.8, 120.7, 119.2, 116.3, 111.9, 108.2, 48.0, 47.7, 33.4, 29.4, 21.8. **HRMS** (ESI) calcd. for $C_{33}H_{28}ClN_2O_5S$ $[M + H]^+$ 599.1402, found 599.1410.

3da, **6-(4-chlorophenyl)-2-oxo-*N*-phenyl-1-tosyl-1,2,3,4,5,6-hexahydro-benzofuro[3,2-*b*] azocine-5-carboxamide**

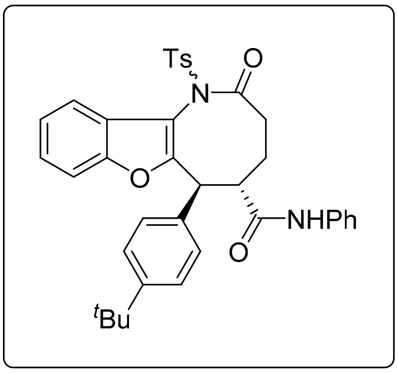

84%, white solid. **^{1}H NMR** (600 MHz, $CDCl_3$) δ 8.29 (d, *J* = 7.7 Hz, 2H), 7.47 (d, *J* = 7.8 Hz, 2H), 7.44 (d, *J* = 7.2 Hz, 1H), 7.41 – 7.31 (m, 3H), 7.32 – 7.28 (m, 4H), 7.18 (t, *J* = 7.8 Hz, 2H), 7.04 (t, *J* = 7.5 Hz, 1H), 6.96 (d, *J* = 7.8 Hz, 2H), 6.66 (s, 1H), 4.55 (d, *J* = 11.8 Hz, 1H), 3.26 (t, *J* = 11.9 Hz, 1H), 2.79 – 2.74 (m, 1H), 2.49 (s, 3H), 2.41 – 2.28 (m, 3H), 1.21 (s, 9H). **^{13}C NMR** (101 MHz, $CDCl_3$) δ 173.4, 170.6, 155.8, 153.6, 150.8, 146.13, 136.7, 136.0, 136.0, 130.3, 129.7, 128.8, 128.2, 126.2, 126.2, 125.5, 125.1, 123.8, 121.2, 119.3, 116.2, 112.0, 48.8, 47.9, 34.6, 33.5, 31.3, 29.2, 21.9. **HRMS** (ESI) calcd. for $C_{37}H_{37}N_2O_5S$ $[M + H]^+$ 621.2418, found 621.2410.

3ea, **6-(4-bromophenyl)-2-oxo-*N*-phenyl-1-tosyl-1,2,3,4,5,6-hexahydro-benzofuro[3,2-*b*] azocine-5-carboxamide**

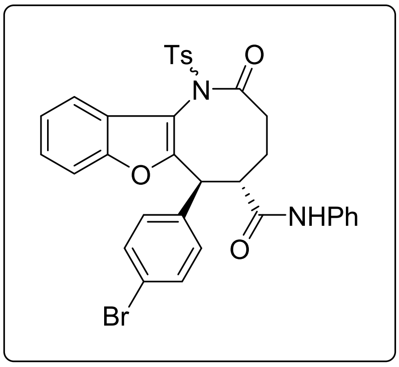

63%, white solid. **^{1}H NMR** (600 MHz, $CDCl_3$) δ 8.28 (d, *J* = 6.6 Hz, 2H), 7.51 – 7.48 (m, 2H), 7.43 (m, 4H), 7.40 (m, 2H), 7.36 – 7.28 (m, 2H), 7.27 (d, *J* = 1.8 Hz, 1H), 7.24 (d, *J* = 1.7 Hz, 1H), 7.17 – 7.13 (m, 2H), 7.12 – 7.07 (m, 1H), 6.98 (s, 1H), 4.63 (d, *J* = 12.3 Hz, 1H), 3.38 – 3.26 (m, 1H), 2.87 – 2.68 (m, 1H), 2.52 (s, 3H), 2.35 (m, 3H). **^{13}C NMR** (151 MHz, $CDCl_3$) δ 173.2, 170.2, 155.1, 153.5, 146.3, 137.9, 136.7, 135.8, 132.2, 130.5, 130.2, 129.7, 125.9, 125.7, 125.1, 123.9, 122.1, 120.8 119.3, 116.3, 112.0, 48.1, 47.6, 33.5, 29.5, 21.9. **HRMS** (ESI) calcd. for $C_{33}H_{28}BrN_2O_5S$ $[M + H]^+$ 643.0879, found 643.0886.

3fa, **6-(4-bromophenyl)-2-oxo-*N*-phenyl-1-tosyl-1,2,3,4,5,6-hexahydro-benzofuro[3,2-*b*] azocine-5-carboxamide**

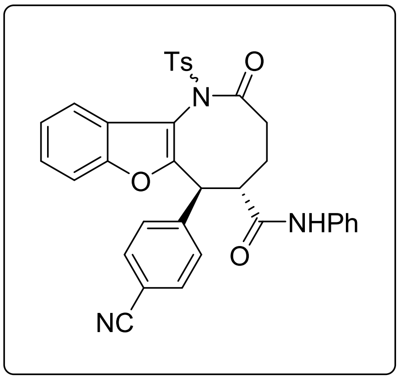

63%, white solid. **^{1}H NMR** (400 MHz, $CDCl_3$) δ 8.26 (d, *J* = 8.0 Hz, 2H), 7.72 – 7.56 (m, 4H), 7.48 (d, *J* = 8.1 Hz, 2H), 7.42 – 7.29 (m, 6H), 7.22 – 7.15 (m, 3H), 7.11 – 7.01 (m, 1H), 4.74 (d, *J* = 12.0 Hz, 1H), 3.50 – 3.43 (m, 1H), 2.82 – 2.64 (m, 1H), 2.52 (s, 3H), 2.40 – 2.28 (m, 2H), 2.08 – 2.05 (m, 1H). **^{13}C NMR** (151 MHz, $CDCl_3$) δ 173.2, 167.0, 154.7, 153.7, 146.5, 144.2, 136.9, 132.8, 132.1, 130.1, 129.9, 129.8, 129.1, 126.0, 125.7, 125.2, 124.1, 120.5, 119.3, 116.6, 112.1, 111.8, 48.5, 47.2, 33.6, 29.6, 22.0. **HRMS** (ESI) calcd. for $C_{34}H_{28}N_3O_5S$ $[M + H]^+$ 590.1744, found 590.1751.

3ga, **6-(4-fluorophenyl)-2-oxo-*N*-phenyl-1-tosyl-1,2,3,4,5,6-hexahydro-benzofuro[3,2-*b*] azocine-5-carboxamide**

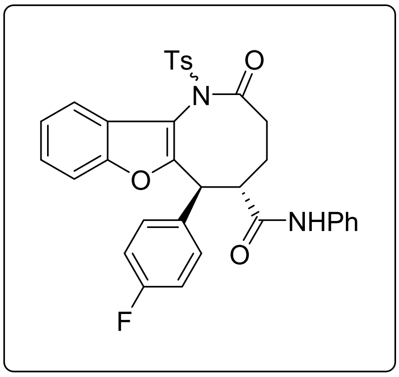

84%, white solid. **^{1}H NMR** (600 MHz, $CDCl_3$) δ 8.24 (d, *J* = 8.2 Hz, 2H), 7.55 – 7.40 (m, 4H), 7.35–7.31 (m, 3H), 7.28–7.27 (m, 1H), 7.24–7.22 (m, 2H), 7.22–7.20 (m, 3H), 7.10 (d, *J* = 7.6 Hz, 1H), 7.05 – 7.01 (m, 1H), 6.78 (s, 1H), 4.81 (d, *J* = 11.8 Hz, 1H), 3.20 (td, *J* = 11.5, 3.0 Hz, 1H), 2.78 – 2.68 (m, 1H), 2.50 (s, 3H), 2.38 – 2.24 (m, 3H). **^{13}C NMR** (101 MHz, $CDCl_3$) δ 173.2, 170.3, 155.5, 153.5, 146.2, 136.7, 135.7, 134.7, 134.6, 130.4, 130.4, 130.1, 129.6, 128.9, 125.9, 125.6, 125.1, 123.8, 120.6, 119.2, 116.1, 116.0, 115.8, 111.9, 47.9, 47.8, 33.4, 29.3, 21.8. **HRMS** (ESI) calcd. for $C_{34}H_{28}FN_2O_5S$ $[M + H]^+$ 583.1697, found 583.1690.

3ha, **6-(3-chlorophenyl)-2-oxo-*N*-phenyl-1-tosyl-1,2,3,4,5,6-hexahydro-benzofuro[3,2-*b*] azocine-5-carboxamide**

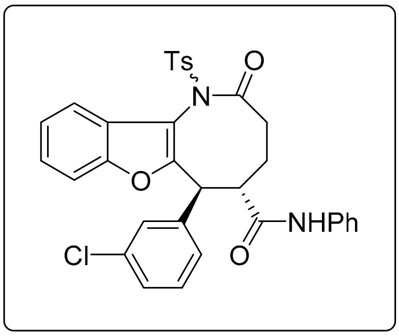

51%, white solid. **^{1}H NMR** (400 MHz, $CDCl_3$) δ 8.29 (d, *J* = 8.0 Hz, 2H), 7.54 (s, 1H), 7.49 – 7.44 (m, 3H), 7.41 – 7.39 (m, 2H), 7.35 – 7.28 (m, 2H), 7.23 – 7.16 (m, 4H), 7.12 (d, *J* = 8.0 Hz, 2H), 7.07 – 7.04 (m, 1H), 6.82 (s, 1H), 4.60 (d, *J* = 11.8 Hz, 1H), 3.22 (t, *J* = 11.1 Hz, 1H), 2.86 – 2.67 (m, 1H), 2.50 (s, 3H), 2.40 – 2.25 (m, 3H). **^{13}C NMR** (151 MHz, $CDCl_3$) δ 173.0, 169.9, 154.6, 153.4, 146.1, 140.6, 136.5, 134.7, 130.2, 130.1, 130.0, 129.5, 129.3, 128.8, 128.7, 128.0, 126.8, 125.8, 125.6, 125.0, 123.8, 120.4, 119.3, 111.8, 48.2, 47.5, 33.3, 29.3, 21.8. **HRMS** (ESI) calcd. for $C_{32}H_{28}ClN_2O_5S$ $[M + H]^+$ 599.1402, found 599.1410.

3ia, **6-(3-cyanophenyl)-2-oxo-*N*-phenyl-1-tosyl-1,2,3,4,5,6-hexahydro-benzofuro[3,2-*b*] azocine-5-carboxamide**

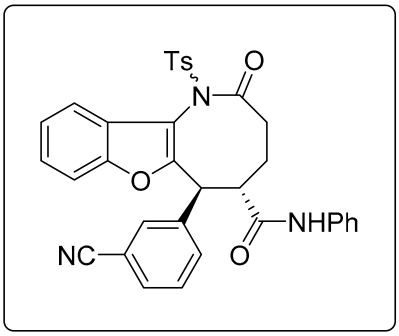

62%, white solid. **^{1}H NMR** (400 MHz, $CDCl_3$) δ 8.28 (d, *J* = 7.6 Hz, 2H), 7.88 (s, 1H), 7.82 (d, *J* = 8.9 Hz, 1H), 7.54 – 7.48 (m, 3H), 7.44 – 7.33 (m, 7H), 7.20 – 7.15 (m, 2H), 7.10 – 7.05 (m, 2H), 4.72 (d, *J* = 11.9 Hz, 1H), 3.46 – 3.37 (m, 1H), 2.87 – 2.71 (m, 1H), 2.53 (s, 3H), 2.43 – 2.33 (m, 3H). **^{13}C NMR** (101 MHz, $CDCl_3$) δ 173.1, 169.9, 154.5, 153.7, 146.4, 140.5, 136.7, 135.8, 133.2, 132.8, 131.7, 130.1, 129.9, 129.1, 126.0, 125.8, 125.3, 124.1, 120.6, 120.3, 119.4, 118.5, 116.7, 113.1, 112.1, 48.2, 47.41, 33.5, 29.6, 22.0. **HRMS** (ESI) calcd. for $C_{34}H_{28}N_3O_5S$ $[M + H]^+$ 590.1744, found 590.1750.

3ja, **6-(3-nitrophenyl)-2-oxo-*N*-phenyl-1-tosyl-1,2,3,4,5,6-hexahydro-benzofuro[3,2-*b*] azocine-5-carboxamide**

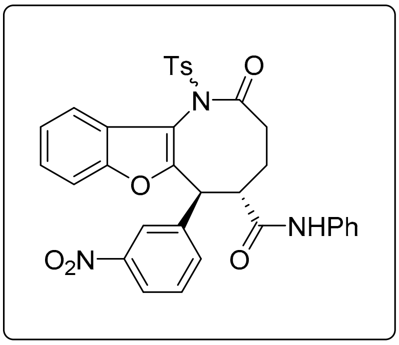

51%, white solid. **^{1}H NMR** (400 MHz, $CDCl_3$) δ 8.28 (d, *J* = 8.6 Hz, 2H), 8.16 (d, *J* = 8.9 Hz, 2H), 7.76 (d, *J* = 8.8 Hz, 2H), 7.50 (d, *J* = 8.3 Hz, 2H), 7.46 – 7.32 (m, 5H), 7.24 – 7.15 (m, 4H), 7.12 – 7.01 (m, 1H), 4.82 (d, *J* = 11.9 Hz, 1H), 3.62 – 3.42 (m, 1H), 2.86 – 2.68 (m, 1H), 2.54 (s, 3H), 2.42 – 2.34 (m, 2H), 1.99 (d, *J* = 5.2 Hz, 1H). **^{13}C NMR** (151 MHz, $CDCl_3$) δ 172.8, 169.7, 154.2, 153.5, 147.3, 146.3, 145.9, 136.5, 135.5, 129.9, 129.8, 129.7, 128.9, 125.9, 125.5, 125.1, 124.0, 123.9, 120.3, 119.1, 116.5, 111.9, 48.1, 47.1, 33.3, 29.6, 21.8. **HRMS** (ESI) calcd. for $C_{32}H_{28}N_3O_7S$ $[M + H]^+$ 610.1642, found 610.1637.

3ka, **6-(3,5-dimethoxyphenyl)-2-oxo-*N*-phenyl-1-tosyl-1,2,3,4,5,6-hexahydro-benzofuro[3,2-*b*] azocine-5-carboxamide**

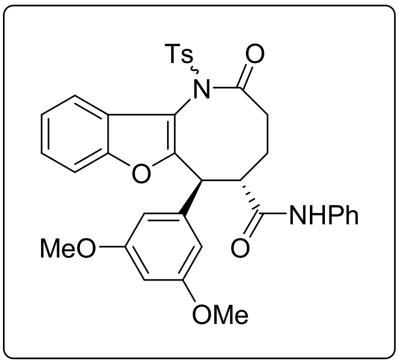

63%, white solid. **^{1}H NMR** (600 MHz, $CDCl_3$) δ 8.30 (d, *J* = 8.1 Hz, 2H), 7.49 (d, *J* = 8.2 Hz, 2H), 7.45 – 7.41 (m, 2H), 7.34 (td, *J* = 8.2, 7.7, 1.5 Hz, 1H), 7.32 – 7.28 (m, 1H), 7.24 (t, *J* = 7.9 Hz, 2H), 7.20 – 7.16 (m, 2H), 7.10 – 7.05 (m, 2H), 6.70 (d, *J* = 2.3 Hz, 2H), 6.29 (t, *J* = 2.3 Hz, 1H), 4.57 (d, *J* = 12.0 Hz, 1H), 3.69 (s, 6H), 3.35 (td, *J* = 11.6, 2.8 Hz, 1H), 2.80 – 2.70 (m, 1H), 2.52 (s, 3H), 2.42 – 2.29 (m, 3H). **^{13}C NMR** (101 MHz, $CDCl_3$) δ 173.4, 170.6, 161.3, 155.8, 153.6, 146.2, 141.1, 137.1, 135.9, 130.3, 130.1, 129.7, 129.5, 128.9, 126.0, 125.6, 124.9, 123.9, 120.9, 119.1, 116.3, 112.1, 106.3, 101.0, 55.8, 49.2, 47.9, 33.5, 29.1, 22.0. **HRMS** (ESI) calcd. for $C_{35}H_{33}N_2O_7S$ $[M + H]^+$ 625.2003, found 625.2009.

3la, **1-((4-methoxyphenyl)sulfonyl)-2-oxo-*N*,6-diphenyl-1,2,3,4,5,6-hexahydro-benzofuro[3,2-*b*]azocine-5-carboxamide**

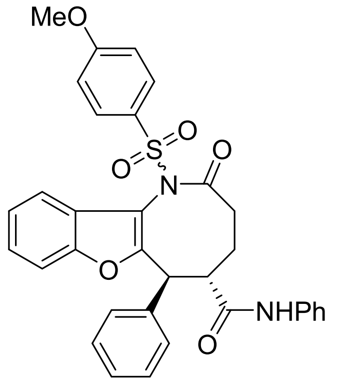

63%, white solid. **^{1}H NMR** (400 MHz, $CDCl_3$) δ 8.35 (d, *J* = 9.0 Hz, 2H), 7.52 – 7.44 (m, 3H), 7.40 – 7.36 (m, 1H), 7.33 – 7.27 (m, 4H), 7.22 – 7.18 (m, 3H), 7.13 – 7.04 (m, 5H), 6.67 (s, 1H), 4.61 (d, *J* = 11.9 Hz, 1H), 3.91 (s, 3H), 3.23 (td, *J* = 11.8, 2.8 Hz, 1H), 2.85 – 2.73 (m, 1H), 2.46 – 2.23 (m, 3H). **^{13}C NMR** (101 MHz, $CDCl_3$) δ 173.3, 170.5, 164.7, 155.5, 153.5, 139.0, 136.9, 132.7, 130.0, 129.1, 129.0, 128.7, 128.0, 126.2, 125.6, 125.0, 123.8, 120.8, 119.3, 116.5, 114.2, 112.0, 56.0, 48.9, 47.9, 33.5, 29.5. **HRMS** (ESI) calcd. for $C_{33}H_{29}N_2O_6S$ $[M + H]^+$ 581.1741, found 581.1747.

3ma, **1-(methylsulfonyl)-2-oxo-*N*,6-diphenyl-1,2,3,4,5,6-hexahydro-benzofuro[3,2-*b*] azocine-5-carboxamide**

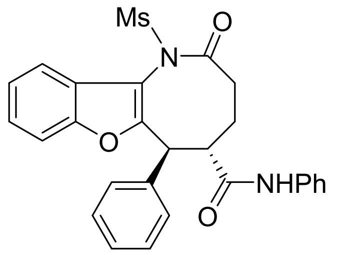

47%, white solid. **^{1}H NMR** (400 MHz, $CDCl_3$) δ 7.44 – 7.36 (m, 4H), 7.35 – 7.27 (m, 4H), 7.22 – 7.15 (m, 3H), 7.12 – 7.02 (m, 3H), 6.98 (s, 1H), 4.63 (d, *J* = 12.0 Hz, 1H), 3.84 (s, 3H), 3.41 (td, *J* = 11.5, 3.7 Hz, 1H), 2.90 (td, *J* = 11.7, 8.2 Hz, 1H), 2.56 – 2.36 (m, 3H). **^{13}C NMR** (151 MHz, $CDCl_3$) δ 174.8, 170.4, 156.1, 153.5, 138.9, 136.8, 129.2,128.8, 128.5, 127.9 125.6, 125.6, 124.9, 123.8, 120.9, 118.7, 114.9, 112.1, 48.9, 47.9, 44.5, 33.3, 29.1. **HRMS** (ESI) calcd. for $C_{27}H_{25}N_2O_5S$ $[M + H]^+$ 489.1479, found 489.1486.

3na, **6-(naphthalen-2-yl)-2-oxo-*N*-phenyl-1-tosyl-1,2,3,4,5,6-hexahydro-benzofuro[3,2-*b*] azo cine-5-carboxamide**

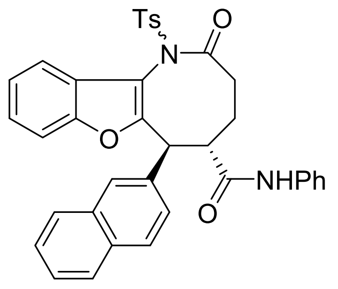

78%, white solid. **^{1}H NMR** (400 MHz, $CDCl_3$) δ 8.33 (d, *J* = 7.2 Hz, 2H), 8.01 (s, 1H), 7.83 – 7.71 (m, 3H), 7.67 – 7.60 (m, 1H), 7.51 – 7.48 (m, 1H), 7.45 (d, *J* = 7.9 Hz, 2H), 7.42 – 7.37 (m, 2H), 7.36 – 7.34 (m, 1H), 7.32 – 7.29 (m, 2H), 7.17 – 7.07 (m, 2H), 7.00 – 6.86 (m, 3H), 6.63 (s, 1H), 4.80 (d, *J* = 12.0 Hz, 1H), 3.34 (t, *J* = 11.7 Hz, 1H), 2.89 – 2.74 (m, 1H), 2.48 (s, 3H), 2.46 – 2.29 (m, 3H). **^{13}C NMR** (101 MHz, $CDCl_3$) δ 173.3, 170.5, 155.6, 153.6, 146.2, 136.6, 136.4, 136.0, 133.6, 132.9, 130.3, 129.7, 128.9, 128.8, 128.3, 128.0, 127.7, 126.4, 126.3, 125.7, 125.0, 123.9, 120.9, 119.4, 116.6, 112.0, 47.0, 48.1, 33.6, 29.5, 21.9. **HRMS** (ESI) calcd. for $C_{37}H_{31}N_2O_5S$ $[M + H]^+$ 615.1948, found 615.1957.

3oa, **2-oxo-*N*-phenyl-6-(thiophen-2-yl)-1-tosyl-1,2,3,4,5,6-hexahydro-benzofuro[3,2-*b*]azocine-5-carboxamide**

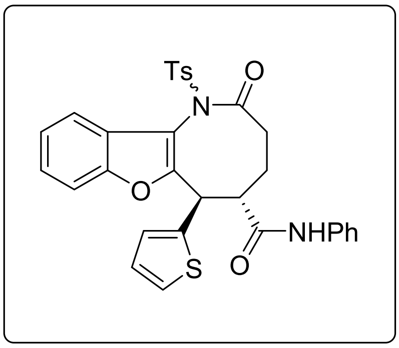

76%, white solid. **^{1}H NMR** (600 MHz, $CDCl_3$) δ 8.25 (d, *J* = 8.2 Hz, 2H), 7.50 – 7.42 (m, 4H), 7.39 – 7.30 (m, 3H), 7.28 – 7.26 (m, 1H), 7.25 – 7.24 (m, 1H), 7.22 – 7.20 (m, 3H), 7.12 – 7.01 (m, 2H), 6.79 (s, 1H), 4.82 (d, *J* = 11.8 Hz, 1H), 3.21 (td, *J* = 11.5, 3.0 Hz, 1H), 2.78 – 2.69 (m, 1H), 2.50 (s, 3H), 2.38 – 2.24 (m, 3H). **^{13}C NMR** (151 MHz, $CDCl_3$) δ 173.2, 170.8, 155.5, 153.5, 146.1, 139.3, 137.0, 130.2, 129.6, 129.0, 127.7, 126.5, 125.6, 125.0, 123.9, 120.8, 119.3, 115.8, 112.0, 48.0, 43.8, 33.5, 29.0, 21.9. **HRMS** (ESI) calcd. for $C_{31}H_{27}N_2O_5S_2$ $[M + H]^+$ 571.1356, found 571.1350.

3pa, **6-(benzofuran-2-yl)-2-oxo-*N*-phenyl-1-tosyl-1,2,3,4,5,6-hexahydro-benzofuro[3,2-*b*] azocine-5-carboxamide**

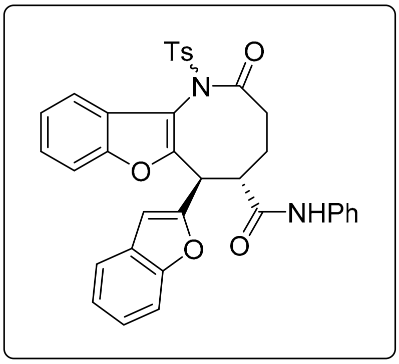

73%, white solid. **^{1}H NMR** (400 MHz, $CDCl_3$) δ 8.36 (d, *J* = 8.3 Hz, 2H), 7.70 – 7.64 (m, 1H), 7.49 – 7.33 (m, 8H), 7.23 – 7.13 (m, 4H), 7.13 – 7.06 (m, 1H), 7.06 – 7.00 (m, 2H), 6.71 (s, 1H), 4.89 (d, *J* = 11.8 Hz, 1H), 2.98 (td, *J* = 11.8, 3.2 Hz, 1H), 2.72 (td, *J* = 12.0, 8.2 Hz, 1H), 2.43 (s, 3H), 2.39 – 2.17 (m, 3H). **^{13}C NMR** (101 MHz, $CDCl_3$) δ 172.7, 170.1, 155.0, 153.6, 153.5, 151.2, 146.1, 136.8, 135.8, 130.6, 129.4, 129.0, 128.2, 126.4, 126.0, 125.1, 124.7, 124.2, 123.3, 121.5, 120.6, 120.2, 117.5, 111.9, 111.0, 106.0, 45.5, 42.6, 33.3, 28.9, 21.9. **HRMS** (ESI) calcd. for $C_{31}H_{27}N_2O_5S_2$ $[M + H]^+$ 571.1356, found 571.1350.

3qa, **6-(2-chlorophenyl)-2-oxo-*N*-phenyl-1-tosyl-1,2,3,4,5,6-hexahydro-benzofuro[3,2-*b*] azocine-5-carboxamide**

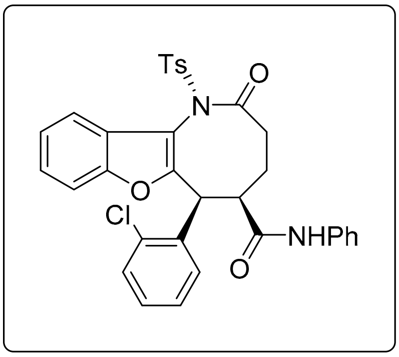

95%, white solid. **^{1}H NMR** (400 MHz, $CDCl_3$) δ 8.33 (d, *J* = 5.1 Hz, 1H), 7.84 (d, *J* = 6.7 Hz, 1H), 7.70 (d, *J* = 7.9 Hz, 2H), 7.46 – 7.26 (m, 10H), 7.13 (t, *J* = 7.3 Hz, 1H), 6.94 (d, *J* = 8.0 Hz, 2H), 6.57 (s, 1H), 3.82 (s, 1H), 2.85 (t, *J* = 12.6 Hz, 1H), 2.77 (d, *J* = 5.5 Hz, 1H), 2.55 (dd, *J* = 12.8, 7.7 Hz, 1H), 2.33 (s, 3H), 2.25 (dd, *J* = 14.8, 7.7 Hz, 1H), 2.03 (td, *J* = 14.6, 13.8, 5.5 Hz, 1H) **^{13}C NMR** (101 MHz, $CDCl_3$) δ 174.4, 169.7, 154.8, 150.5, 145.5, 137.5, 136.2, 134.6, 133.8, 130.3, 130.0, 129.6, 129.5, 129.2, 128.9, 127.5, 126.1, 125.0, 124.6, 124.4, 121.2, 119.8, 119.4, 111.8, 46.7, 39.9, 33.1 28.4, 21.8. **HRMS** (ESI) calcd. for $C_{32}H_{28}ClN_2O_5S$ $[M + H]^+$ 599.1402, found 599.1409.

3qb, ***N*-(3-bromophenyl)-6-(2-chlorophenyl)-2-oxo-1-tosyl-1,2,3,4,5,6-hexahydro-benzofuro [3,2-*b*]azocine-5-carboxamide**

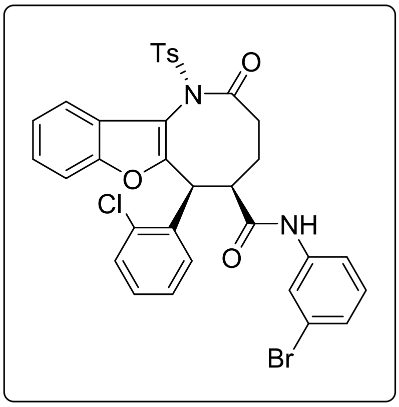

71%, white solid. **^{1}H NMR** (600 MHz, $CDCl_3$) δ 8.33 – 8.28 (m, 1H), 7.88 – 7.84 (m, 1H), 7.70 (d, *J* = 8.2 Hz, 2H), 7.57 (s, 1H), 7.46 – 7.33 (m, 6H), 7.30 – 7.27 (m, 1H), 7.17 (t, *J* = 8.0 Hz, 1H), 7.10 (dt, *J* = 8.5, 1.2 Hz, 1H), 6.94 (d, *J* = 8.1 Hz, 2H), 6.56 (s, 1H), 3.80 (d, *J* = 1.4 Hz, 1H), 2.85 – 2.79 (m, 1H), 2.78 (d, *J* = 5.8 Hz, 1H), 2.56 (ddd, *J* = 13.1, 7.9, 1.8 Hz, 1H), 2.33 (s, 3H), 2.24 (ddt, *J* = 14.8, 8.0, 1.8 Hz, 1H), 2.05 (dddd, *J* = 14.4, 12.7, 5.8, 1.8 Hz, 1H). **^{13}C NMR** (151 MHz, $CDCl_3$) δ 173.5, 169.2, 154.0, 149.5, 144.8, 138.0, 135.3, 133.9, 132.9, 129.7, 129.1, 128.9, 128.7, 128.1, 126.8, 126.8, 125.5, 124.2, 123.7, 122.1, 120.5, 118.8, 117.4, 111.0, 46.0, 39.1, 32.3, 27.5, 21.03. **HRMS** (ESI) calcd. for $C_{33}H_{27}BrClN_2O_5S$ $[M + H]^+$ 677.0507, found 677.0517.

3qc, ***N*-(4-bromophenyl)-6-(2-chlorophenyl)-2-oxo-1-tosyl-1,2,3,4,5,6-hexahydro-benzofuro [3,2-*b*]azocine-5-carboxamide**

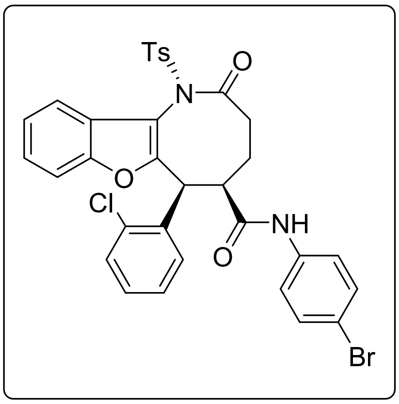

80%, white solid. **^{1}H NMR** (400 MHz, $CDCl_3$) δ 8.32 – 8.25 (m, 1H), 7.89 – 7.81 (m, 1H), 7.69 (d, *J* = 8.1 Hz, 2H), 7.46 – 7.36 (m, 7H), 7.32 – 7.27 (m, 1H), 7.19 – 7.13 (m, 2H), 6.93 (d, *J* = 8.2 Hz, 2H), 6.59 (s, 1H), 3.80 (s, 1H), 2.86 – 2.74 (m, 2H), 2.62 – 2.50 (m, 1H), 2.33 (s, 3H), 2.24 (dd, *J* = 14.7, 7.9 Hz, 1H), 2.03 (td, *J* = 13.0, 5.6 Hz, 1H). **^{13}C NMR** (101 MHz, $CDCl_3$) δ 174.3, 169.8 154.7, 136.6, 136.0, 134.6, 133.7, 132.2, 130.4, 129.9, 129.7, 129.5, 128.9, 127.5, 126.2, 124.9, 124.5, 121.3, 121.2, 119.5, 117.1, 111.7, 46.7, 39.8, 33.1, 28.3, 21.8. **HRMS** (ESI) calcd. for $C_{33}H_{27}BrClN_2O_5S$ $[M + H]^+$ 677.0507, found 677.0516.

3qd, *N*-(2-chlorophenyl)-6-(2-chlorophenyl)-2-oxo-1-tosyl-1,2,3,4,5,6-hexahydro-benzofuro [3,2-*b*]azocine-5-carboxamide

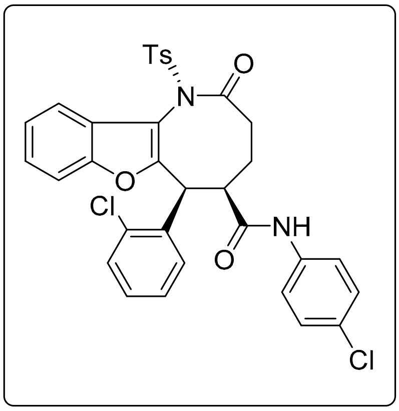

72%, white solid. **^{1}H NMR** (600 MHz, $CDCl_3$) δ 8.34 – 8.27 (m，1H), 7.88 – 7.81 (m, 1H), 7.69 (d, *J* = 8.3 Hz, 2H), 7.45 – 7.35 (m, 5H), 7.32 – 7.26 (m, 3H), 7.22 (d, *J* = 8.8 Hz, 2H), 6.94 (d, *J* = 8.0 Hz, 2H), 6.60 (s, 1H), 3.80 (s, 1H), 2.82 (t, *J* = 12.3 Hz, 1H), 2.77 (d, *J* = 5.6 Hz, 1H), 2.55 (ddd, *J* = 13.1, 7.9, 1.5 Hz, 1H), 2.33 (s, 3H), 2.24 (dd, *J* = 14.7, 8.0 Hz, 1H), 2.03 (dddd, *J* = 14.4, 12.4, 5.6, 1.7 Hz, 1H). **^{13}C NMR** (151 MHz, $CDCl_3$) δ 174.3, 169.8, 154.7, 150.4, 145.6, 136.1, 136.1, 134.7, 133.7, 130.5, 129.9, 129.7, 129.5, 129.5, 129.2, 128.9, 127.5, 126.2, 125.0, 124.5, 121.3, 120.9, 119.5, 111.7, 46.7, 39.9, 33.1, 28.3, 21.8. **HRMS** (ESI) calcd. for $C_{33}H_{27}Cl_2N_2O_5S$ $[M + H]^+$ 633.1012, found 633.1017.

3qe, 6-(2-chlorophenyl)-*N*-(4-cyanophenyl)-2-oxo-1-tosyl-1,2,3,4,5,6-hexahydro-benzofuro[3,2-*b*]azocine-5-carboxamide

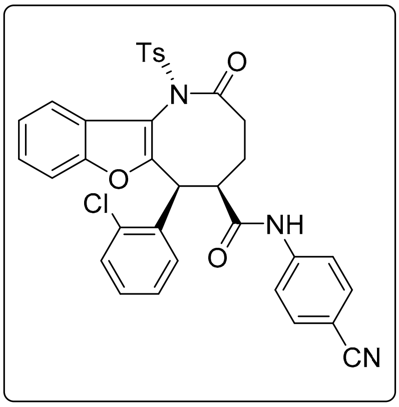

72%, white solid. **^{1}H NMR** (400 MHz, $CDCl_3$) δ 8.25 – 8,23 (m, 1H), 7.88 – 7.78 (m, 1H), 7.67 (d, *J* = 7.9 Hz, 2H), 7.58 (d, *J* = 8.3 Hz, 2H), 7.46 – 7.33 (m, 7H), 7.23 – 7.15 (m, 1H), 6.92 (d, *J* = 8.0 Hz, 2H), 6.86 (s, 1H), 3.79 (s, 1H), 2.82 (d, *J* = 5.3 Hz, 1H), 2.77 (t, *J* = 12.6 Hz, 1H), 2.54 (dd, *J* = 13.0, 7.7 Hz, 1H), 2.32 (s, 3H), 2.23 (dd, *J* = 14.7, 7.7 Hz, 1H), 2.04 (td, *J* = 16.3, 14.6, 5.6 Hz, 1H). **^{13}C NMR** (101 MHz, $CDCl_3$) δ 174.1, 170.4, 154.7, 150.1, 145.6, 141.5, 135.8, 134.7, 133.6, 133.5, 130.5, 129.8, 129.7, 129.5, 128.9, 127.5, 126.3, 124.9, 124.6, 121.3, 119.6, 119.4, 118.8, 111.5, 107.4, 46.9, 39.8, 33.0, 28.2, 21.8. **HRMS** (ESI) calcd. for $C_{34}H_{27}ClN_3O_5S$ $[M + H]^+$ 624.1354, found 624.1354.

3qf, *N*-(3,5-bis(trifluoromethyl)phenyl)-6-(2-chlorophenyl)-2-oxo-1-tosyl-1,2,3,4,5,6-hexahydro-benzofuro[3,2-*b*]azocine-5-carboxamide

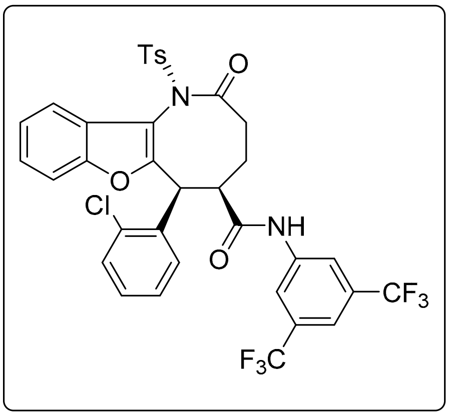

79%, white solid. **^{1}H NMR** (400 MHz, $CDCl_3$) δ 8.35 – 8.17 (m, 1H), 7.92 – 7.83 (m, 1H), 7.76 (s, 2H), 7.72 – 7.63 (m,3H), 7.50 – 7.34 (m, 5H), 7.24 (d, *J* = 5.6 Hz, 1H), 6.93 (d, *J* = 8.0 Hz, 2H), 6.88 (s, 1H), 3.80 (s, 1H), 2.89 – 2.73 (m, 2H), 2.57 (dd, *J* = 13.0, 7.7 Hz, 1H), 2.33 (s, 3H), 2.25 (dd, *J* = 14.7, 7.7 Hz, 1H), 2.08 (td, *J* = 14.1, 13.3, 5.3 Hz, 1H).**^{13}C NMR** (101 MHz, $CDCl_3$) δ 174.1, 170.5, 154.7, 150.0, 145.6, 138.8, 135.8, 134.7, 131.6 (q, *J* =33.3 Hz, 2C), 129.9, 129.6, 129.4, 128.8, 127.5, 126.3, 124.6, 123.1 (q, *J* = 273.7 Hz, 2C), 121.30, 119.40, 117.8, 111.5, 46.8, 39.8, 33.0, 28.0, 21.8. **HRMS** (ESI) calcd. for $C_{35}H_{26}ClF_6N_3O_5S$ $[M + H]^+$ 735.1150, found 735.1157.

4. Gram-scale reaction

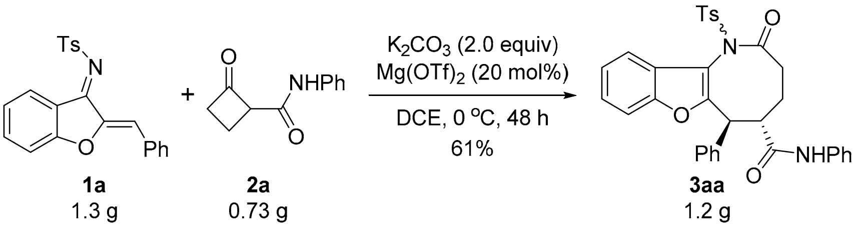

In total, **1a** (1.2 g, 3.2 mmol, 1.0 equiv), **2a** (0.73 g, 3.7 mmol, 1.15 equiv), K_2CO_3 (0.88 g, 6.4 mmol, 2.0 equiv) and $Mg(OTf)_2$ (238 mg, 0.74 mmol, 0.2 equiv) were dissolved in DCE (50 mL) at 0 °C. The reaction mixture was constantly stirred at this temperature for 48 h, and then the reaction mixture was extracted with EtOAc (3 × 20 mL), and the organic layer was washed by brine, dried

over Na_2SO_4 and then concentrated at reduced pressure to produce a residue, which was subjected to flash chromatography on silica gel to obtain the corresponding product, **3aa** (1.2 g, 61%), as a white solid.

5. Loss of atropisomerism study

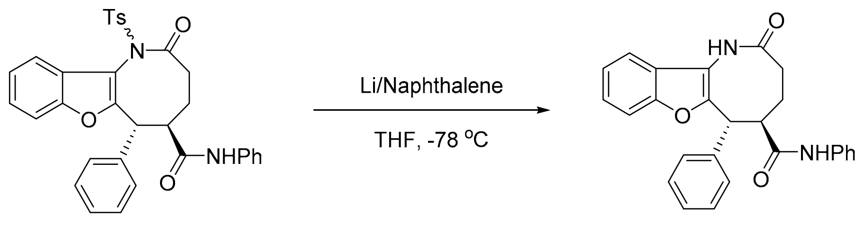

3aa

6, 84% yield
no atropisomerism

Naphthalene (448 mg, 3.5 mmol, 1.0 equiv) was dissolved in THF (7 mL) under N_2. Lithium (25 mg, 3.5 mmol, 1.0 equiv) was added, and the mixture stirred for 2 h at room temperature. A dark green Li/ Naphthalene solution (0.5 M) was obtained. To an over dried flask, **3aa** (56 mg, 0.1 mmol, 1.0 equiv) and dry THF (1.5 mL) under Ar at -78 °C were added. The Li/Naphthalene solution (0.5 M in THF) was added dropwise until the dark green solution turned colorless. Then, the reaction mixture was stirred at RT for 30 min. Quenched by 1 M $NaHCO_3$ (5 mL), the aqueous layer was extracted with EtOAc (3 × 10 mL), and the combined organic layer was dried by Na_2SO_4, filtered and concentrated in vacuo. Then, it was purified by flash column chromatography to yield the final product, **6** (84% yield), as a white solid. **^{1}H NMR** (400 MHz, DMSO-d_6) δ 9.87 (s, 1H), 9.68(s, 1H), 7.62 – 7.59 (m, 1H), 7.38 – 7.35 (m, 1H), 7.30 – 7.13 (m, 10H), 7.08 (td, J = 7.0, 1.8 Hz, 1H), 6.97 – 6.93 (m, 1H), 4.49 (d, J = 11.2 Hz), 3.73 – 3.51 (m, 1H), 2.85 – 2.78 (m, 1H), 2.26 – 2.10 (m, 3H). **^{13}C NMR** (101 MHz, DMSO-d_6) δ 173.4, 171.1, 152.2, 150.1, 138.5, 128.4, 128.3, 128.0, 126.7, 125.4, 124.7, 123.2, 122.7, 119.5, 119.1, 117.0, 110.9, 47.4, 47.0, 30.5, 28.5. **HRMS** (ESI) calcd. for $C_{26}H_{23}N_2O_3$ $[M + H]^+$ 411.1703, found 411.1697.

6. VT ^{1}H NMR experiments

^{1}H NMR of a solution of **3aa** in DMSO-d_6 was recorded at various temperatures (368 K, 358 K, 348 K, 338 K, 328 K, 318 K, 308 K and 298 K).

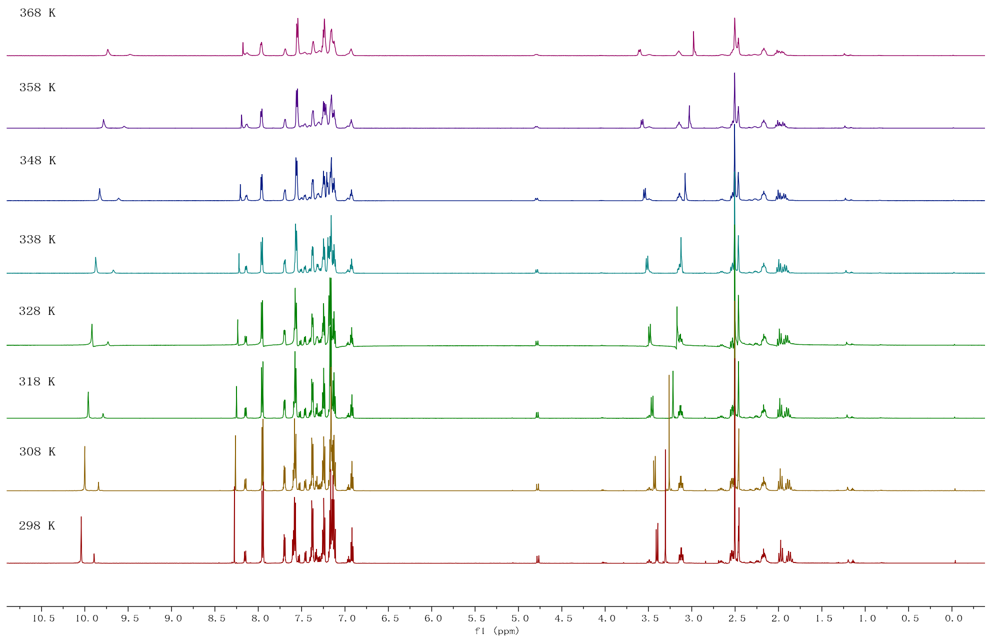

Full spectrum of 3aa

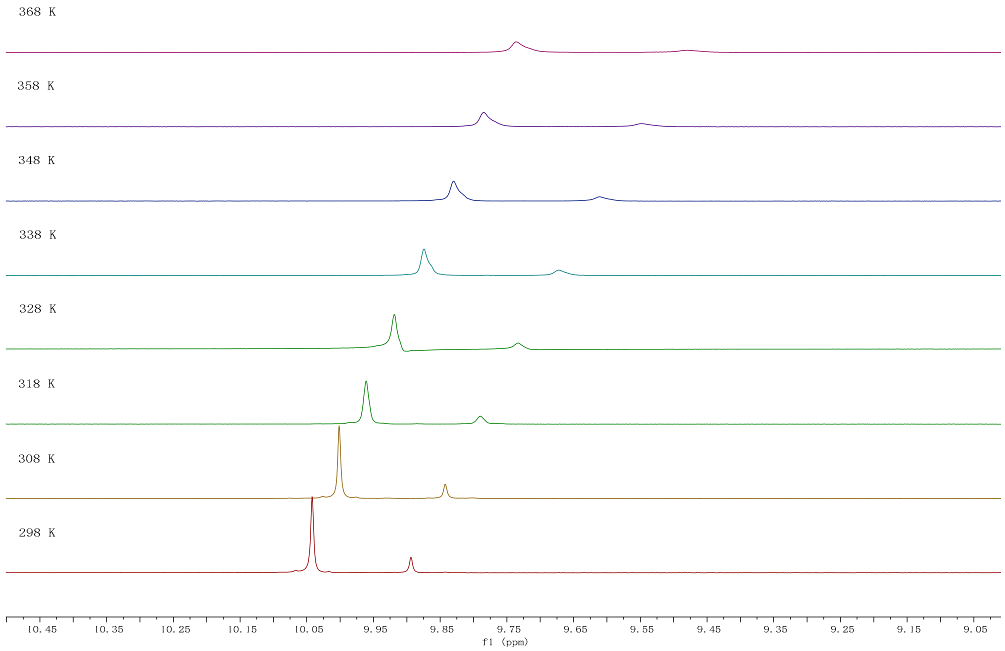

Zoom on 9.0 – 10.5 ppm

7. References

[44] Scuiller, A.; Karnat, A.; Casaretto, N.; Archambeau, A. Vinylcyclopropanes as all-carbon 1,5-dipoles: A reactivity switch for Palladium-catalyzed (5+4) cycloadditions. *Org. Lett.* **2021**, *23*, 2332–2336.

[48] Ni, H.; Tang, X.; Zheng, W.; Yao, W.; Ullah, N.; Lu, Y. Enantioselective phosphine-catalyzed formal [4+4] annulation of α,β-unsaturated imines and allene ketones: Construction of eight-membered rings. *Angew. Chem. Int. Ed.* **2017**, *56*, 14222–14226.

[49] Jiang, B.; Du, W.; Chen, Y.-C. Modified cinchona alkaloid-catalysed enantioselective [4+4] annulations of cyclobutenones and 1-azadienes. *Chem. Commun.* **2020**, *56*, 7257–7260.

[62] Zhou, Y.; Wei, Y.-L.; Rodriguez, J.; Coquerel, Y. Enantioselective organocatalytic four-atom ring expansion of cyclobutanones: Synthesis of benzazocinones. *Angew. Chem. Int. Ed.* **2019**, *58*, 456–460.

[65] Yang, W.-L.; Wang, Y.-L.; Li, W.; Gu, B.-M.; Wang, S.-W.; Luo, X.; Tian, B.-X.; Deng, W.-P. Diastereo- and enantioselective synthesis of eight-membered heterocycles via an allylation/ring expansion sequence enabled by multiple catalysis. *ACS Catal.* **2021**, *11*, 12557–12564.

[73] Laina-Martín,V.; Humbrías-Martín, J.; Mas-Ballesté, R.; Fernández-Salas, J.A.; Alemán, J. Enantioselective Inverse-Electron Demand Aza-Diels–Alder Reaction: ipso,α-Selectivity of Silyl Dienol Ethers. *ACS Catal.* **2021**, *11*, 12133-12145.

[74] Trost, B.M.; Zuo, Z. Highly Regio-, Diastereo-, and Enantioselective Synthesis of Tetrahydroazepines and Benzo[b]oxepines through Palladium-Catalyzed [4+3] Cycloaddition Reactions. *Angew. Chem. Int. Ed.* **2020**, *59*, 1243-1247.

[75] Qin, X.-Y.; Meng, F.-T.; Wang, M.; Tu, S.-J.; Hao, W.-J.; Wang, J.; Jiang, B. Gold-Catalyzed Skeletal Rearrangement of Alkenes: Regioselective Synthesis of Skeletally Diverse Tricyclic Heterocycles and Mechanistic Investigations. *ACS Catal.* **2021**, *11*, 6951-6959.

[76] Liu, K.; Yang, J.; Li, X. Palladium-Catalyzed Diastereo- and Enantioselective [3 + 2] Cycloaddition of Vinylcyclopropanes with Azadienes: Efficient Access to Chiral Spirocycles. *Org. Lett.* **2021**, *23*, 826-831.

[77] Mailhol, D.M.; Duque, d.M.S.; Raimondi, W.; Bonne, D.; Constantieux, T.; Coquerel, Y.; Rodriguez, J. Enantioselective Organocatalytic Michael Addition of Cyclobutanones to Nitroalkenes. *Adv. Synth. & Catal.* **2012**, *354*, 3523-3532.

[78] He, P.; Liu, X.; Shi, J.; Lin, L.; Feng, X. Organocatalytic Sequential Michael Reactions: Stereoselective Synthesis of Multifunc-tionalized Tetrahydroindan Derivatives. *Org. Lett.* **2011**, *13*, 936-939.

[79] Sheppard, C. I.; Taylor, J. L.; Wiskur, S. L. Silylation-Based Kinetic Resolution of Monofunctional Secondary Alcohols. *Org. Lett.* **2011**, *13*, 3794-3797.

[80] Karplus, M. Vicinal Proton Coupling in Nuclear Magnetic Resonance. *J. Am. Chem. Soc.* **1963**, *85*, 2870–2871.

8. X-Ray report

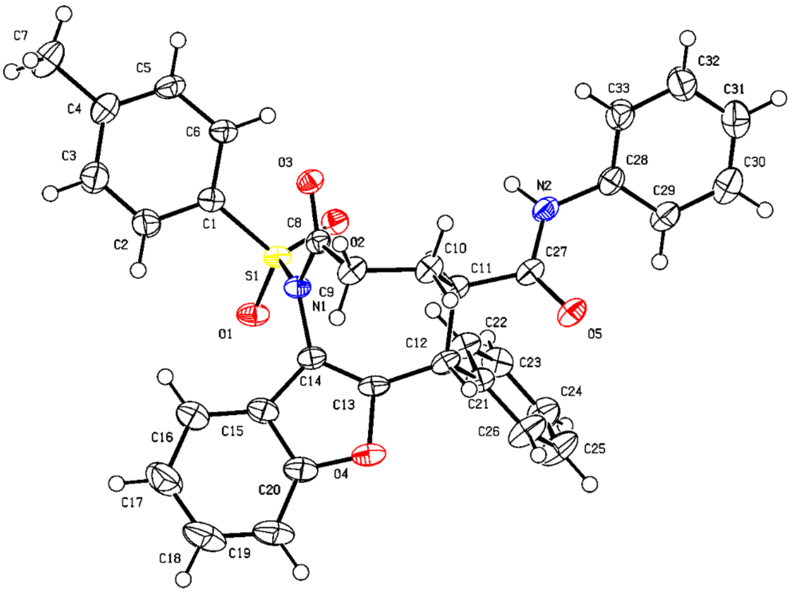

Figure S1. Thermal ellipsoid plot for the crystal structure of 3aa (at 50% probability level, CCDC No. 2218437)

Experimental

Single crystals of $C_{33}H_{28}N_2O_5S$ (**3aa**) were recrystallised from *n*-hexane, ethyl acetate (EA) and petroleum ether (PE) mounted in inert oil and transferred to the cold gas stream of the diffractometer.

Table S1 Crystal data and structure refinement for exp_wjm-20220317-1_auto.

Identification code	exp_wjm-20220317-1_auto
Empirical formula	$C_{33}H_{28}N_2O_5S$
Formula weight	564.63
Temperature/K	100.00(10)
Crystal system	monoclinic
Space group	C2/c
a/Å	29.9406(7)
b/Å	7.50522(18)
c/Å	29.3684(7)
α/	90
β/	100.471(2)
γ/	90
Volume/Å^3	6489.5(3)
Z	8
ρ_{calc}g/cm^3	1.156

μ/mm^{-1}	1.211
F(000)	2368.0
Crystal size/mm^3	0.2 × 0.1 × 0.1
Radiation	CuKα (λ = 1.54184)
2Θ range for data collection/	9.326 to 152.956
Index ranges	-37 ≤ h ≤ 37, -8 ≤ k ≤ 8, -37 ≤ l ≤ 35
Reflections collected	25767
Independent reflections	6473 [R_{int} = 0.0907, R_{sigma} = 0.0773]
Data/restraints/parameters	6473/0/371
Goodness-of-fit on F^2	1.134
Final R indexes [I>=2σ (I)]	R_1 = 0.0558, wR_2 = 0.1542
Final R indexes [all data]	R_1 = 0.0721, wR_2 = 0.1658
Largest diff. peak/hole / e Å^{-3}	0.28/-0.56

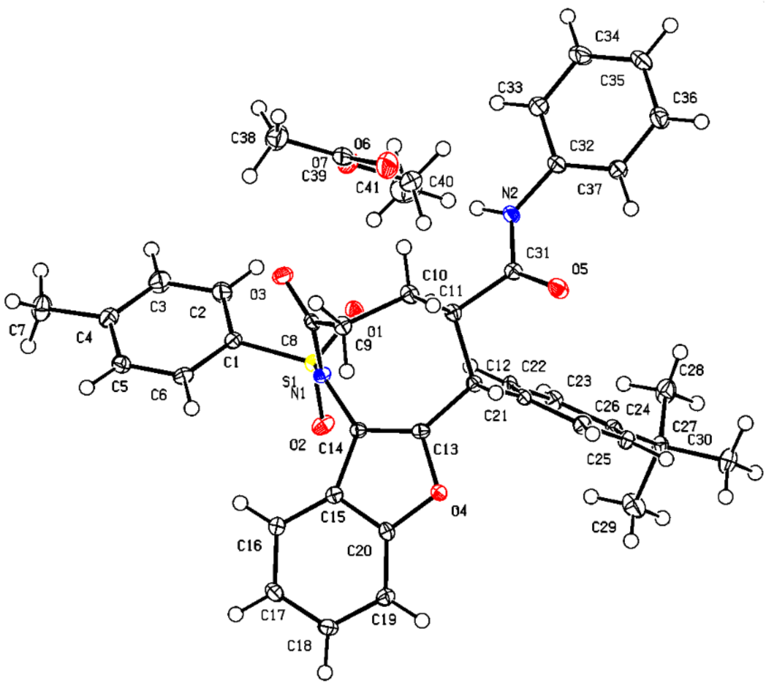

Figure S2. Thermal ellipsoid plot for the crystal structure of 3da (at 50% probability level, CCDC No. 2218433)

Experimental

Single crystals of $C_{41}H_{44}N_2O_7S$ (**3da**) were recrystallised from *n*-hexane, ethyl acetate (EA) and petroleum ether (PE) mounted in inert oil and transferred to the cold gas stream of the diffractometer.

Table S2 Crystal data and structure refinement for exp_10928-J Wang-ii-61-11_auto.

Identification code	exp_10928-J Wang-ii-61-11_auto
Empirical formula	$C_{41}H_{44}N_2O_7S$
Formula weight	708.84
Temperature/K	100.00(10)
Crystal system	triclinic
Space group	P-1
a/Å	7.8383(3)
b/Å	11.2972(4)
c/Å	20.8528(6)
α/	78.643(3)
β/	83.899(3)
γ/	77.996(3)
Volume/Å3	1766.68(11)
Z	2
ρcalcg/cm3	1.333
μ/mm-1	0.147
F(000)	752.0
Crystal size/mm3	0.15 × 0.15 × 0.15
Radiation	MoKα (λ = 0.71073)
2Θ range for data collection/°	6.514 to 62.15
Index ranges	-11 ≤ h ≤ 10, -14 ≤ k ≤ 14, -29 ≤ l ≤ 28
Reflections collected	31278
Independent reflections	8997 [Rint = 0.0401, Rsigma = 0.0446]
Data/restraints/parameters	8997/0/466
Goodness-of-fit on F2	1.068
Final R indexes [I>=2σ (I)]	R1 = 0.0421, wR2 = 0.0995
Final R indexes [all data]	R1 = 0.0584, wR2 = 0.1053
Largest diff. peak/hole / e Å-3	0.61/-0.43

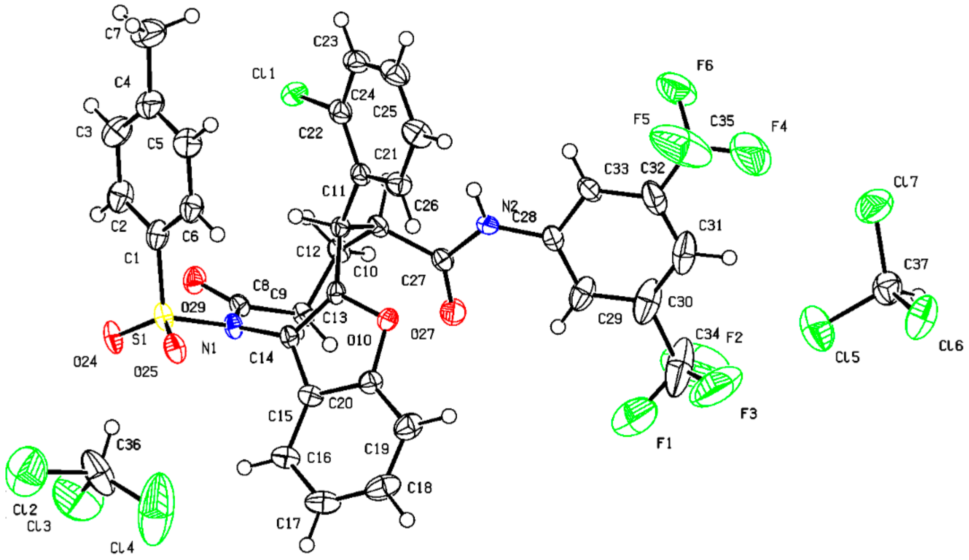

Figure S3. Thermal ellipsoid plot for the crystal structure of 3qf (at 50% probability level, CCDC No. 2218434)

Experimental

Single crystals of $C_{37}H_{27}Cl_7F_6N_2O_5S$ (**3da**) were recrystallised from $CDCl_3$ mounted in inert oil and transferred to the cold gas stream of the diffractometer.

Table S3 Crystal data and structure refinement for exp_12307_autored

Identification code	exp_12307_autored
Empirical formula	$C_{37}H_{27}Cl_7F_6N_2O_5S$
Formula weight	973.86
Temperature/K	100.00(10)
Crystal system	monoclinic
Space group	$P2_1/n$
a/Å	13.1561(2)
b/Å	14.2737(2)
c/Å	21.6803(3)
α/	90
β/	90.6680(10)
γ/	90
Volume/Å^3	4070.98(10)
Z	4
ρ_{calc}g/cm^3	1.5888
μ/mm^{-1}	5.586
F(000)	1985.6
Crystal size/mm^3	0.3 × 0.1 × 0.1
Radiation	Cu Kα (λ = 1.54184)
2Θ range for data collection/°	7.42 to 152.9
Index ranges	$-16 \le h \le 16, -18 \le k \le 17, -26 \le l \le 21$
Reflections collected	49479
Independent reflections	8371 [R_{int} = 0.0454, R_{sigma} = 0.0283]
Data/restraints/parameters	8371/0/524
Goodness-of-fit on F^2	1.037
Final R indexes [I>=2σ (I)]	R_1 = 0.1082, wR_2 = 0.2794
Final R indexes [all data]	R_1 = 0.1136, wR_2 = 0.2832
Largest diff. peak/hole / e Å^{-3}	2.31/-1.61

9. Copies of NMR spectra (3aa-3la and **3na-3pa** were obtained as an atropoisomer mixture, and only the C5-C6 *trans* product was observed; **3ma** no atropoisomers. **3qa-3qf** Only C5-C6 *cis* product was observed**)**

3aa, ^{1}H NMR (600 MHz, $CDCl_3$)

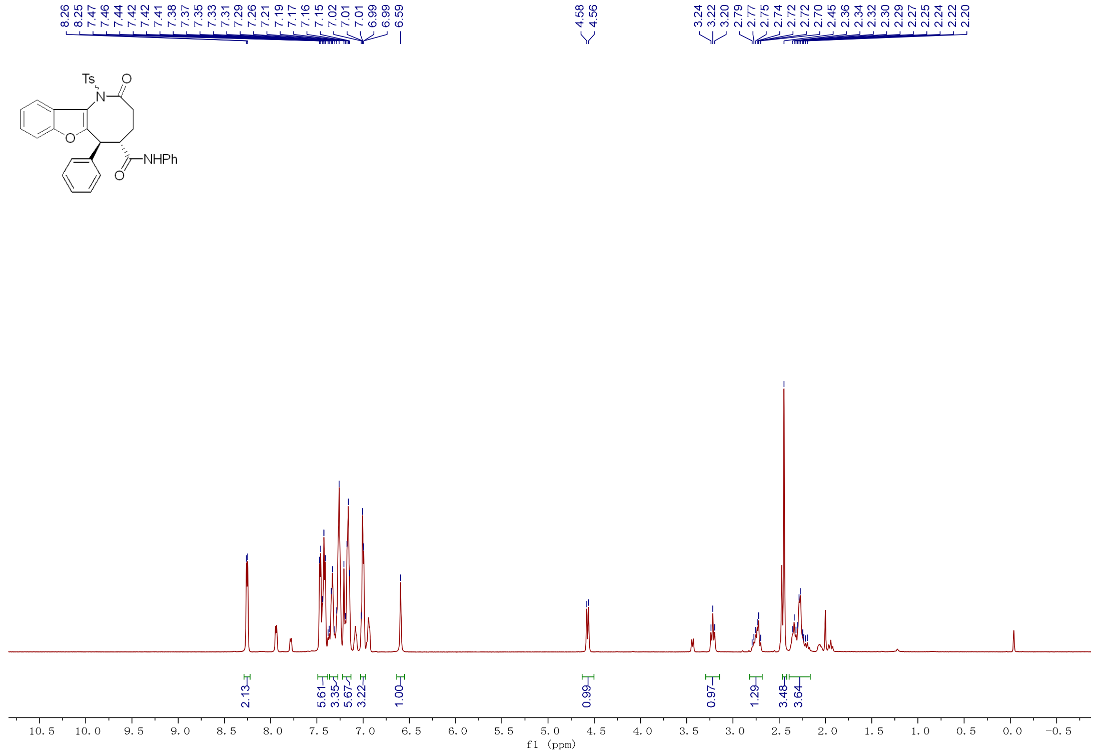

3aa, ^{13}C NMR (101 MHz, $CDCl_3$)

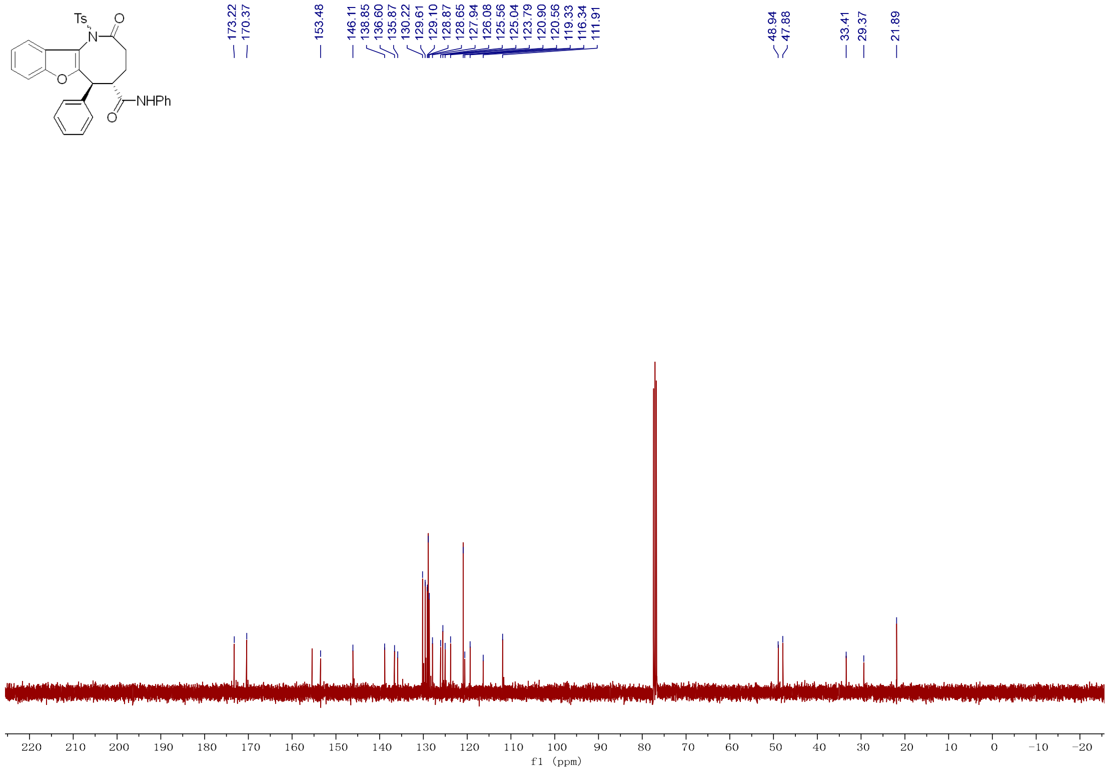

3ba, ^{1}H NMR (600 MHz, $CDCl_3$)

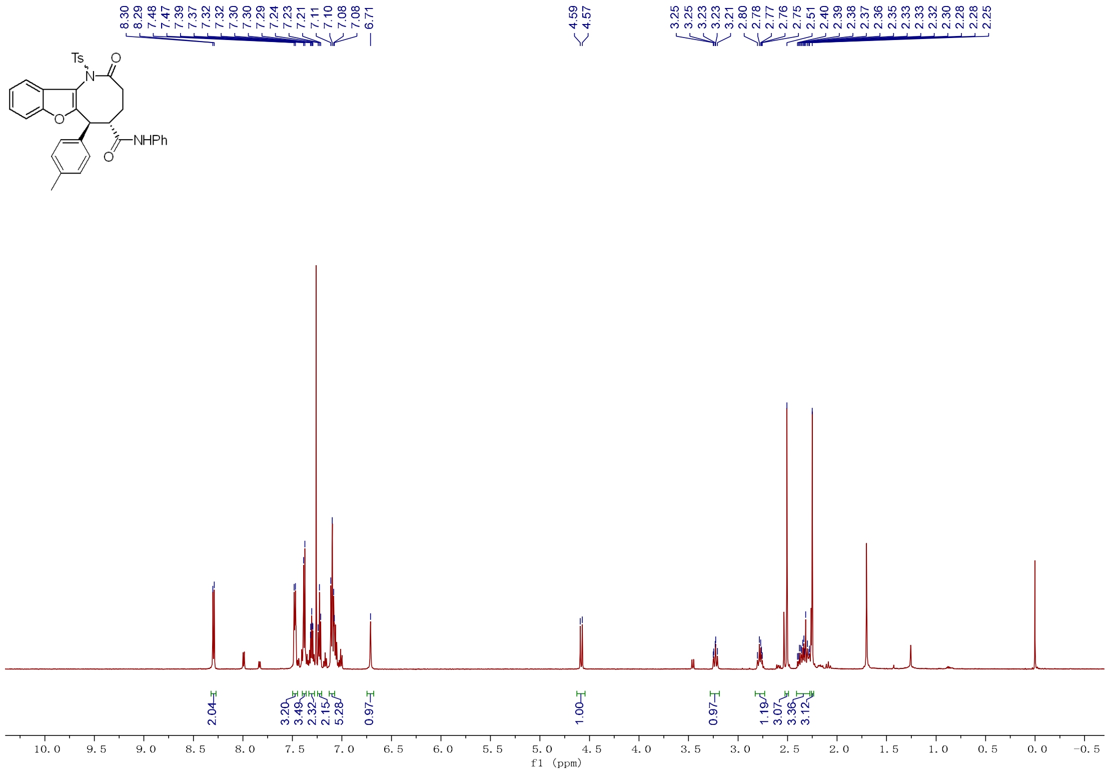

3ba, ^{13}C NMR (151 MHz, $CDCl_3$)

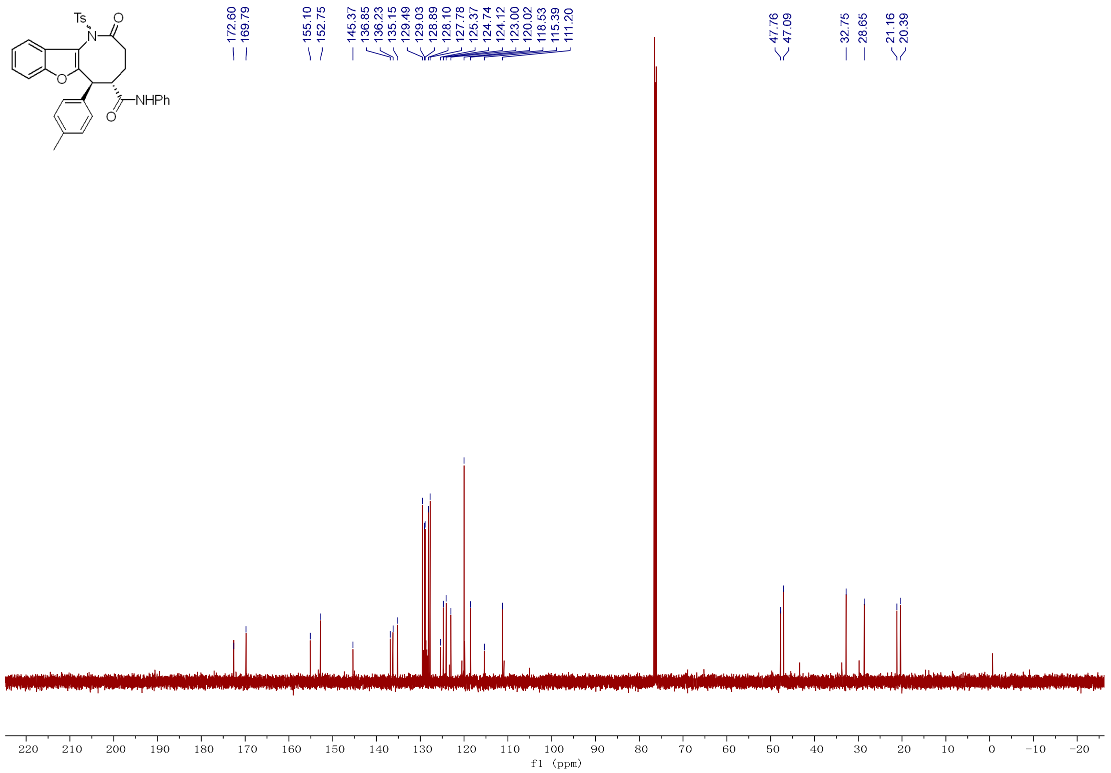

3ca, ^{1}H NMR (600 MHz, $CDCl_3$)

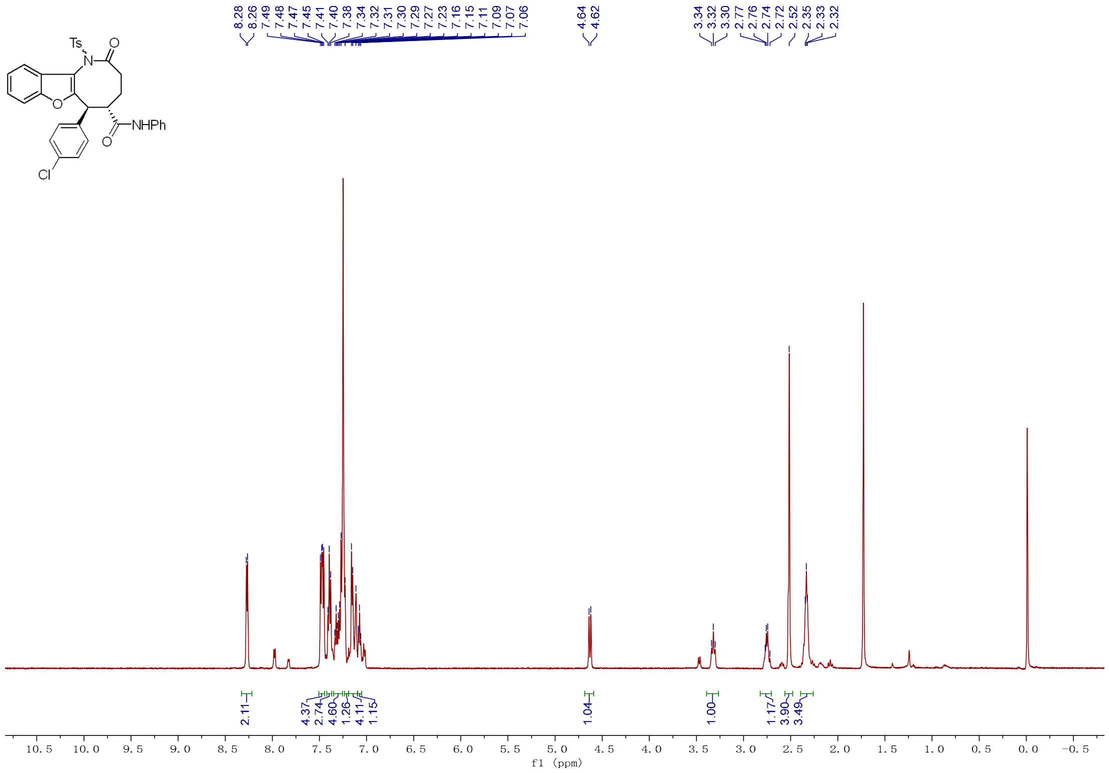

3ca, ^{13}C NMR (151 MHz, $CDCl_3$)

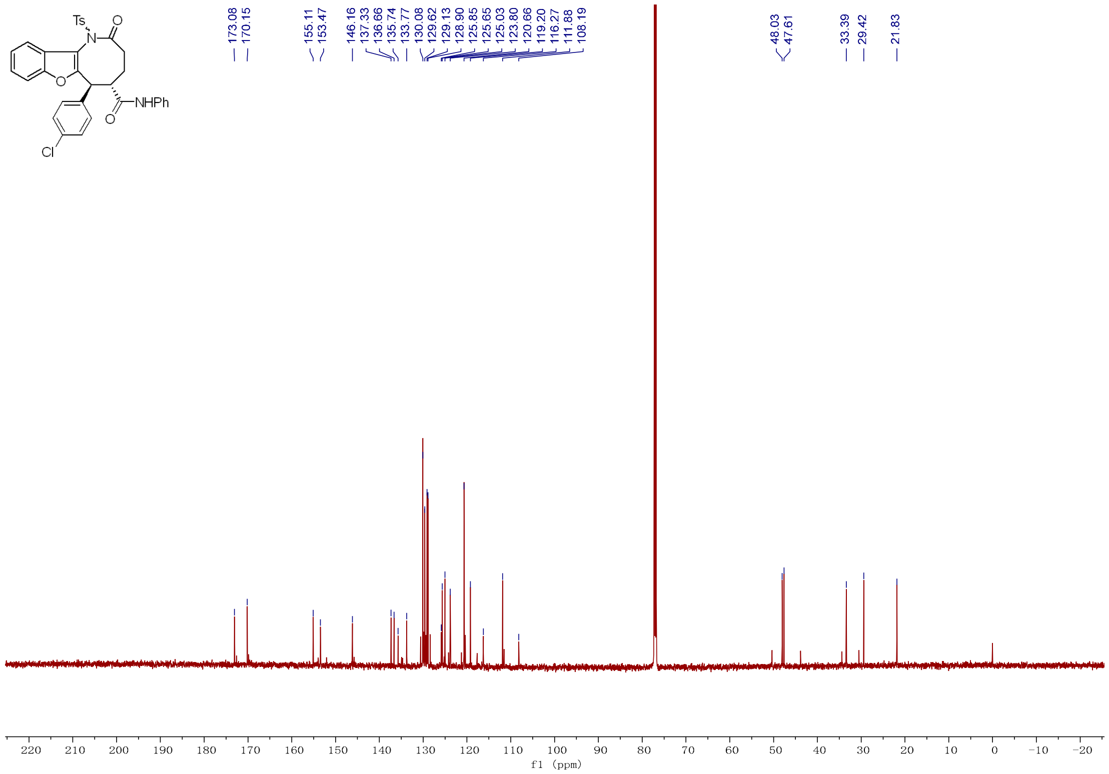

3da, ^{1}H NMR (600 MHz, $CDCl_3$)

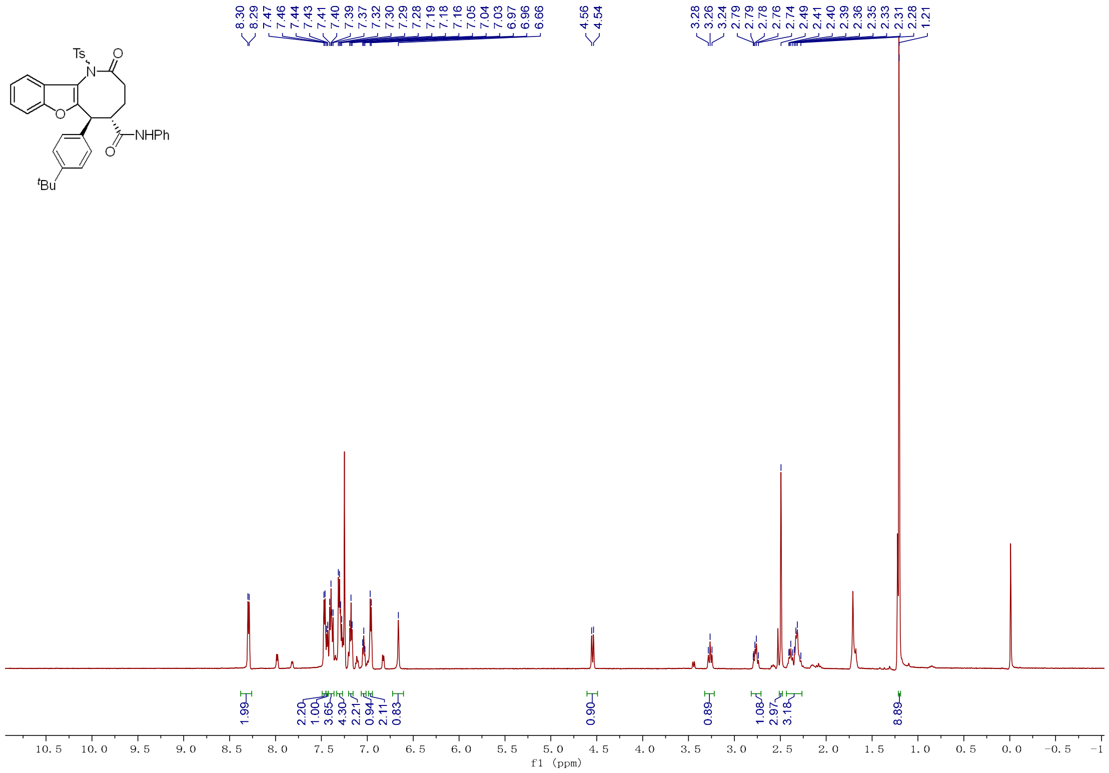

3da, ^{13}C NMR (101 MHz, $CDCl_3$)

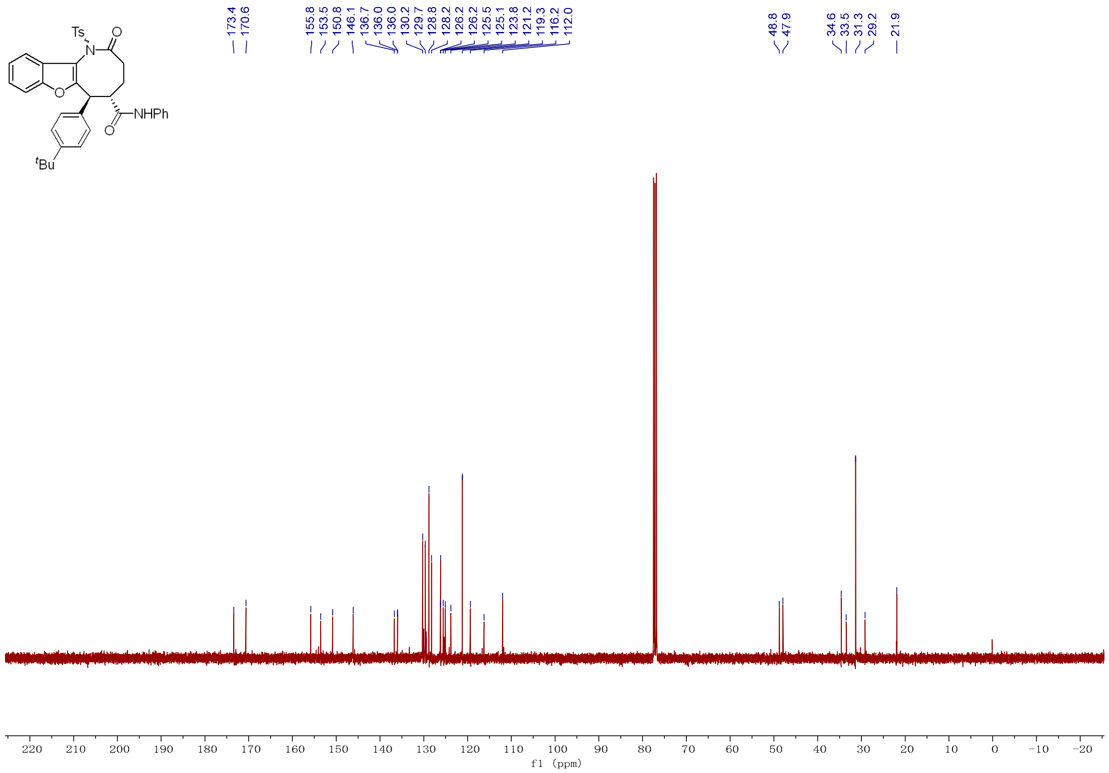

3ea, ^{1}H NMR (600 MHz, $CDCl_3$)

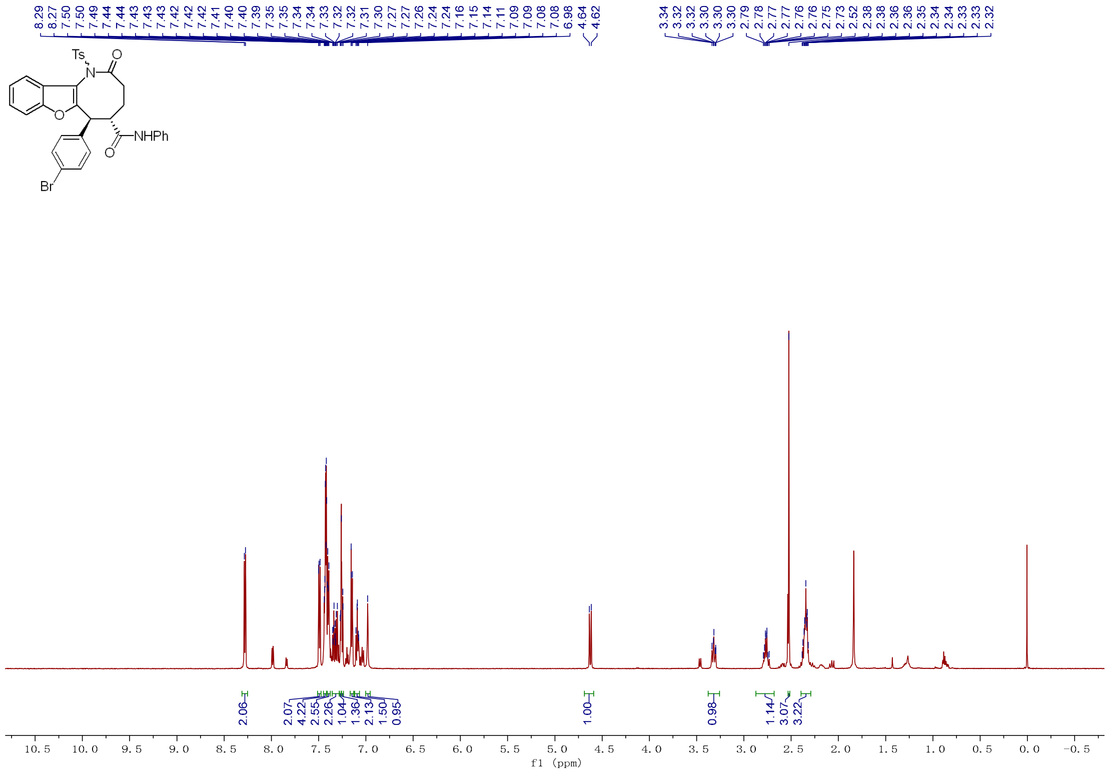

3ea, ^{13}C NMR (151 MHz, $CDCl_3$)

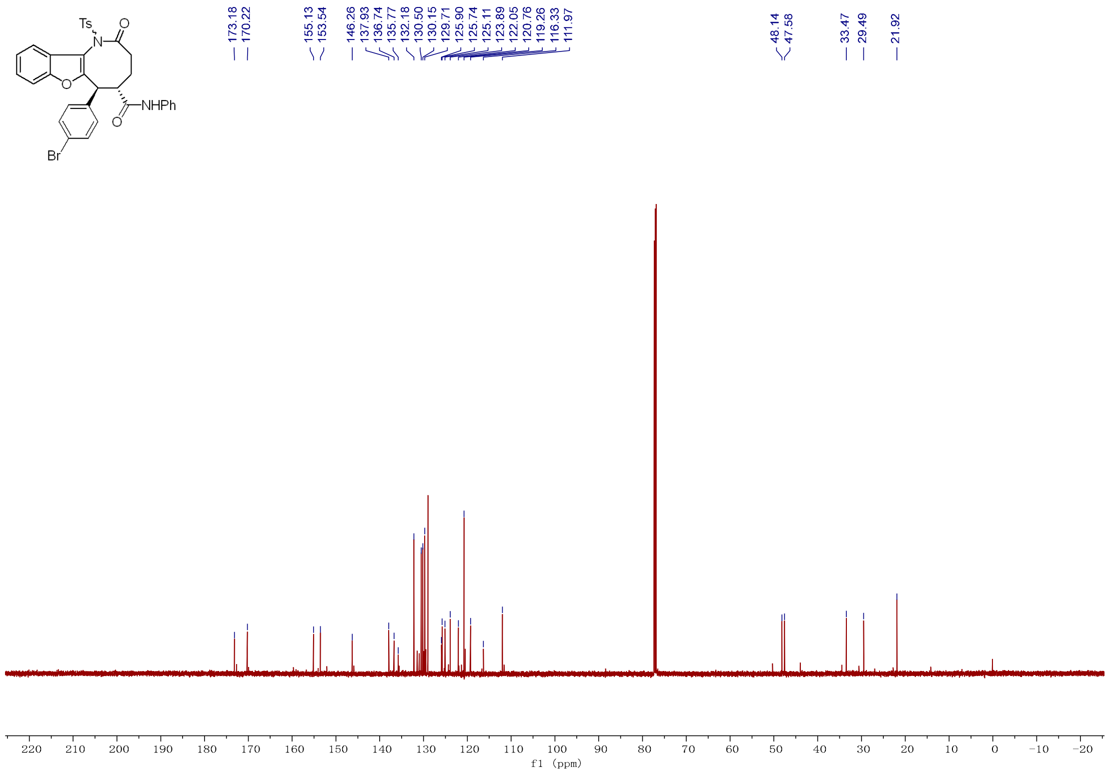

3fa, ^{1}H NMR (400 MHz, $CDCl_3$)

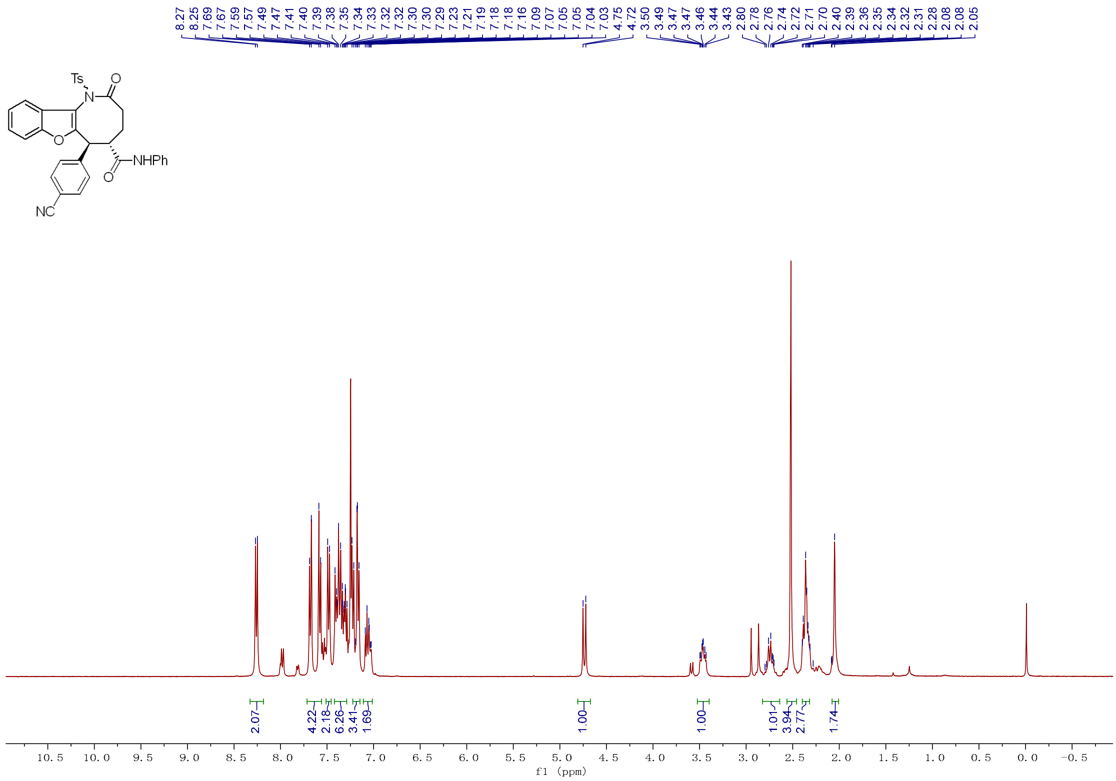

3fa, ^{13}C NMR (151 MHz, $CDCl_3$)

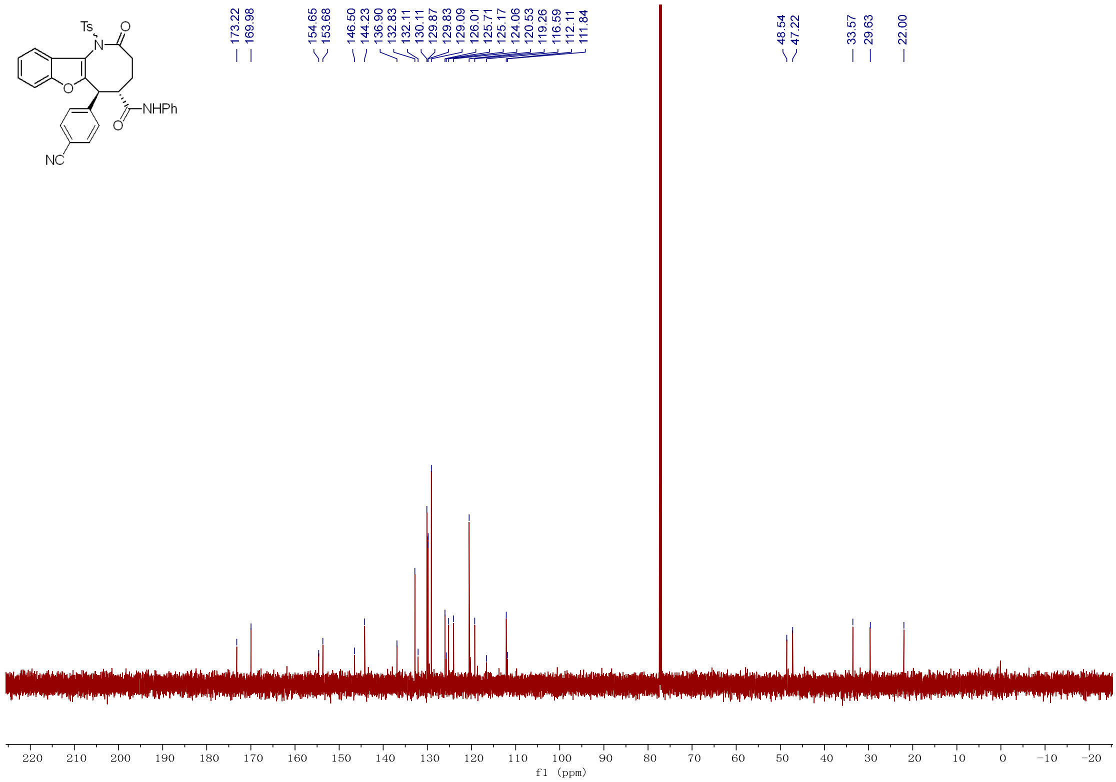

3ga, ^{1}H NMR (600 MHz, $CDCl_3$)

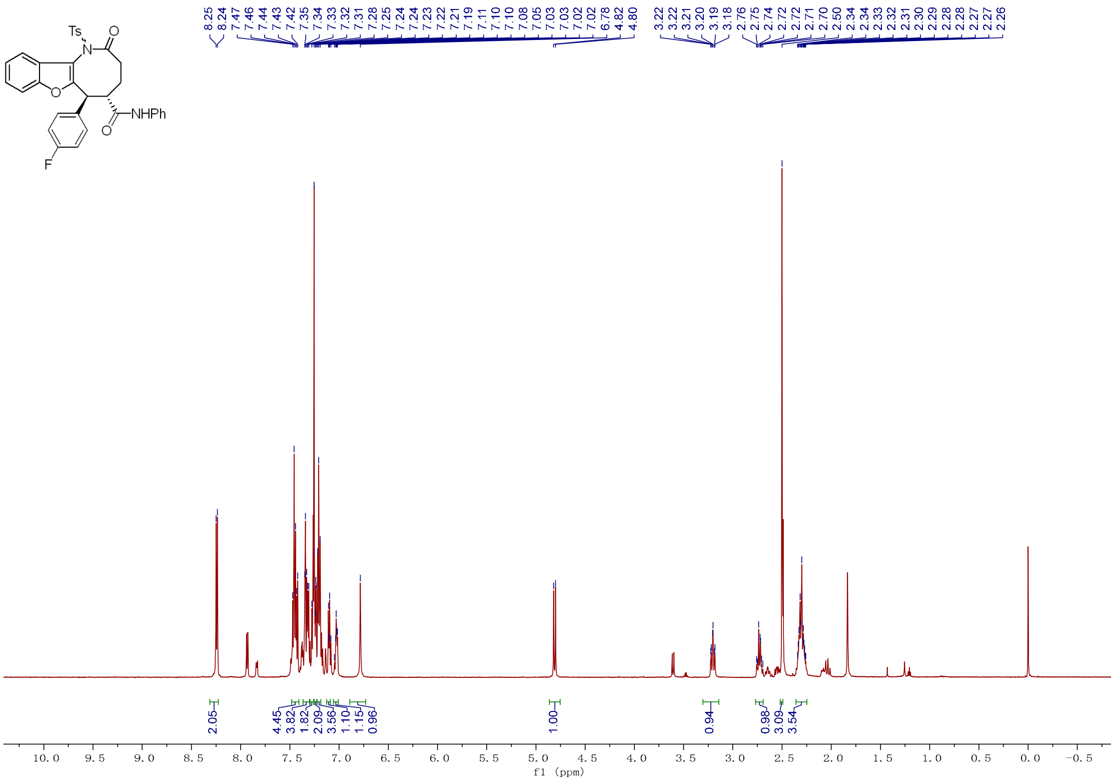

3ga, ^{13}C NMR (101 MHz, $CDCl_3$)

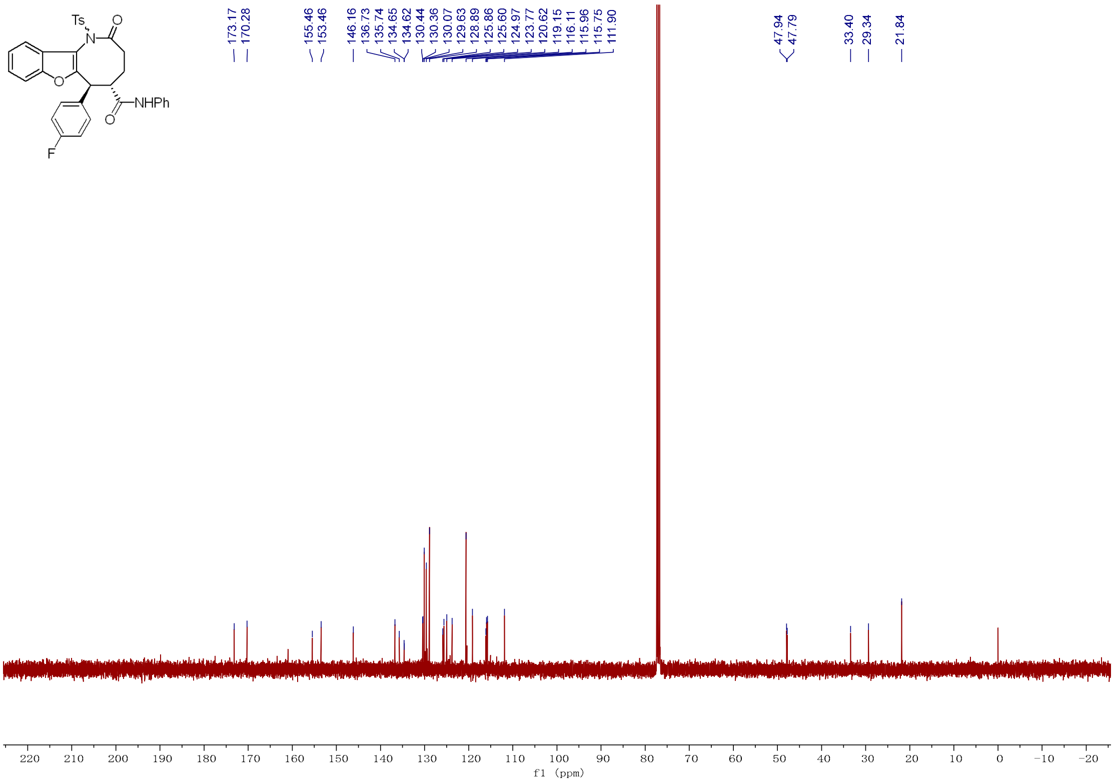

3ha, ^{1}H NMR (400 MHz, $CDCl_3$)

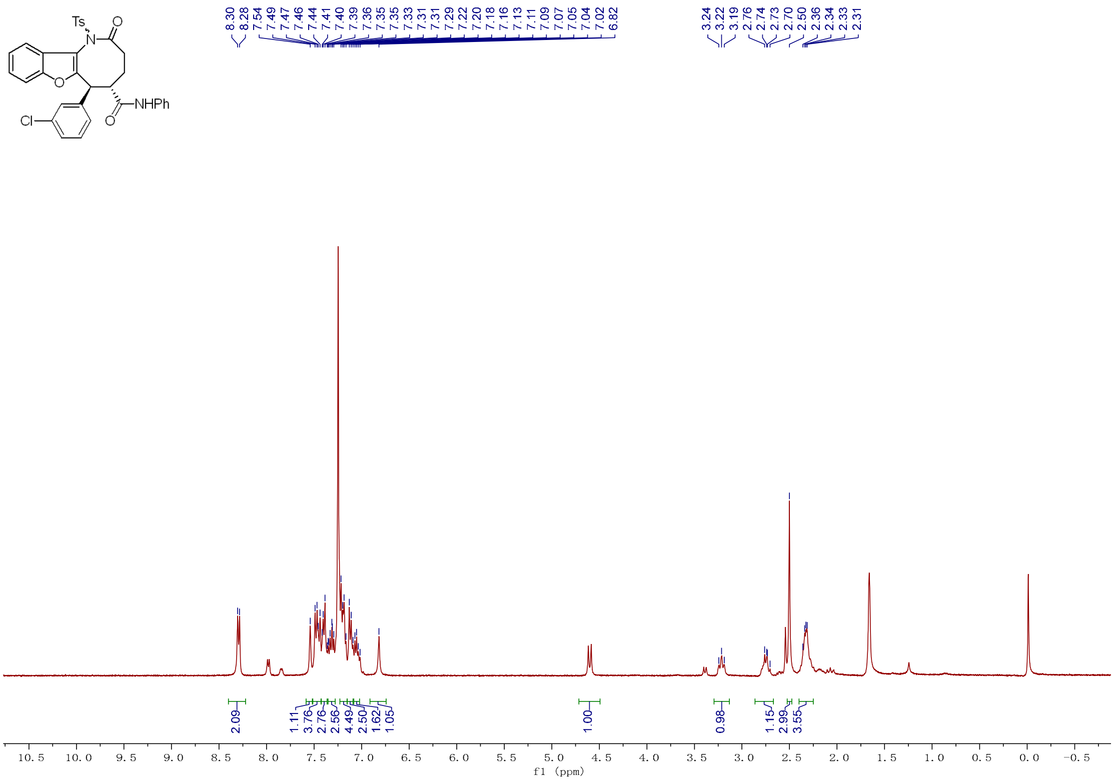

3ha, ^{13}C NMR (151 MHz, $CDCl_3$)

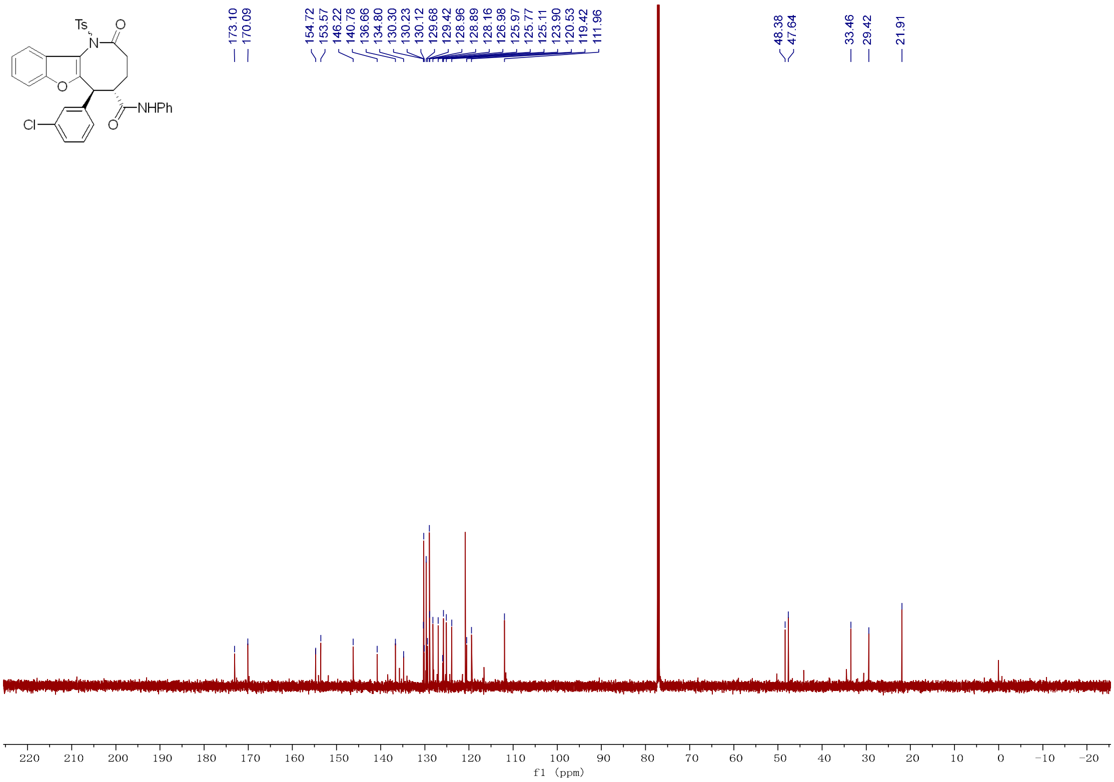

3ia, ^{1}H NMR (400 MHz, $CDCl_3$)

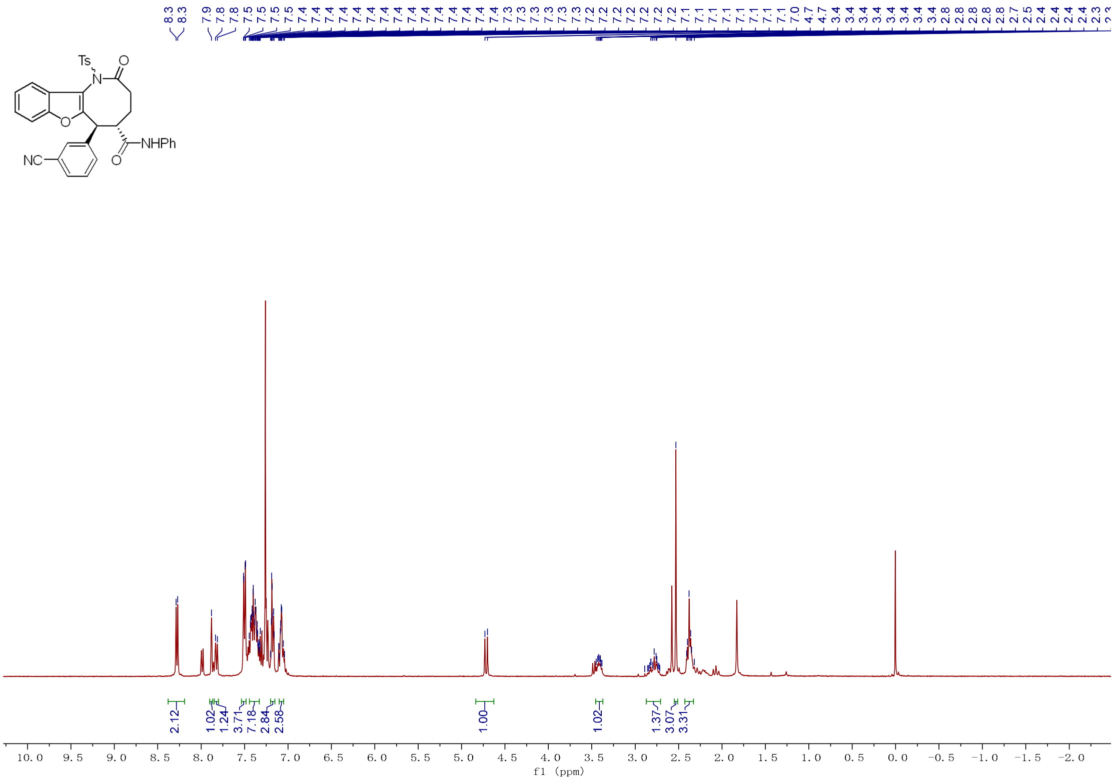

3ia, ^{13}C NMR (101 MHz, $CDCl_3$)

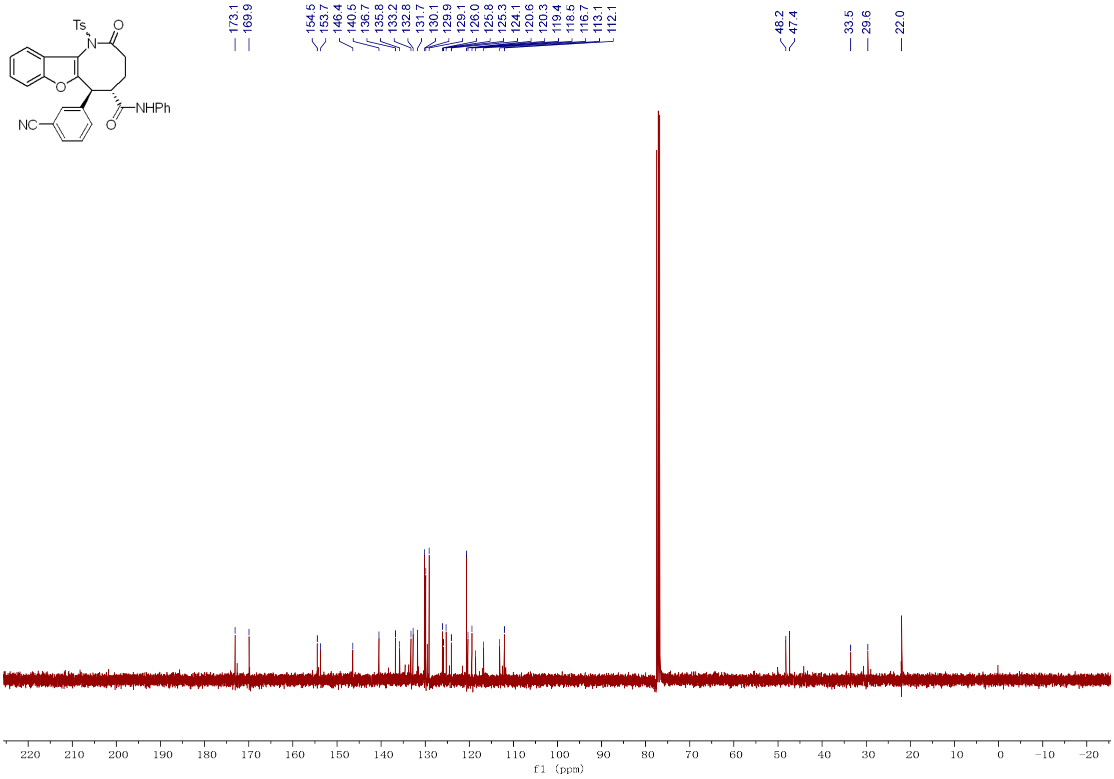

3ja, ^{1}H NMR (400 MHz, $CDCl_3$)

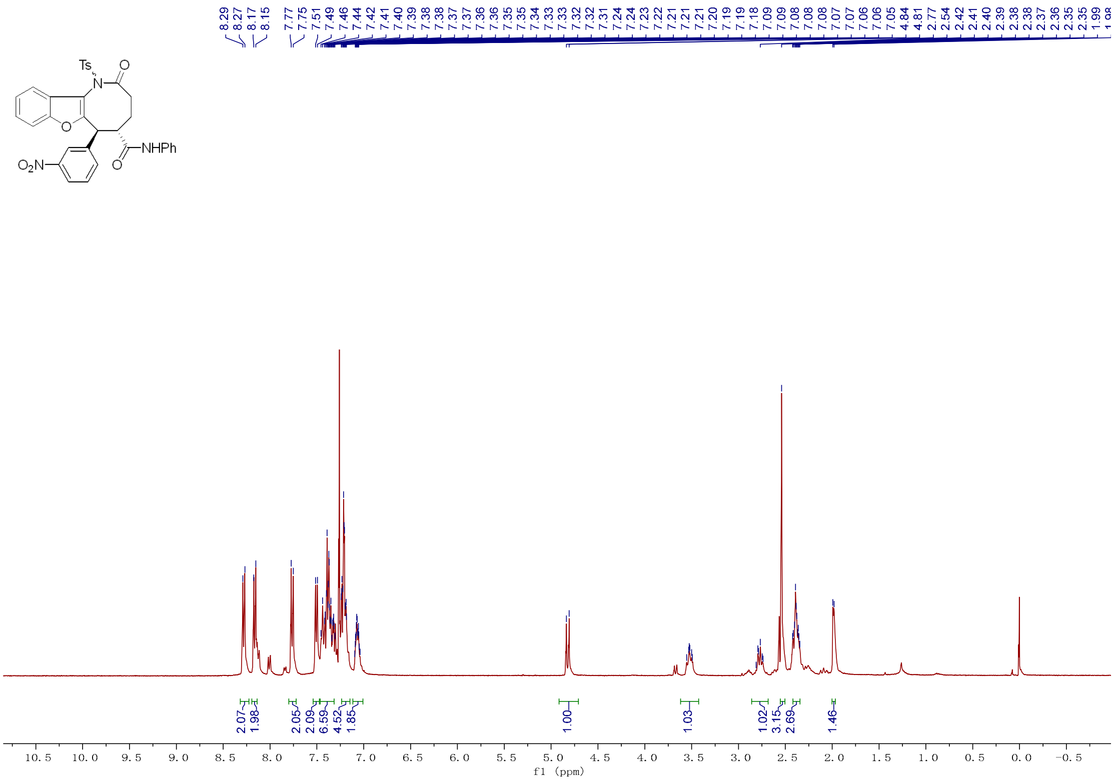

3ja, ^{13}C NMR (151 MHz, $CDCl_3$)

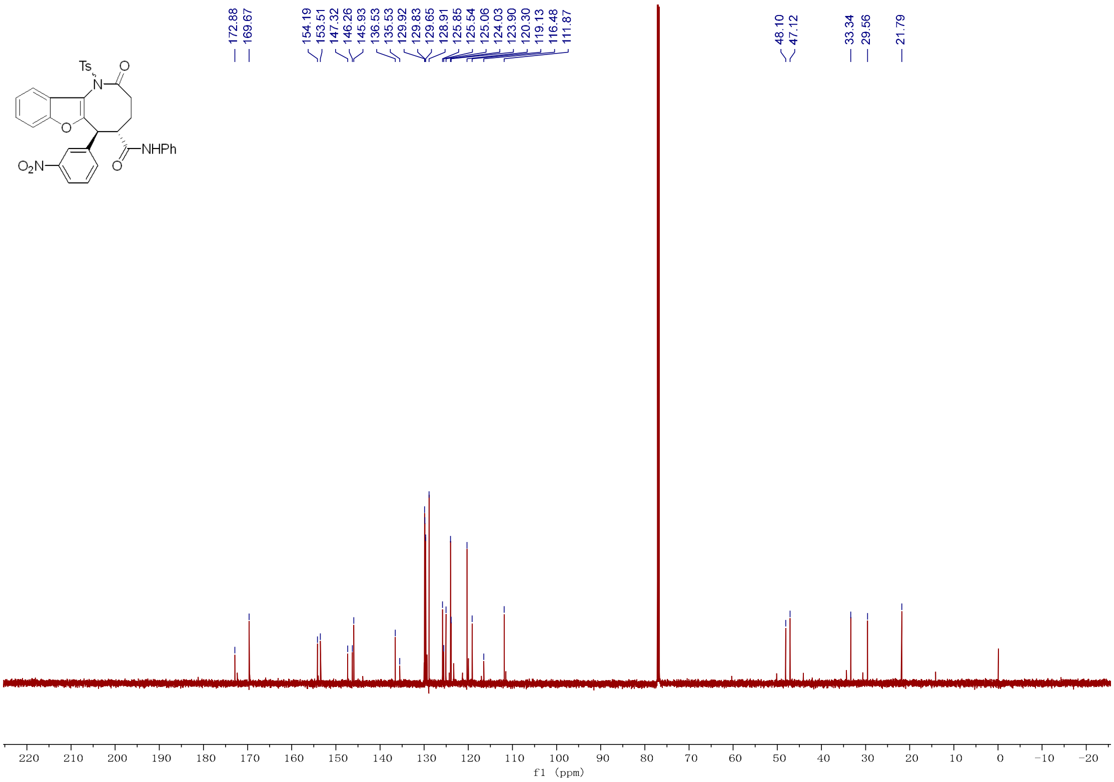

3ka, ^{1}H NMR (600 MHz, CDCl$_3$)

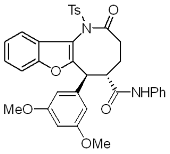

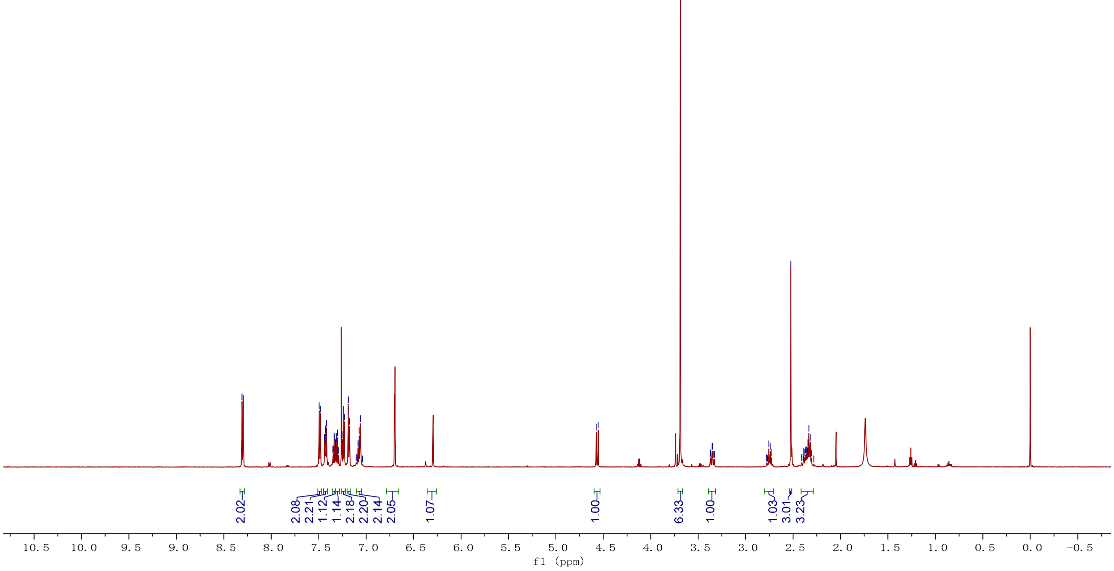

3ka, ^{13}C NMR (101 MHz, CDCl$_3$)

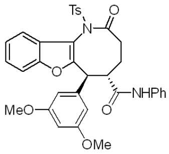

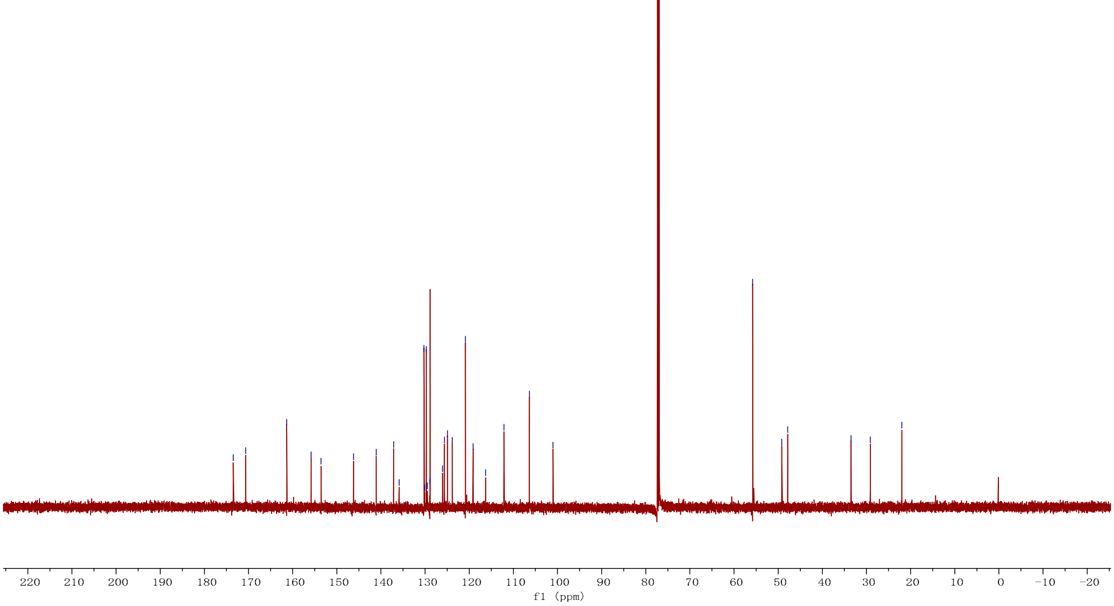

3la, ¹H NMR (400 MHz, CDCl₃)

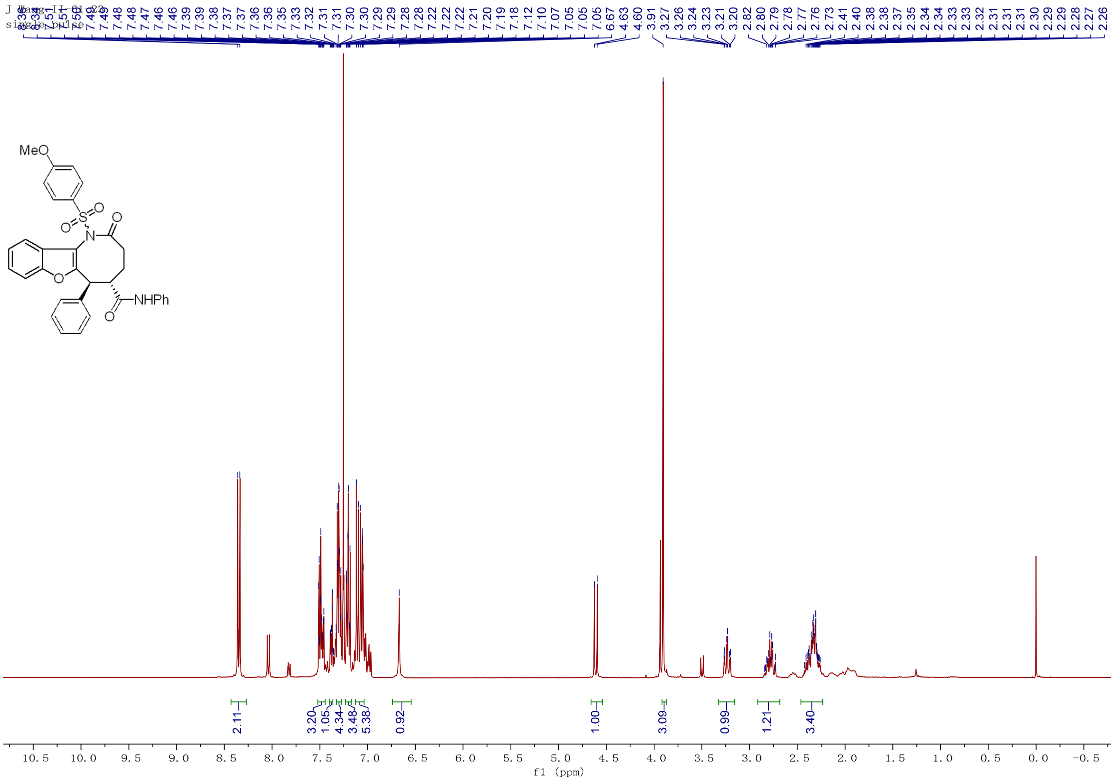

3la, ¹³C NMR (101 MHz, CDCl₃)

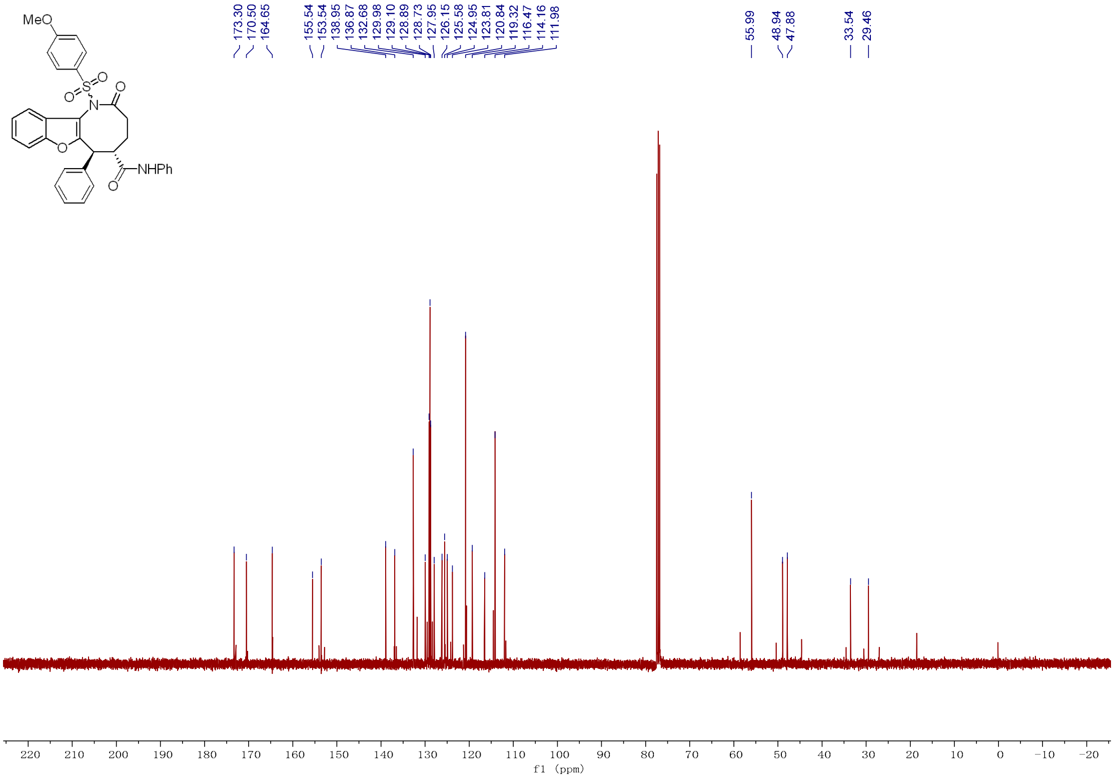

3ma, ¹H NMR (400 MHz, $CDCl_3$)

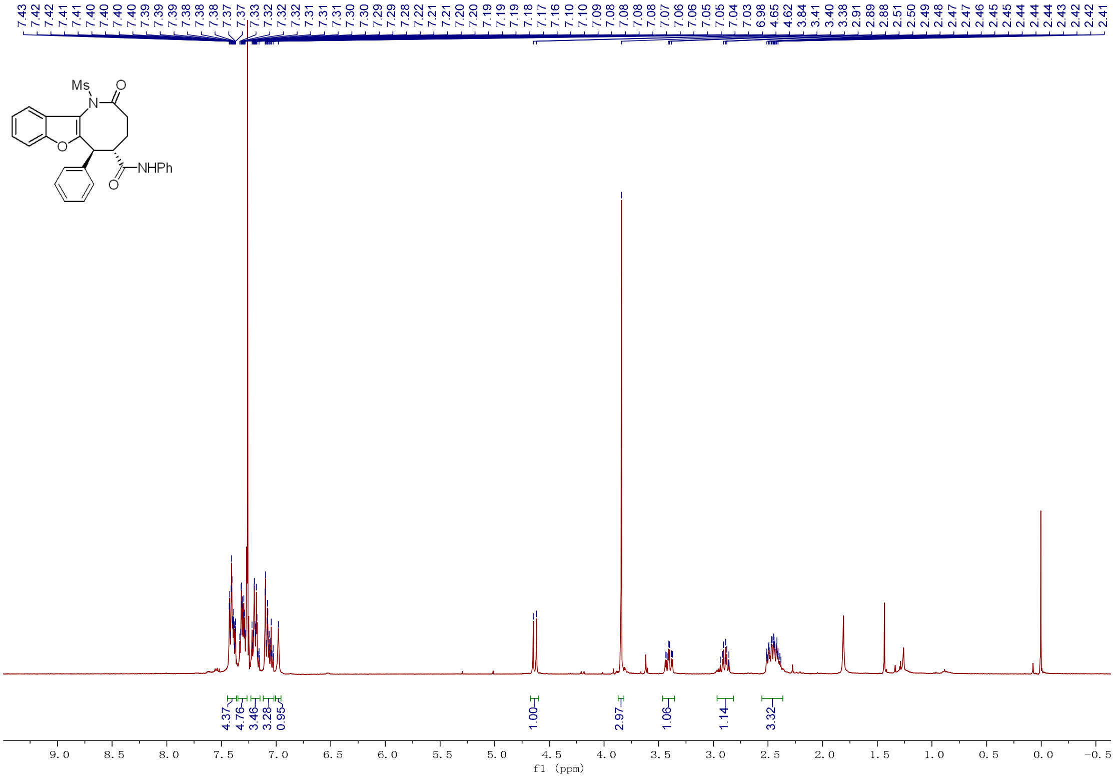

3ma, ¹³C NMR (151 MHz, $CDCl_3$)

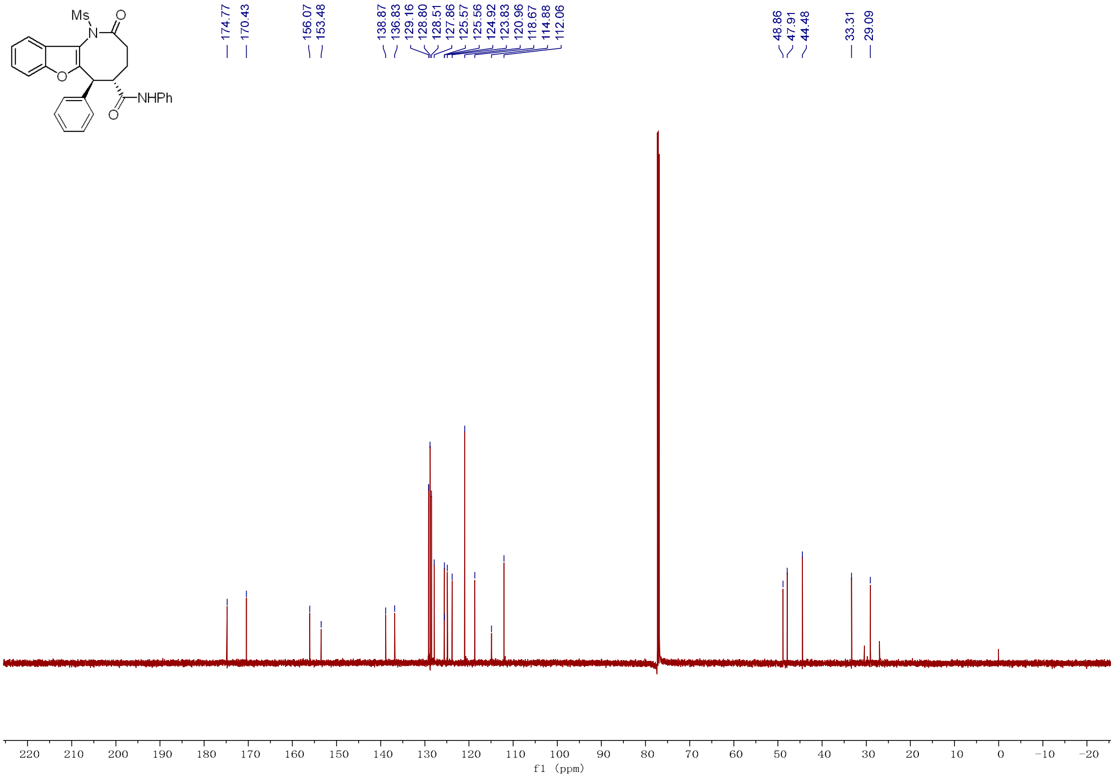

3na, ¹H NMR (400 MHz, CDCl$_3$)

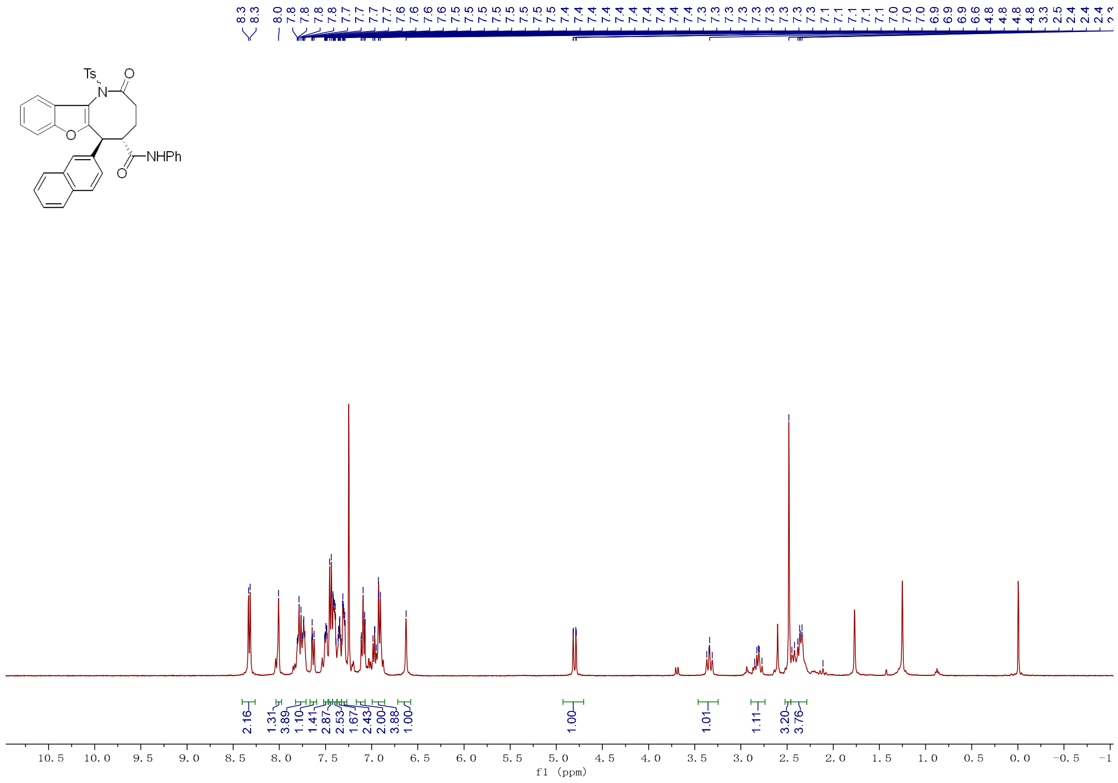

3na, ¹³C NMR (101 MHz, CDCl$_3$)

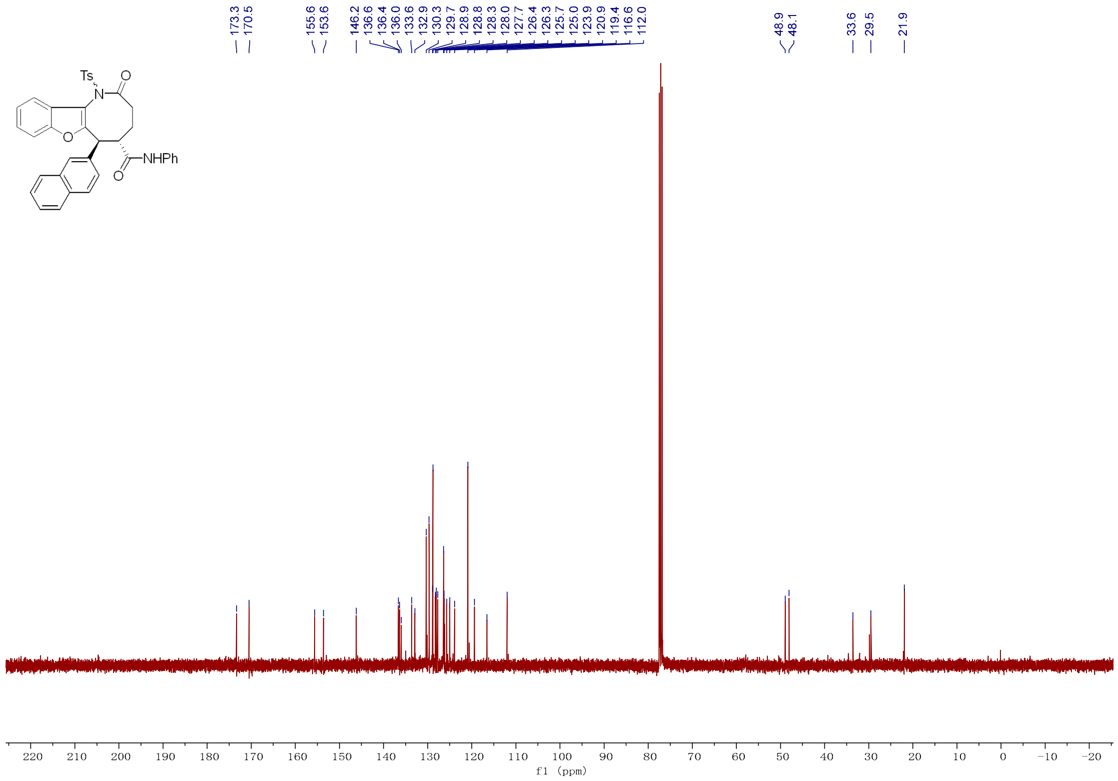

3oa, ^{1}H NMR (600 MHz, $CDCl_3$)

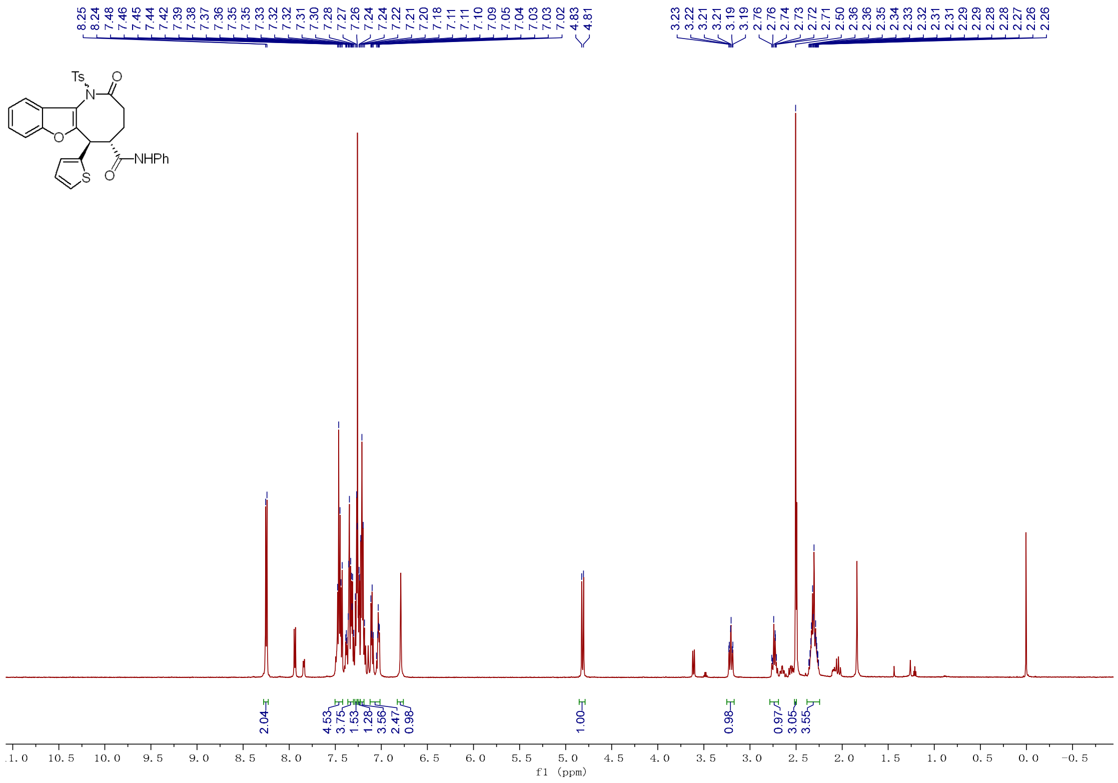

3oa, ^{13}C NMR (151 MHz, $CDCl_3$)

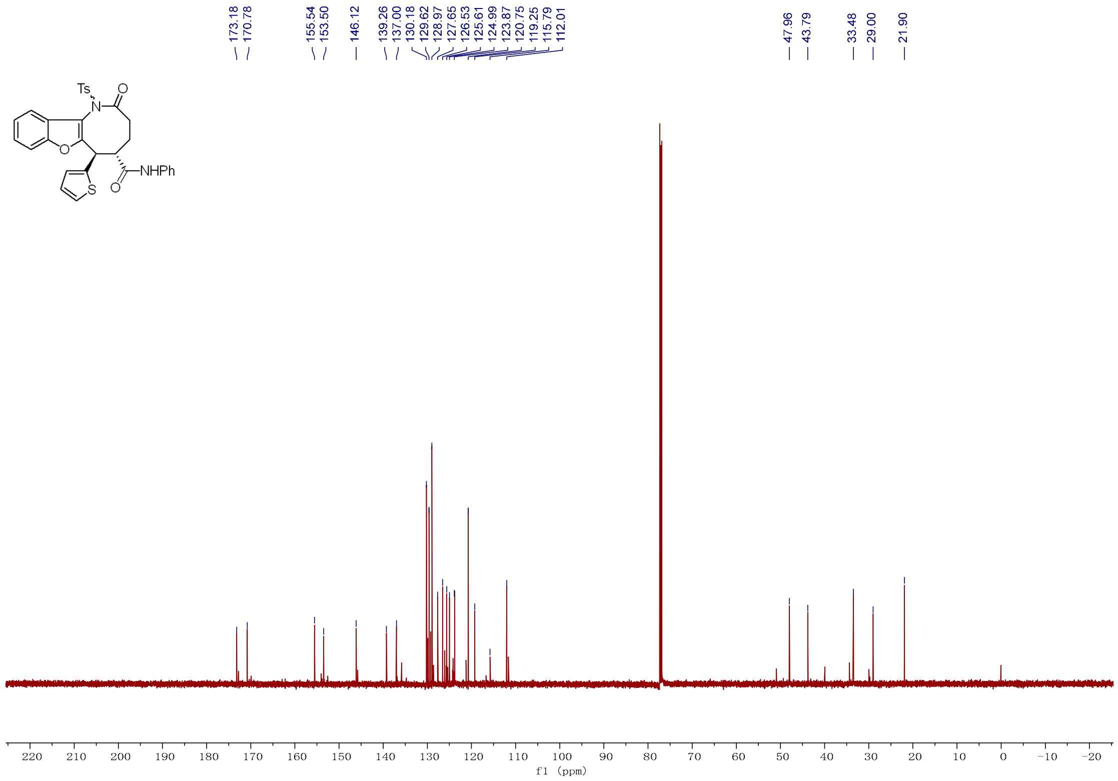

3pa, ^{1}H NMR (400 MHz, $CDCl_3$)

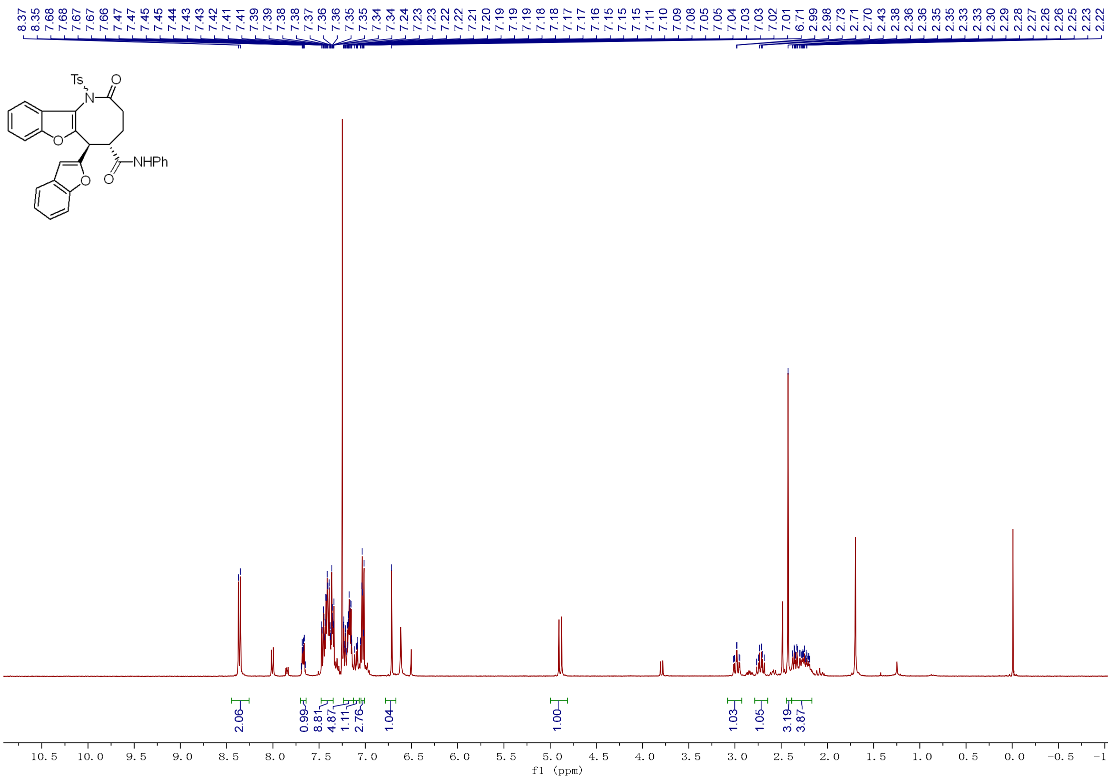

3pa, ^{13}C NMR (101 MHz, $CDCl_3$)

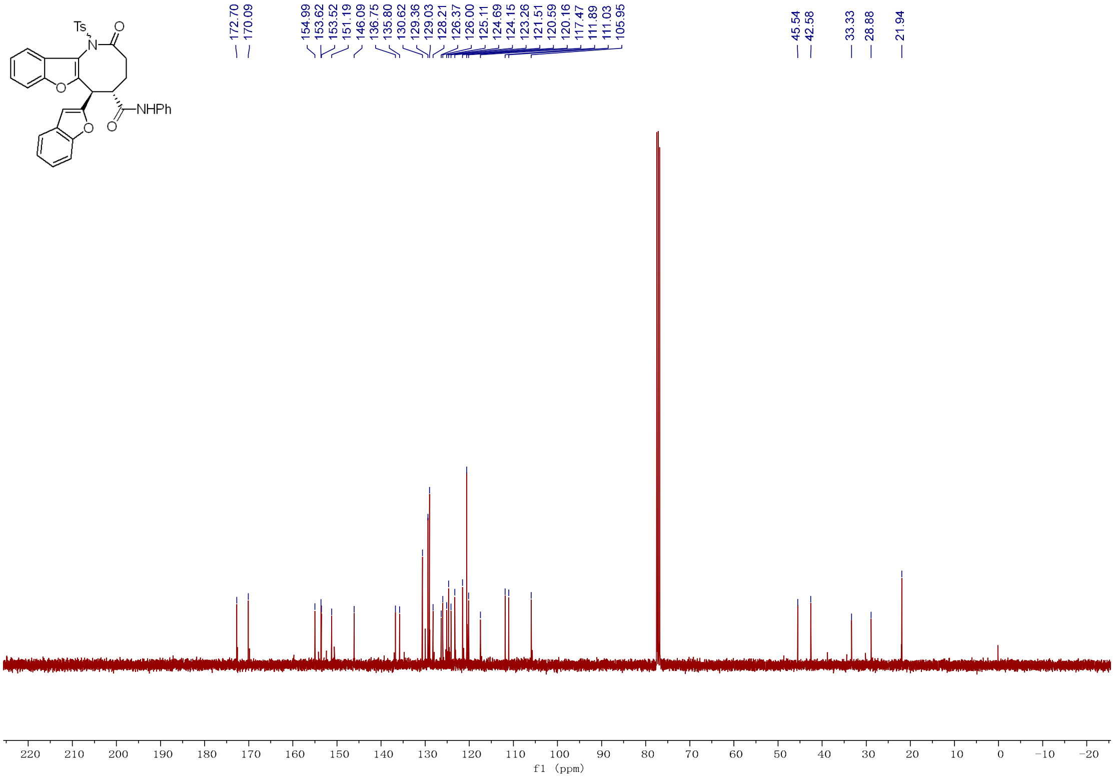

3qa, ¹H NMR (400 MHz, $CDCl_3$)

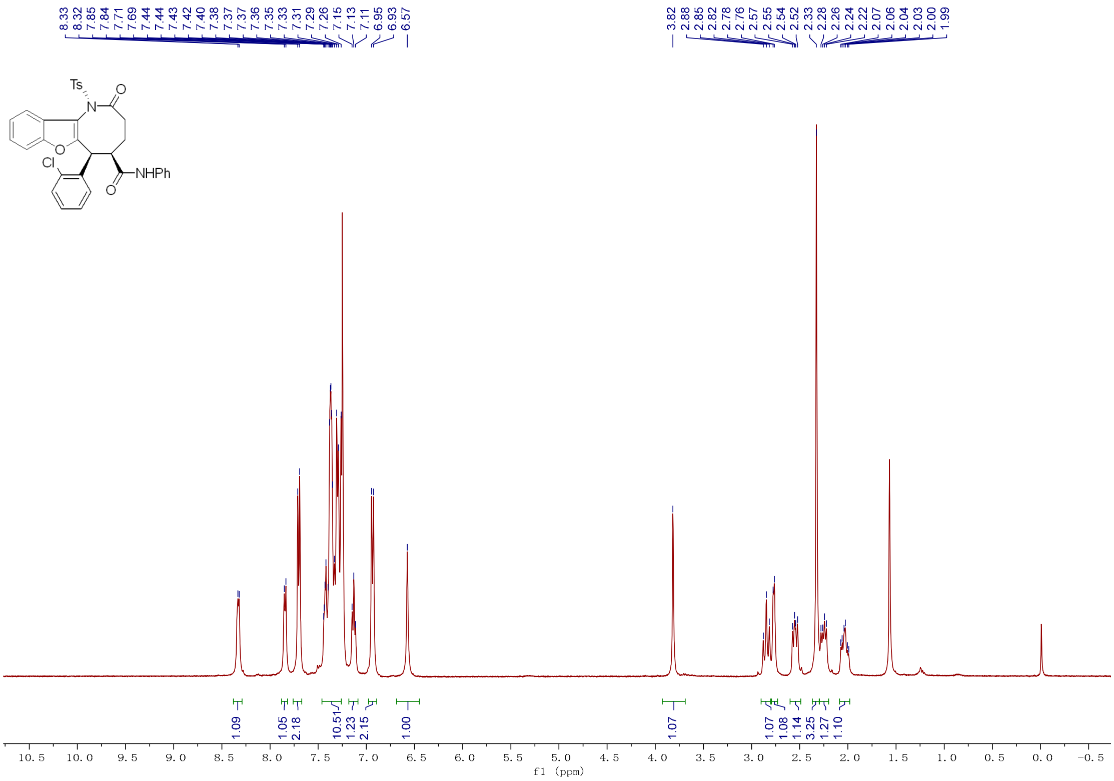

3qa, ¹³C NMR (101 MHz, $CDCl_3$)

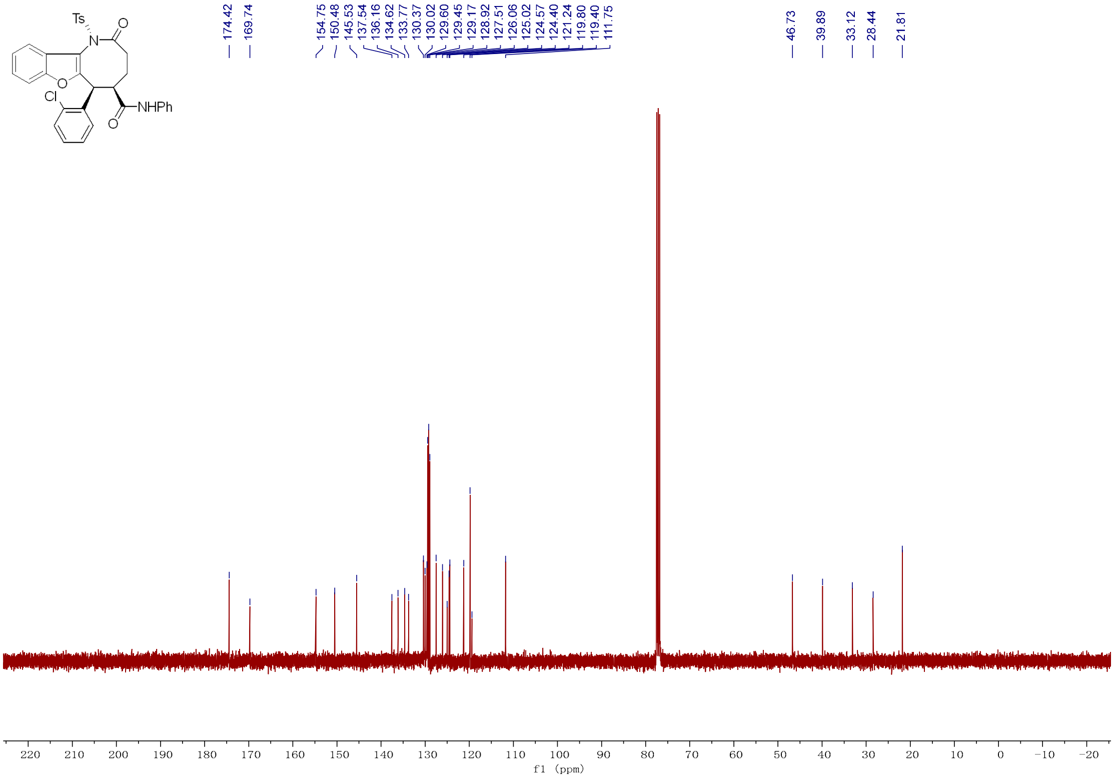

3qb, ^{1}H NMR (600 MHz, $CDCl_3$)

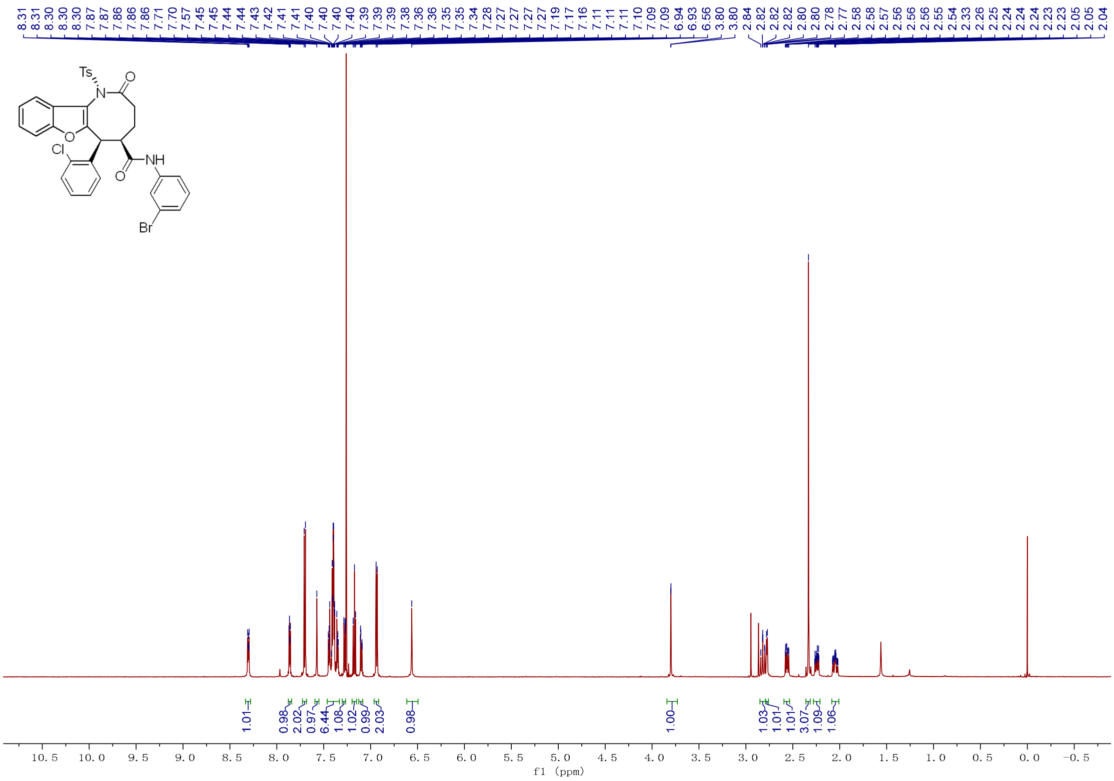

3qb, ^{13}C NMR (151 MHz, $CDCl_3$)

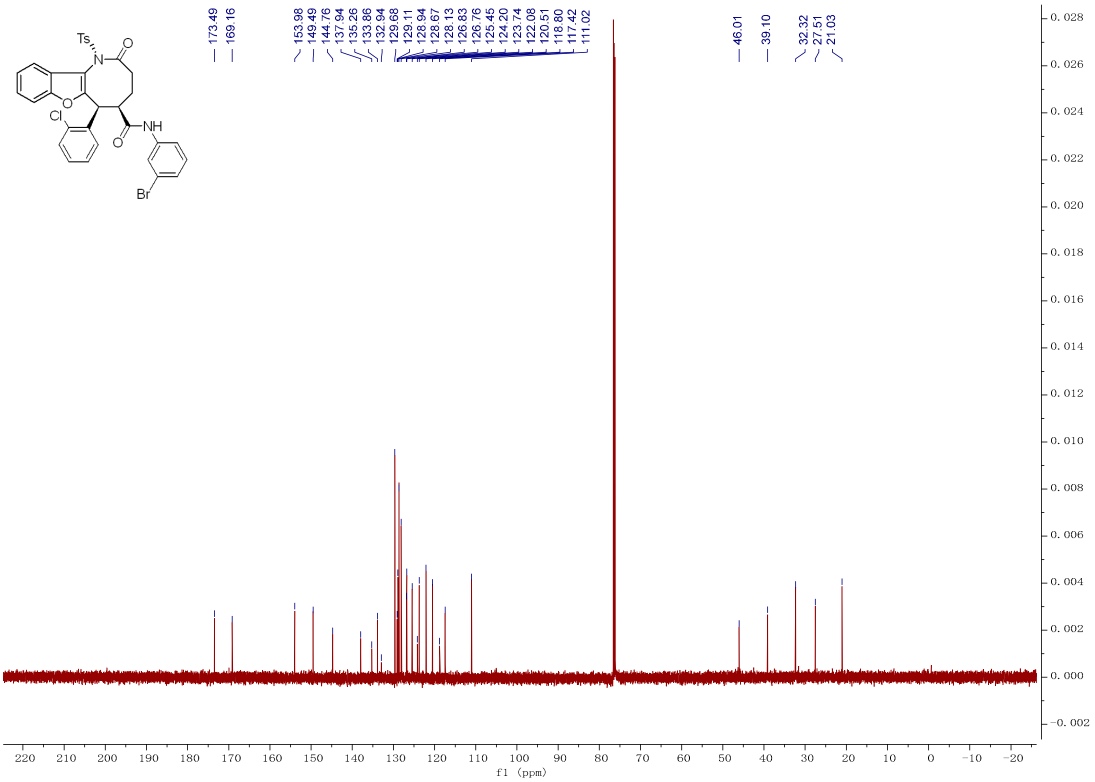

3qc, ^{1}H NMR (400 MHz, $CDCl_3$)

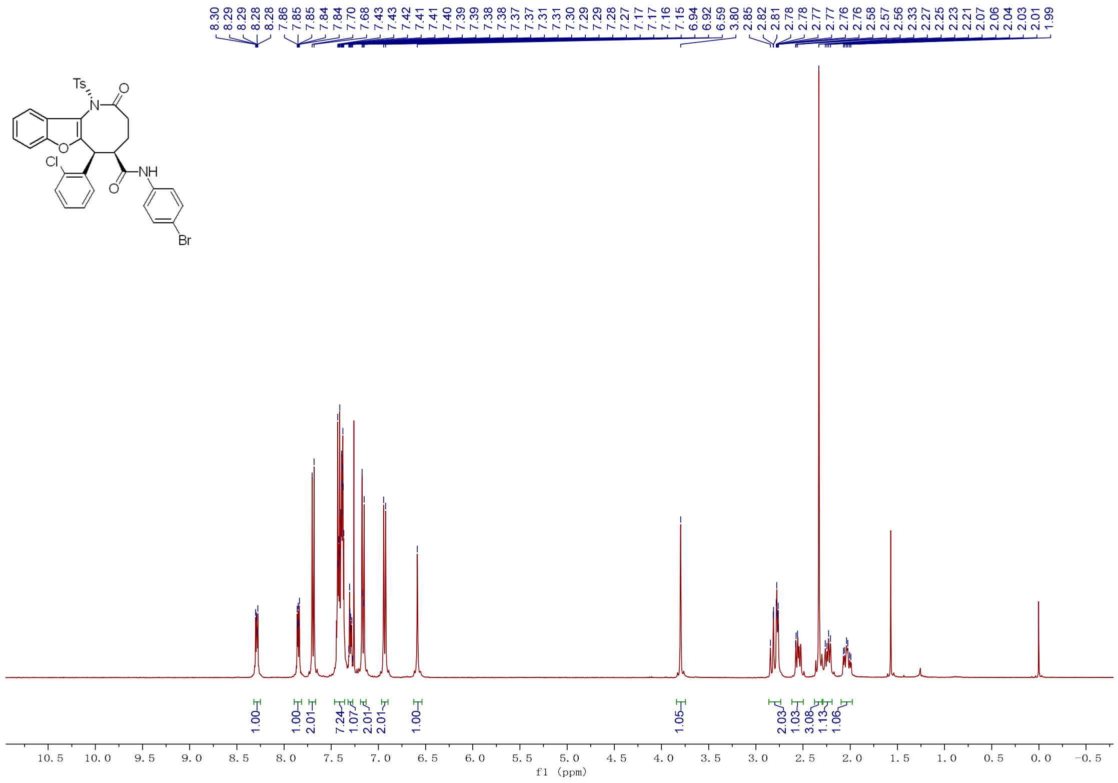

3qc, ^{13}C NMR (101 MHz, $CDCl_3$)

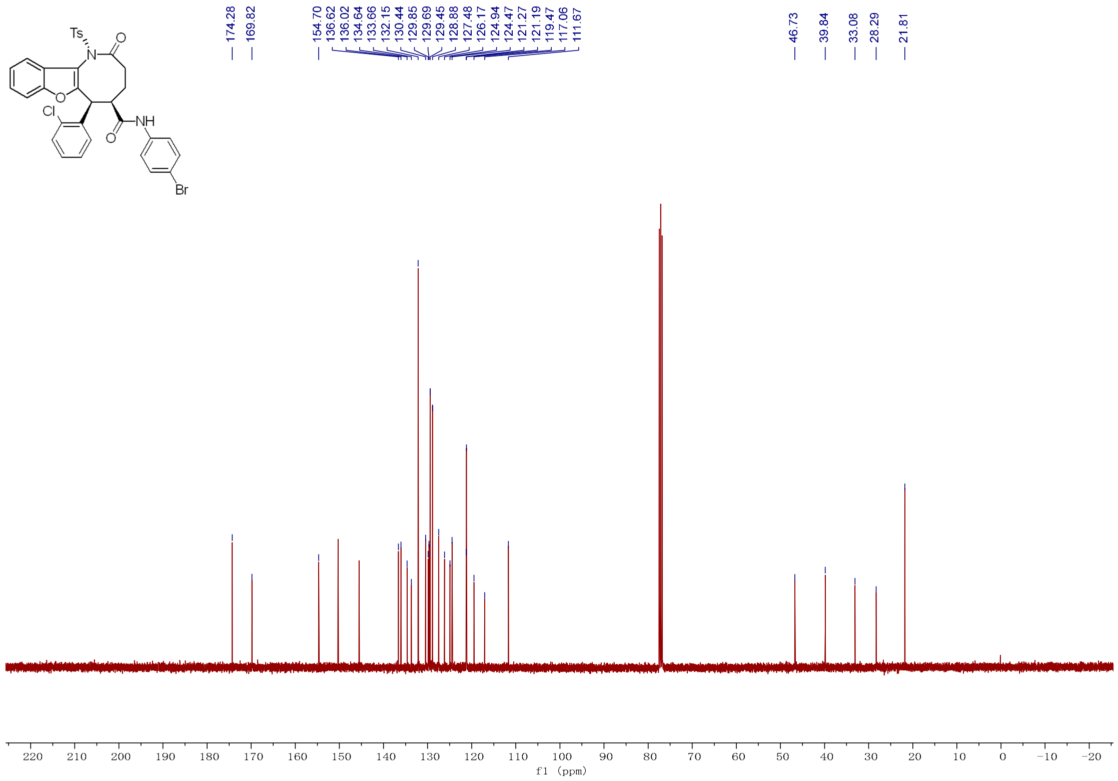

3qd, ^{1}H NMR (600 MHz, $CDCl_3$)

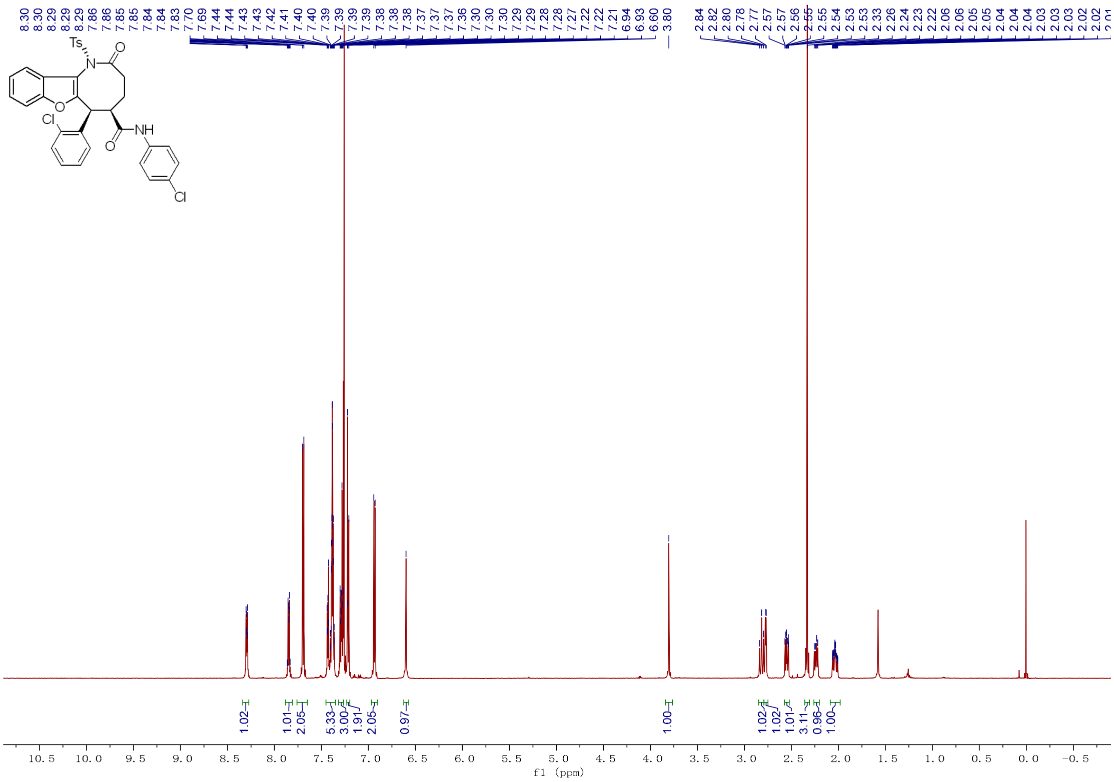

3qd, ^{13}C NMR (151 MHz, $CDCl_3$)

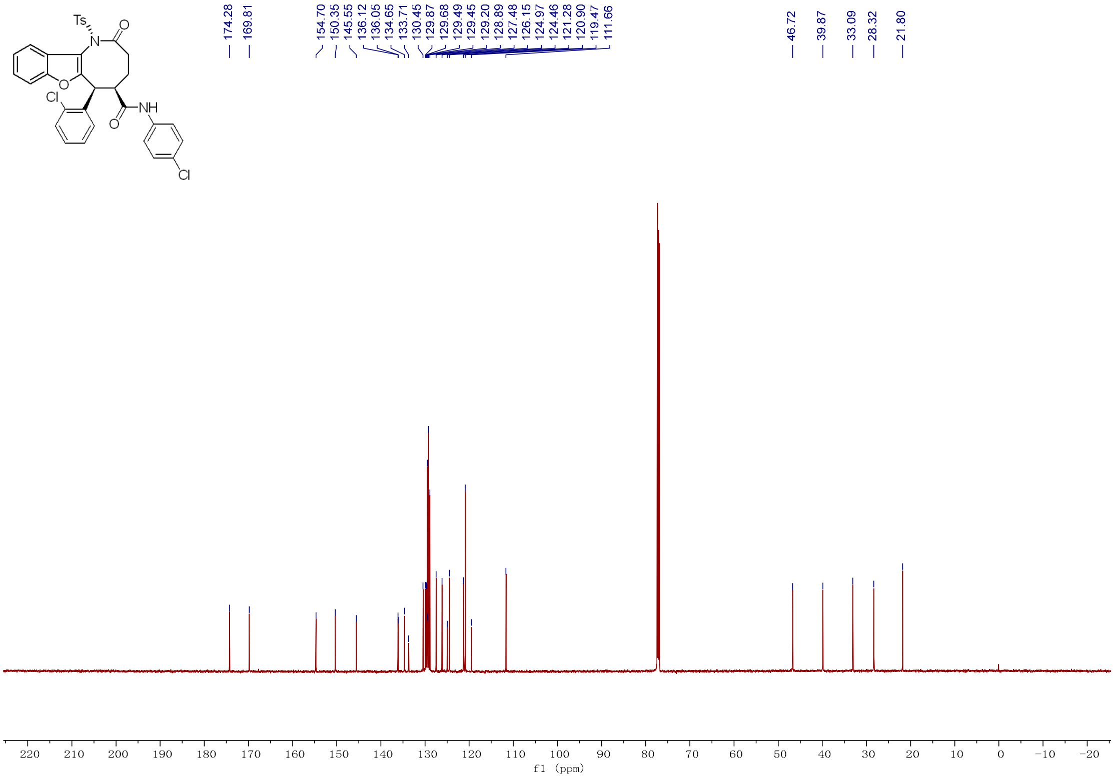

3qe, ^{1}H NMR (400 MHz, $CDCl_3$)

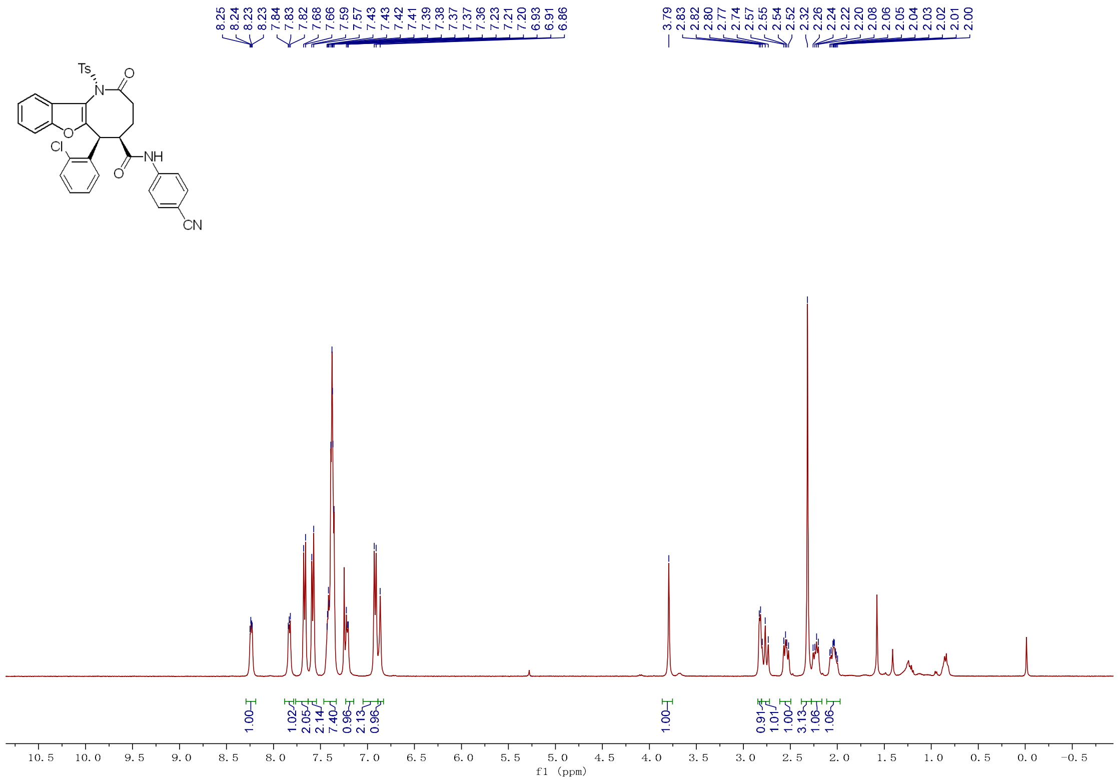

3qe, ^{13}C NMR (101 MHz, $CDCl_3$)

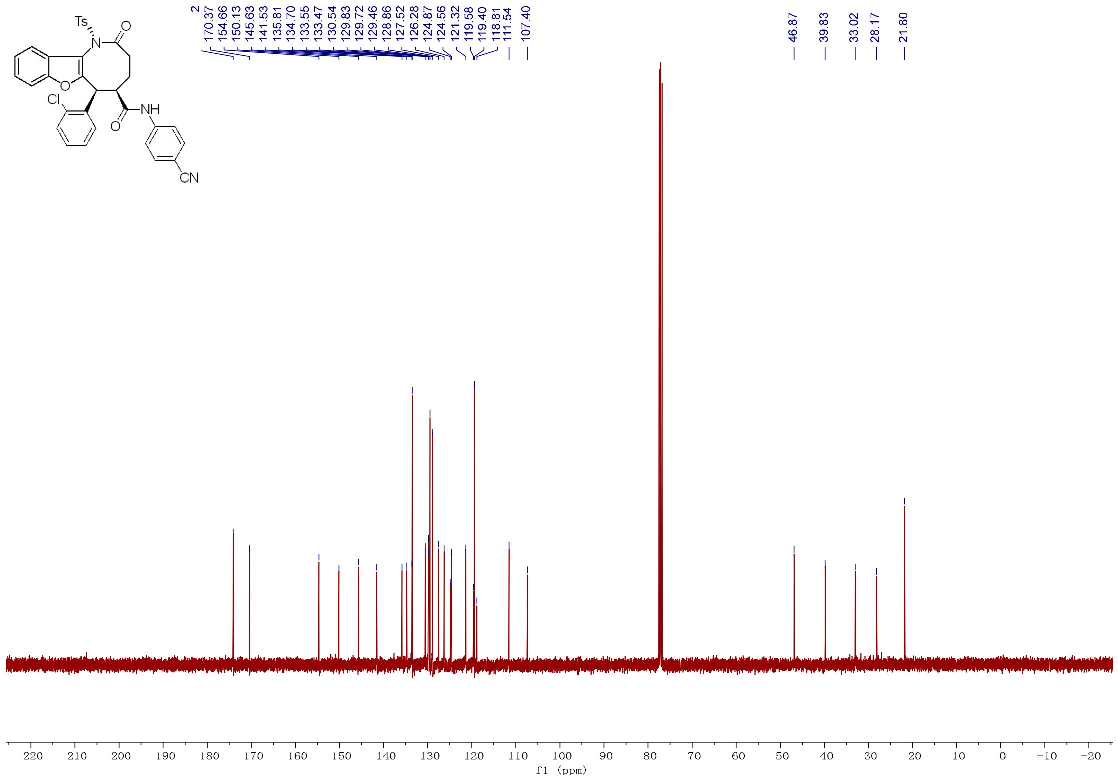

3qf, ^{1}H NMR (400 MHz, $CDCl_3$)

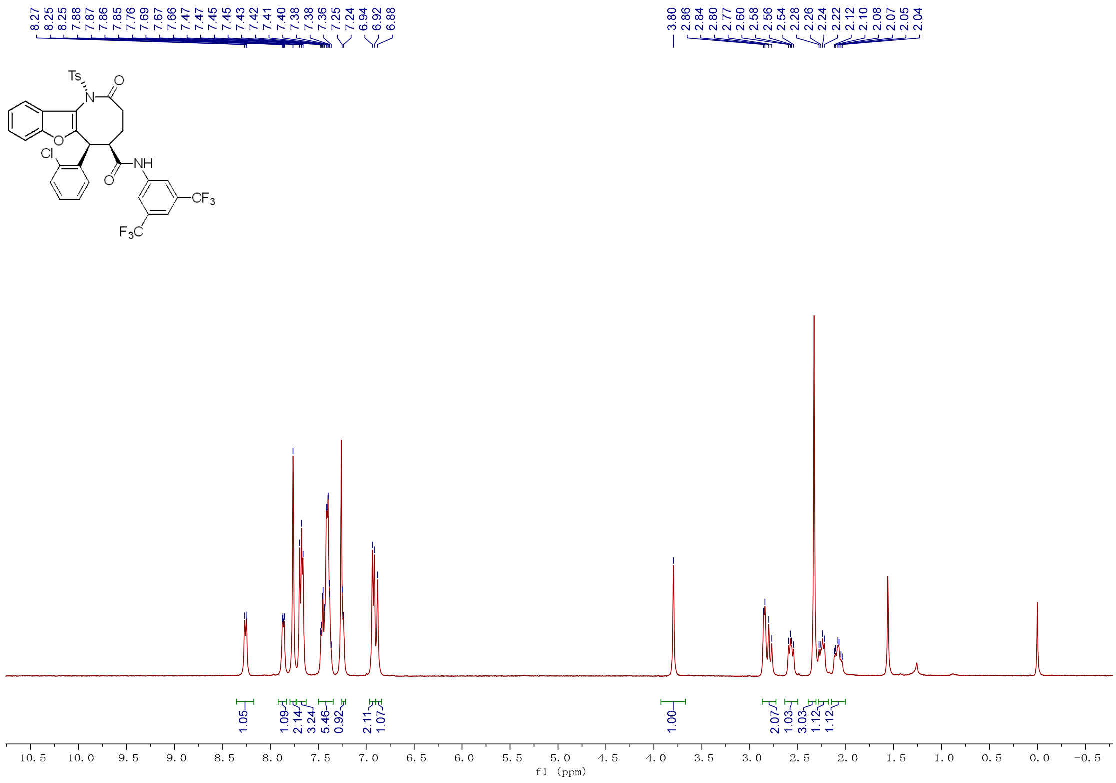

3qf, ^{13}C NMR (101 MHz, $CDCl_3$)

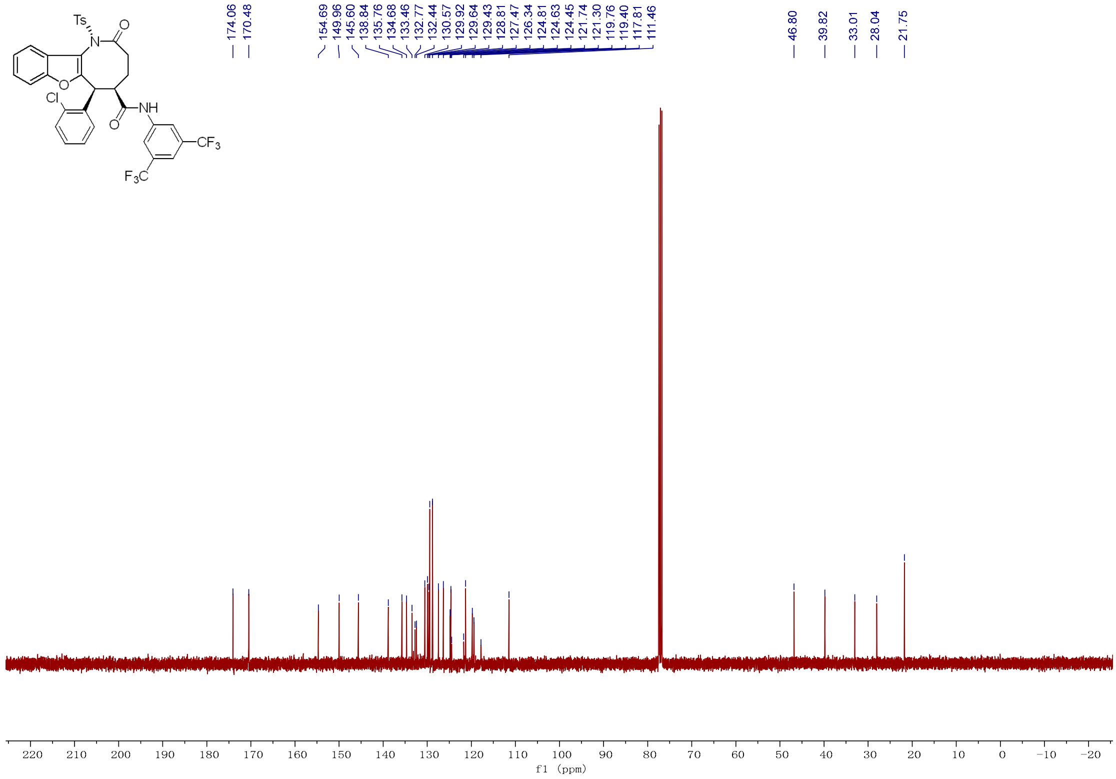

6, ^{1}H NMR (400 MHz, DMSO-d_6)

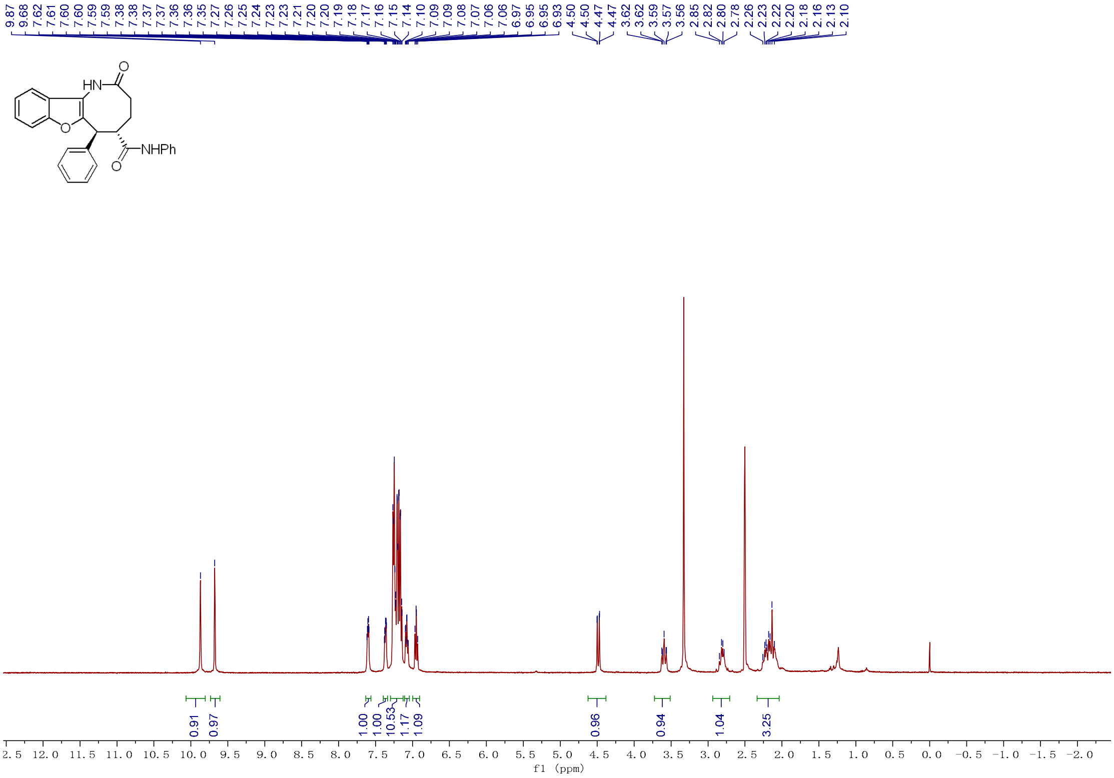

6, ^{13}C NMR (101 MHz, DMSO-d_6)

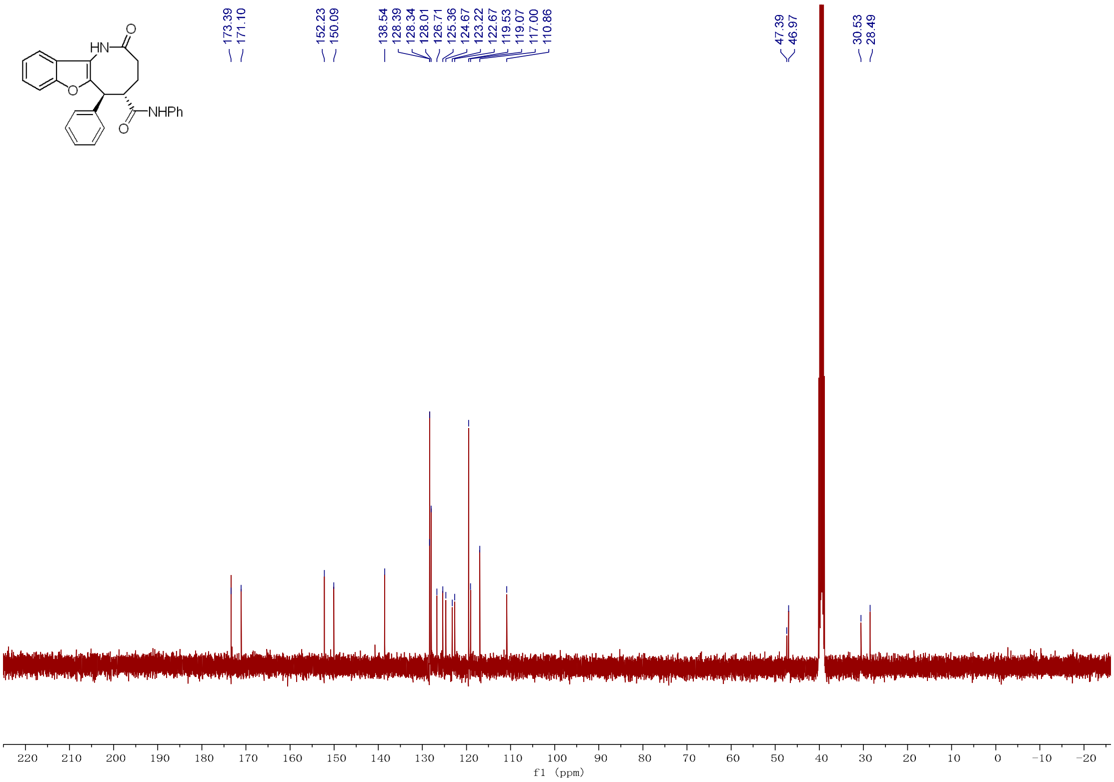

7, ^{1}H NMR (600 MHz, CDCl$_3$)

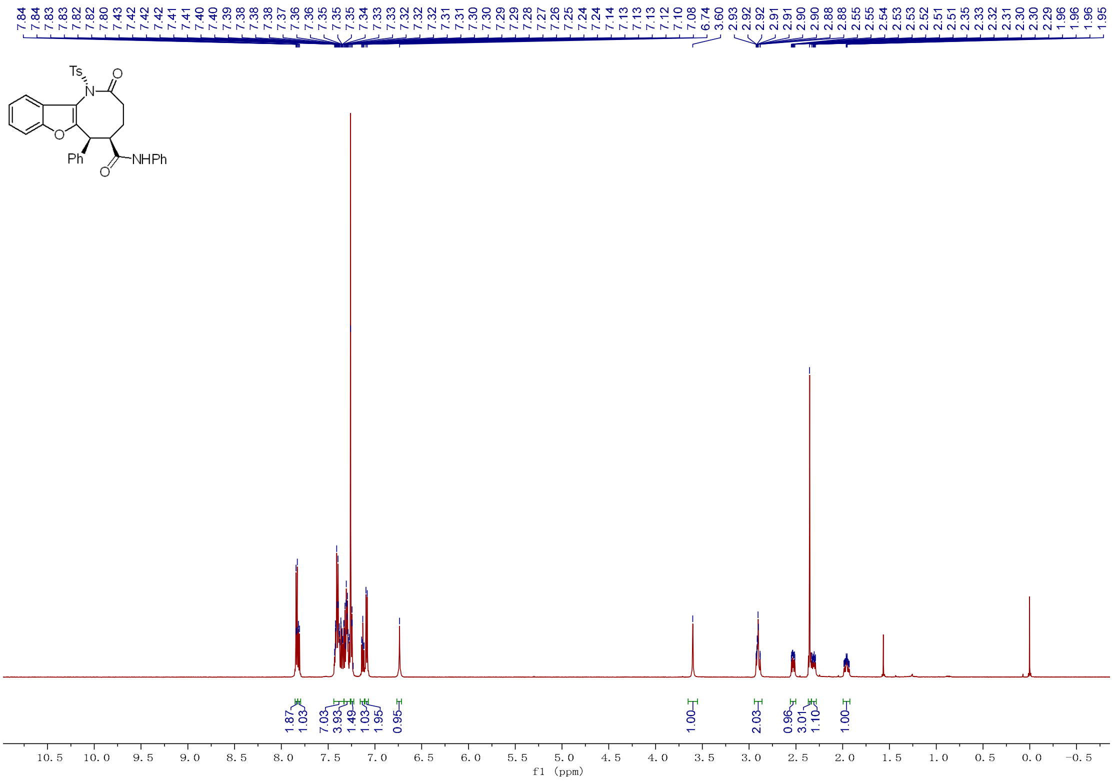

7, ^{13}C NMR (151 MHz, CDCl$_3$)

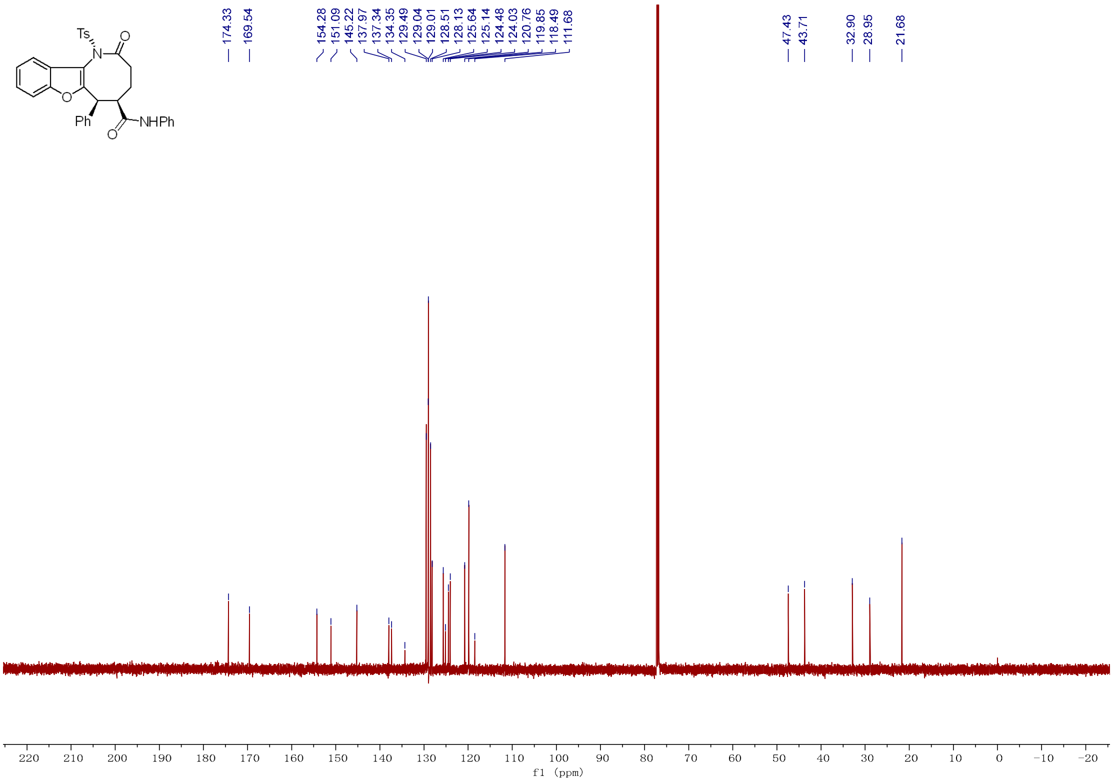

10. Optimization of catalytic asymmetric reaction conditions and HPLC Spectrum of 3aa

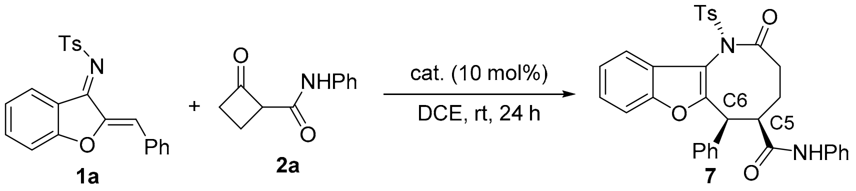

In a flame-dried Schlenk reaction tube equipped with a magnetic stir bar, the catalyst (0.01 mmol), **1a** (0.10 mmol, 37.5 mg) and **2a** (0.10 mmol, 19.8 mg) under N_2 were added, and freshly distilled DCE (2.0 mL) was added. The mixture was then stirred at rt for 24 h. Then, the reaction mixture was filtered through a pad of Celite, and the solvent was concentrated in vacuo. It was then purified by flash column chromatography to yield product **7** as a white solid. **^{1}H NMR** (600 MHz, $CDCl_3$) δ 7.85 – 7.83 (m, 2H), 7.82 – 7.79 (m, 1H), 7.44 – 7.33 (m, 7H), 7.33 – 7.26 (m, 4H), 7.26 – 7.23 (m, 1H), 7.16 – 7.11 (m, 1H), 7.09 (d, J = 8.0 Hz, 2H), 6.74 (s, 1H), 3.60 (s, 1H), 2.94 – 2.86 (m, 2H), 2.53 (ddd, J = 13.2, 7.9, 1.9 Hz, 1H), 2.35 (s, 3H), 2.34 – 2.28 (m, 1H), 1.96 (dddd, J = 14.3, 12.1, 5.5, 1.8 Hz, 1H). **^{13}C NMR** (151 MHz, $CDCl_3$) δ 174.3, 169.5, 154.3, 151.1, 145.2, 138.0, 137.3, 134.4, 129.5, 129.1, 129.0, 128.5, 128.1, 125.6, 125.1, 124.5, 124.0, 120.8, 119.9, 118.5, 111.7, 47.4, 43.7, 32.9, 29.0, 21.7.

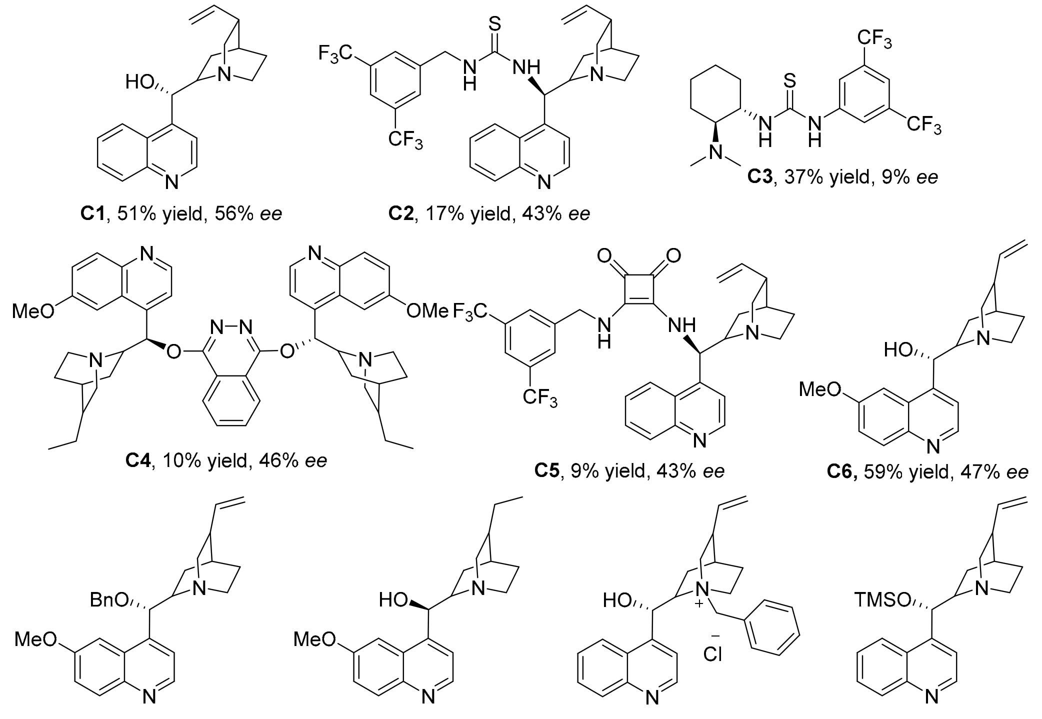

C1, 51% yield, 56% *ee*

C2, 17% yield, 43% *ee*

C3, 37% yield, 9% *ee*

C4, 10% yield, 46% *ee*

C5, 9% yield, 43% *ee*

C6, 59% yield, 47% *ee*

C7, 33% yield, 7% *ee*

C8, 37% yield, 43% *ee*

C9, trace

C10, trace

HPLC Spectrum of racemic 3aa

Method description: Chiralpak IF, Hexane/Isopropanol 80/20, 1 mL/min

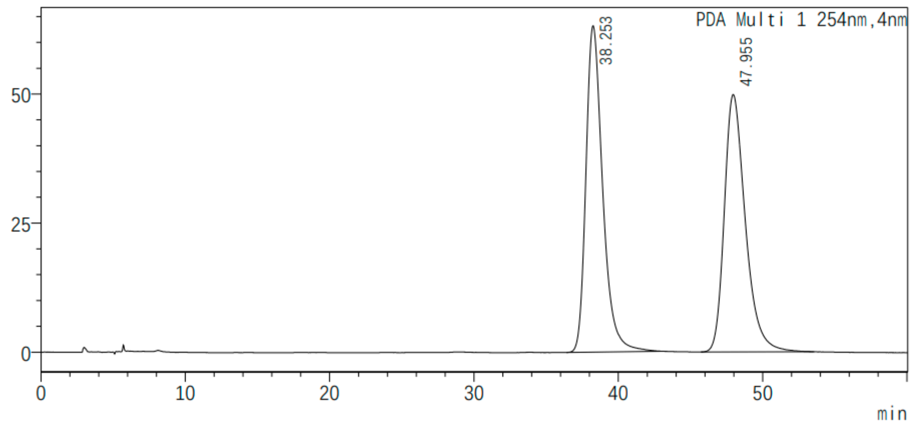

Peak#	Ret. Time	Area	Height	Area%	Height%
1	38.253	5111767	63227	50.236	55.900
2	47.955	5063733	49880	49.764	44.099
Total		10175500	113107	100.000	100.000

HPLC Spectrum of enantioenriched 3aa (prepared by using C1 as a catalyst)

Method description: Chiralpak IF, Hexane/Isopropanol 80/20, 1 mL/min

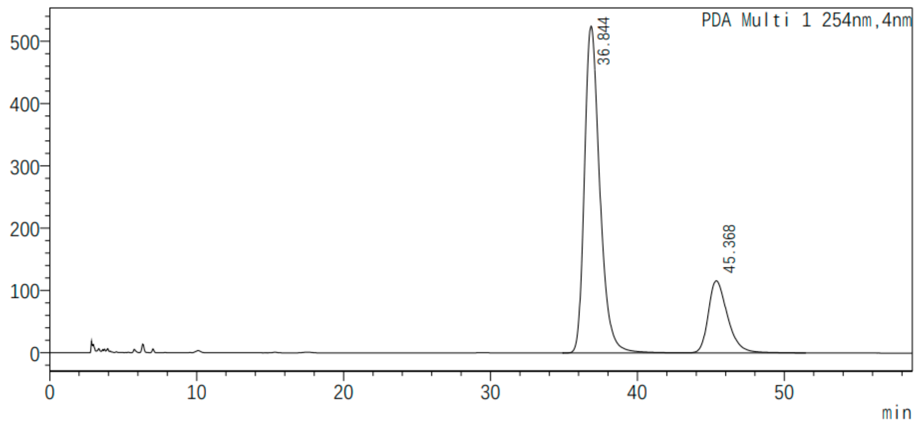

Peak#	Ret. Time	Area	Height	Area%	Height%
1	36.844	38186656	524798	77.953	81.913
2	45.368	10638990	115873	22.047	18.087
Total		48825646	640671	100.000	100.000
